# ECGene: A Literature‐Based Knowledgebase of Endometrial Cancer Genes

**DOI:** 10.1002/humu.22950

**Published:** 2016-01-13

**Authors:** Min Zhao, Yining Liu, Tracy A O'Mara

**Affiliations:** ^1^ School of Engineering, Faculty of Science, Health, Education and EngineeringUniversity of the Sunshine CoastQueensland4558Australia; ^2^ Genetics and Computational Biology DepartmentQIMR Berghofer Medical Research InstituteBrisbaneQueensland4006Australia

**Keywords:** endometrial cancer, database, cancer genomics, network analysis, systems biology, long noncoding RNA

## Abstract

Endometrial cancer (EC) ranks as the sixth common cancer for women worldwide. To better distinguish cancer subtypes and identify effective early diagnostic biomarkers, we need improved understanding of the biological mechanisms associated with EC dysregulated genes. Although there is a wealth of clinical and molecular information relevant to EC in the literature, there has been no systematic summary of EC‐implicated genes. In this study, we developed a literature‐based database ECGene (Endometrial Cancer Gene database) with comprehensive annotations. ECGene features manual curation of 414 genes from thousands of publications, results from eight EC gene expression datasets, precomputation of coexpressed long noncoding RNAs, and an EC‐implicated gene interactome. In the current release, we generated and comprehensively annotated a list of 458 EC‐implicated genes. We found the top‐ranked EC‐implicated genes are frequently mutated in The Cancer Genome Atlas (TCGA) tumor samples. Furthermore, systematic analysis of coexpressed lncRNAs provided insight into the important roles of lncRNA in EC development. ECGene has a user‐friendly Web interface and is freely available at http://ecgene.bioinfo‐minzhao.org/. As the first literature‐based online resource for EC, ECGene serves as a useful gateway for researchers to explore EC genetics.

## Introduction

Endometrial cancer (EC; cancer of the uterine corpus) is the sixth most commonly diagnosed cancer in women worldwide [Ferlay et al., 2015]. It is estimated that approximately 10,170 women will die from EC in the United States in 2015 (Siegel, et al., 2015). The majority of EC cases (53%) are from developed countries, with the highest incidence of this cancer reported in Northern America and Europe (Ferlay, et al., 2015). Accumulated histological and molecular evidence have classified EC as two distinct types (Bokhman, 1983; Dedes, et al., 2011), type I and type II carcinomas. Type I ECs, comprising approximately 80% of new EC cases, are of endometrioid histology and characterized by expression of the estrogen receptor and progesterone receptor. Type II ECs are nonendometrioid in histology, often with clear‐cell or serous papillary morphology (Dedes, et al., 2011). Type II EC tumors tend to be high grade, poorly differentiated tumors, and are associated with a more aggressive phenotype. Although type II tumors make up only ∼20% of all EC cases, they are responsible for a high proportion of EC‐related deaths (44%) (Hamilton, et al., 2006).

Despite the distinct morphologic and clinical features of the two EC subtypes, most EC patients are treated with the same chemotherapy, without an effective screening strategy in place to distinguish EC subtypes. Similar to other cancers, EC is remarkably heterogeneous at the cellular and molecular levels. The uncontrolled cell growth of EC results from promoter methylation (Tao and Freudenheim, 2010), copy‐number alteration of tumor suppressor genes and oncogenes (Zhao, et al., 2013e), and dysregulated expression of mRNA, microRNA (Banno, et al., 2013), and long noncoding RNAs (He, et al., 2014). Therefore, prioritization of genes reported to be associated with EC and understanding their function is critical to identify potential targets for precise treatment of this disease. Moreover, integration of known genes from published databases and the literature is necessary to evaluate their consistency. Here, we aim to develop the first literature‐based genetic resource for EC. With extensive annotations, ECGene will serve as a valuable platform allowing users to efficiently retrieve information about literature, gene regulation, and gene interactions of EC‐implicated genes.

## Materials and Methods

### Data Integration from Existing Bioinformatics Resources

To survey EC‐related genetic information, we performed extensive literature curation and data integration. EC‐implicated genes were first integrated from three databases: four genes from OMIM (Online Mendelian Inheritance in Man) (Amberger, et al., 2009); 113 unique genes from 149 published studies from GAD (The Genetic Association database) (Becker, et al., 2004); and 14 candidate genes identified from GWASCatalog (Welter, et al., 2014). Combined, we harvested a nonredundant EC‐implicated gene list of 127 human genes.

### Literature Collection and Gene Curation

A literature query of the GeneRif (Gene Reference Into Function) database was performed on Apr 5, 2015 to identify additional EC‐implicated genes and relevant publications. GeneRif is a collection of short descriptions about gene function in the Entrez Gene database (Lu, et al., 2007). By matching those GeneRif records with both endometrial and cancer keywords: ([endometrial OR uterine] AND [cancer OR tumor OR carcinoma]), we retrieved 845 PubMed abstracts. For further curation, we downloaded all 845 PubMed abstracts in a Medline format for manual review. Curation of EC‐implicated genes involved three steps: (1) grouping all 845 retrieved abstracts based on their sematic similarity, (2) extracting descriptions related to EC, and (3) collecting gene names from the descriptions and mapping the gene names to Entrez gene database. Here, we used Entrez gene IDs to cross‐link the same genes from different public bioinformatics databases. For example, in the sentence “*COX‐2 is upregulated in endometrial cancer and facilitates tumor growth via angiogenesis produced in associated with VEGF and TP. Specific inhibition of COX‐2 may be a useful therapeutic intervention in endometrial cancer*” (Fujiwaki, et al., 2002), the gene COX‐2 was one of the synonyms of PTGS2 in the current Entrez gene database. For those studies not on human, we mapped all the curated genes to their corresponding human homologous groups using NCBI HomoloGene database as implemented previously (Kong, et al., 2013; Zhao, et al., 2013d; Brown, et al., 2015).

In total, we identified 414 Entrez human genes from 747 PubMed abstracts. By integrating the 127 EC‐implicated genes identified from OMIM, GAD, and the GWASCatalog, we consolidated a list of 458 EC‐implicated genes, including 423 protein coding and 35 noncoding genes (Table S1). As shown in Supp. Figure S1, the majority of EC‐implicated genes were identified from our literature curation.

### Biological Annotation

To understand the biological function of the EC‐implicated genes, functional annotations for each gene was collected. Basic gene information such as gene names and DNA/protein sequences were integrated from the Entrez gene database (Sayers, et al., 2012). Cross‐links to the rate‐limiting enzyme database RLEdb (Zhao, et al., 2009) and text mining server iHOP (Fernandez, et al., 2007) were also provided. Associated biological pathways for genes were retrieved from BioCyc (Caspi, et al., 2014), KEGG Pathway (Kanehisa, et al., 2008), PID Curated (Schaefer, et al., 2009), PathLocdb (Zhao and Qu, 2010), PANTHER (Thomas, et al., 2003), and PID Reactome (Matthews, et al., 2009; Croft, et al., 2011). Additionally, reported associations with other diseases were integrated from the KEGG Disease database (Kanehisa, et al., 2010), Fundo (Du, et al., 2009; Osborne, et al., 2009), GAD (Becker, et al., 2004), NHGRI (Hindorff, et al., 2009), and OMIM (Sayers, et al., 2011). Post‐translational modifications and transcription factor regulation information was collected from dbPTM (Lee, et al., 2009) and the TRANSFAC database (Heinemeyer, et al., 1999), respectively. Digital gene expression information for 184 tumor samples and 84 normal tissues were integrated from BioGPS (Su, et al., 2004). Information about methylation sites were integrated from DiseaseMeth (Lv, et al., 2012), and protein–protein interactions from the Pathway Commons database (Cerami, et al., 2011).

### Network Reconstruction

To present a broader biological picture of EC, we collected all pathway‐based gene interactions associated with the 458 EC‐implicated genes, using a nonredundant pathway‐based human interactome from PathCommons. In total, the final pathway‐based interactome contained 3,629 genes and 36,034 connections. To create a subnetwork related to EC, we adopted the subnetwork extraction approach (Zhao, et al., 2013b). In this algorithm, all inputted EC‐implicated genes were mapped to the integrated interactome and the subnetwork extracted by connecting input genes by their shortest paths.

Unlike the functional exploration of a single gene, biological networks are often too complex to test the function on each element. However, because a few simple rules of topology relate to network function, the topological properties of a network are often used to characterize its global function (Barabasi and Oltvai, 2004). Here, the topological analyses were performed using NetworkAnalyzer (Smoot, et al., 2011). For each gene in the network, we calculated the number of connections for a node, also known as degree (Barabasi and Oltvai, 2004), and the short path, indicating the shortest steps for one gene to reach another (Barabasi and Oltvai, 2004). Cytoscape 2.8 was used for final network visualization (Smoot, et al., 2011).

### Coexpression with Long Noncoding RNAs in Matched Cancer Samples

To provide coexpression patterns of EC‐implicated genes with long noncoding RNAs (lncRNAs), we downloaded lncRNA expression data for The Cancer Genome Atlas (TCGA) EC patients (Cancer Genome Atlas Research Network et al., 2013) from the MiTranscriptome database (Iyer, et al., 2015). EC‐implicated gene expression data for the same patients were extracted from the TCGA data portal (https://tcga‐data.nci.nih.gov/tcga/tcgaHome2.jsp). The expression correlation between each of the 458 EC‐implicated genes and all 17,250 lncRNAs was assessed using Spearman's correlation scores.

The expression correlation scores and corresponding *P* values were calculated using R (version 2.14.0), and a false discovery rate (FDR) was applied to correct for multiple testing. For all EC‐implicated gene and lncRNA pairs, we required expression correlation scores greater than 0.3 and FDR‐adjusted *P* values less than 0.01.

### Functional Enrichment Analysis

The statistically representative pathways from KEGG and Reactome for each gene set were identified by ToppFun (Aerts, et al., 2006). In these functional enrichment analyses, all human protein‐coding genes were used as background. *P* values were corrected for multiple testing using Benjamini–Hochberg adjustments. Only human pathways with a corrected *P* value less than 0.01 for any gene set were considered significant.

## Results and Discussion

### Web Interface

Based on a systematic survey of EC‐related genes in public resources and literature, we have developed a user‐friendly Web interface to access all annotated information. Data and information in ECGene are stored in a MySQL relational database on a Linux server. Web‐based queries to the database are implemented in Perl scripts running in an Apache environment. ECGene allows users to quickly query by GeneID or gene symbol and run BLAST searches against all the sequences in ECGene. For advanced systems biology‐based studies, ECGene provides EC‐implicated gene nucleotide and protein sequences in a plain text format.

**Figure 1:**
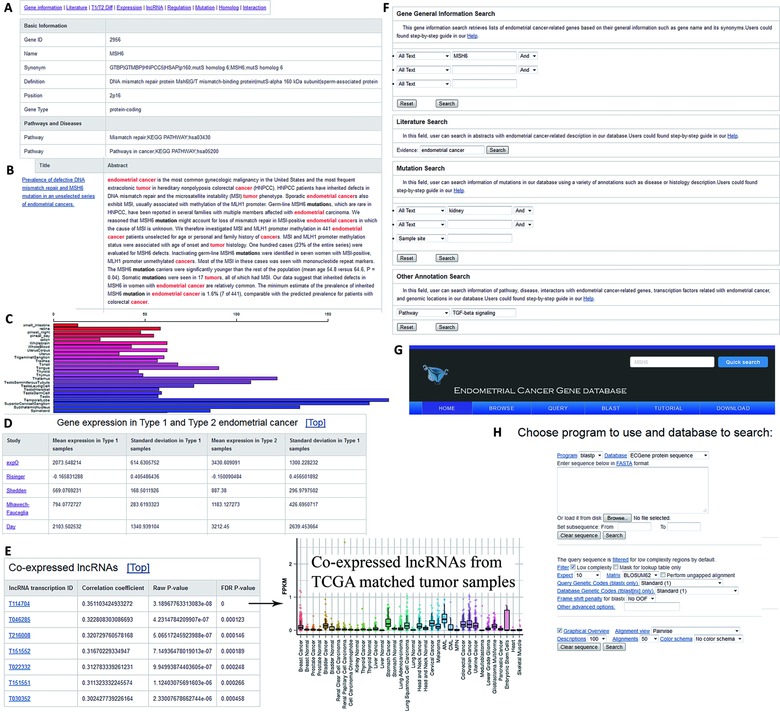
Web interface of ECGene database. **A**: Example of the general information provided for each endometrial cancer‐implicated gene. **B**: Example of gene‐supporting literature with keywords highlighted. **C**: Box plots of gene expression profiles in normal tissues. **D**: Gene expression data from endometrial cancer tissue expression studies. **E**: Coexpressed lncRNAs in TCGA endometrial cancer samples. **F**: Query interface for text search. **G**: The quick search interface for ECGene database. **H**: BLAST search interface for comparing query against all sequences in ECGene.

We classified gene annotations into nine groups: “Gene information,” “Literature,” “T1/T2 Diff,” “Expression,” “lncRNA,” “Regulation,” “Mutation,” “Homolog,” and “Interaction” (Fig. 1A). On the “Gene information” page, the official gene symbol, synonyms, gene functions, involved pathways and diseases, and nucleotide/protein sequences are listed. Cross‐references to other public bioinformatics databases such as iHOP, NCBI Entrez Gene, and KEGG pathway database are also included. The associated references for the queried gene are provided on the “Literature” page. Abstracts are provided and highlighted with cancer keywords (Fig. 1B). The “T1/T2 Diff” page displays type I and type II EC expression results from eight microarray expression studies (Fig. 1D; Supp. Table S2). In the “Expression” page, the queried gene's comprehensive expression profile from 84 normal and 184 tumor samples is provided (Fig. 1C). The “lncRNA” page presents correlation coefficients and associated *P* values for lncRNAs significantly correlated with the queried gene's expression using EC samples from TCGA (Fig. 1E). To view coexpressed lncRNA expression patterns across all TCGA tissue types in the MiTranscriptome database (Iyer, et al., 2015), users can click on the lncRNA ID. Interactions of the queried gene with transcription factors, its post‐translational modifications, and methylation information are found in the “Regulation” page. The “Mutation” page presents classified somatic mutations and mutation type (e.g., substitution, insertion, and deletion) from the most COSMIC database. The homologous sequences from other model species, integrated from NCBI HomoloGene database, are provided on the “Homolog” page. Finally, protein–protein interaction data from PathwayCommons database can be found on the “Interaction” page.

ECGene supports a variety of ways to browse EC‐implicated genes, including the highlighted KEGG map and chromosome location. KEGG maps have been marked with all EC‐implicated genes in color. Using chromosome browser, users can click on a chromosome of interest to access all EC‐implicated genes on that chromosome, with hyperlinks provided to access gene annotation pages.

Advanced search options are available to query ECGene by gene name or functional characteristics, including chromosome location, interaction partner, biological process, and disease (Fig. 1F). In addition, users can access curated references using a keyword search. A quick search function on the top right of each page is also provided to query by gene symbols or Gene IDs quickly (Fig. 1G).

In the BLAST page, users can evaluate sequence similarity by inputting their sequence of interest. The homologs of the input sequence are searched among the human genes in ECGene using BLAST. The sequence alignment options can be set by E‐value and identity score (Fig. 1H).

### Gene Ranking for all the Genes in ECGene and the Enriched Pathways for the Top‐Ranked Genes

We explored the relative importance, mutational frequency, and network properties of all 458 EC‐implicated genes identified from public resources and the literature. Since hundreds of genes are associated with EC, it is necessary to systematically prioritize the most informative genes. Using the ToppGene gene ranking tool (Aerts, et al., 2006), we ranked the relative importance for all the 423 protein‐coding genes in ECGene. To build a ranking model using ToppGene, we defined a training set with the 25 most reliable genes, that is, genes with 10 or more publications in our database (Supp. Table S3, training set). ToppGene then used the biological feature annotations from the training set to rank the remaining 399 genes. Biological feature annotations included gene coexpression, gene ontology evidence, literature mining data, pathway annotations, protein domain, and other sequence features. Finally, ToppGene combined all rankings, using order statistics, to construct a global ranking (Supp. Table S3, ranking result). Not surprisingly, some top‐ranked genes from the global ranking are genes that have been well‐studied for EC (e.g., both *EGFR* and *CDH1* have seven references in ECGene). However, other top‐ranked genes were only supported by a single publication regarding their abnormal expression in EC samples: *MUC8* (Hebbar, et al., 2005), *TARP* (Zhao, et al., 2013a), and *MAPK1* (Gong, et al., 2007). Further experimental validation of these three genes in EC may be deserved since their functional features are similar to those in the training set.

**Figure 2:**
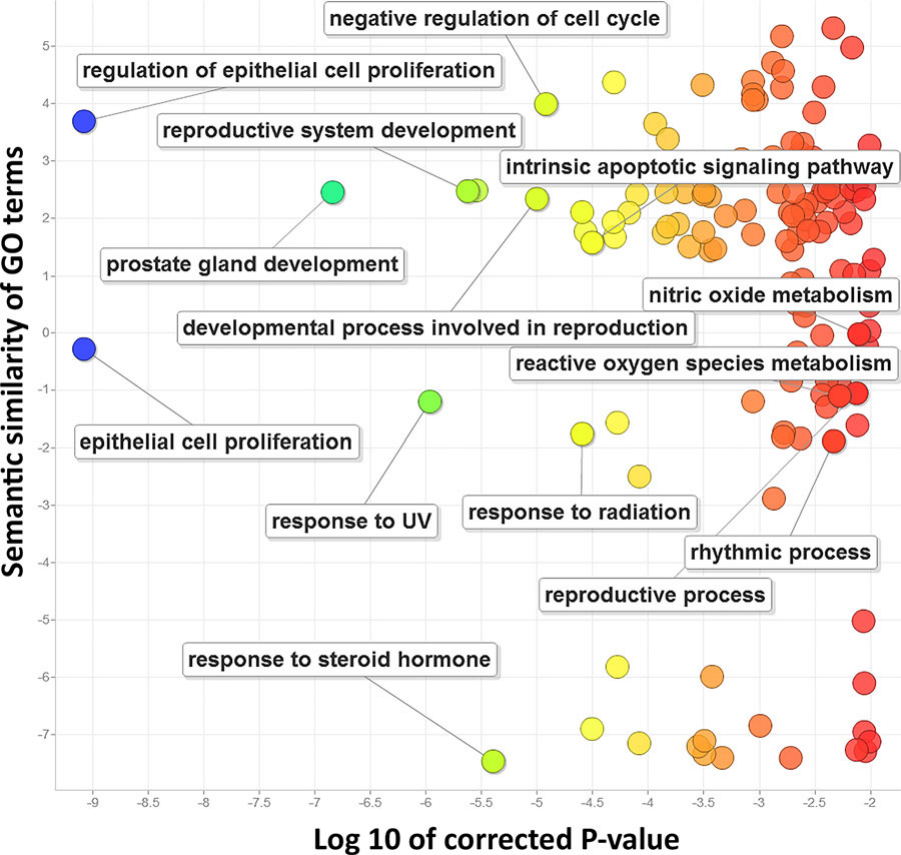
The enriched gene ontology terms of the top 100 ranked EC‐implicated genes. The X‐axis is the logarithm of corrected *P* values. The Y‐axis represents the similarity between the enriched gene ontology terms.

Functional enrichment analysis revealed that the top 100 ranked genes are enriched in cancer‐related and reproductive system biological processes according to gene ontology (Fig. 2). In particular, the top 100 ranked genes are associated with a number of key cancer pathways, such as the “Pathways in cancer” (corrected *P* value = 5.2 × 10^−5^) and “p53 pathway feedback loops 2” (corrected *P* value = 6.56 × 10^−5^) (Supp. Table S4). Interestingly, the most significantly enriched pathway is “Signaling by SCF‐KIT” (corrected *P* value = 2.9 × 10^−5^), a system that has been associated with carcinogenesis of the female genital tract (Inoue, et al., 1994). SCF (stem cell factor) is a cytokine that binds to the c‐KIT receptor (CD117) to promote cell growth. A recent study found targeting of the SCF/CD117 axis with imatinib‐sensitized EC cells to cisplatin, suggesting this axis may be a potential therapeutic target (Zhang, et al., 2014).

### Mutational Pattern for the Top 100 Ranked Genes in ECGene

**Figure 3:**
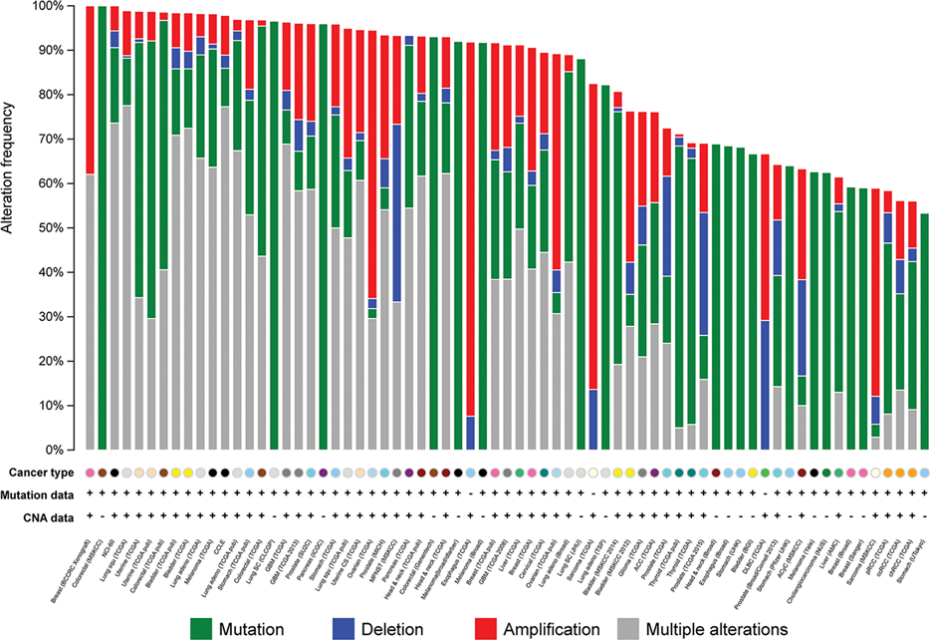
The mutational landscape for the top 99 ranked endometrial cancer genes in multiple cancers.

To identify highly mutated genes for further screening in relevant cancers, we systematically examined the somatic mutational pattern of the top 100 ranked genes in various cancer genomics datasets using cBio portal (Gao, et al., 2013). To avoid mutation bias from TP53 in the top 100 ranked gene set, it was excluded from mutational analysis across multiple cancers. As shown in Figure 3, the top 99 ranked EC‐implicated genes are frequently mutated in other cancer types such as colorectal and lung squamous cell carcinoma. These 99 genes are mutated in 98.9% patients of TCGA lung squamous cell carcinoma (176 individuals; Supp. Table S5), and among 98.8% patients of TCGA EC cohort (239 individuals) (Supp. Table S5). A similar prevalence of mutations (mutation rate over 90%) can be found in 31 other cancer mutation studies from 19 cancer types. This suggests that EC may have common genetic mutations with other carcinomas. Notably, those tumors adjacent to the uterus, such as bladder and colorectal cancers, have the most frequent mutational rate. This implies the adjacent tumors may have similar driver mutations in relation to the tissue of origin, an observation reported by the TCGA pan‐cancer mutational analysis (Kandoth, et al., 2013). Further exploration of the mutational pattern among 239 TCGA EC samples (Supp. Fig. S2 and Supp. Table S6) found the most frequently mutated genes (≥ 20% mutation frequency) are *PTEN, PIK3CA, PIK3R1, CTNNB1, TP53*, and *KRAS*. Taken together, these results may provide new avenues to develop novel therapeutics for those highly mutated genes in EC by repurposing the available drugs used in other cancers.

### Reconstruction of EC‐Implicated Interactome

**Figure 4:**
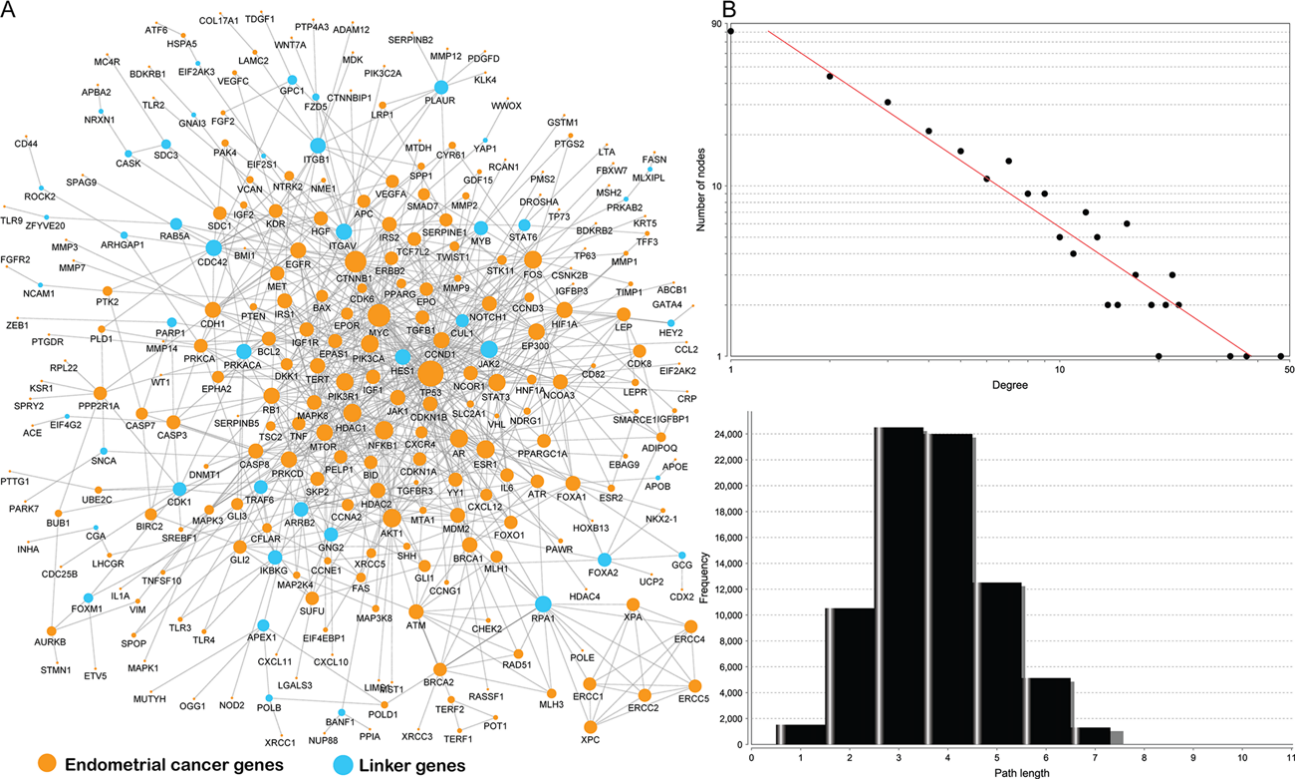
Reconstructed endometrial cancer map using protein–protein interaction data. **A**: The 246 genes in orange are genes from the core dataset in ECGene. The remaining 44 genes in blue are linker genes that bridge the 246 genes. **B**: The degree distribution. **C**: The short path length frequency.

To explore how the EC‐implicated genes interact with each other, we accessed large‐scale protein–protein interaction data. Only reliable interactions were used in this analysis to avoid the high level of noise (Cerami, et al., 2011). EC‐implicated genes were mapped to the pathway‐based interactome using a module searching method, as described previously (Zhao, et al., 2013b). The reconstructed EC pathway interactome comprised 290 genes and 769 gene–gene interactions (Fig. 4A). Of the 290 nodes, 246 are included in ECGene. The remaining 44 are linker genes to connect the EC genes to form a fully connected map.

Network topological analyses indicate the majority of genes are closely connected in the reconstructed map. This not only supports the accuracy of our data, but also implies that EC‐implicated genes can form a high‐density cellular module. As shown in Figure 4B, the majority of nodes have multiple connections, with only 89 of the 290 nodes in the reconstructed map displaying one connection. The degrees of all genes in our reconstructed EC map follow a power law distribution *P(k)∼k^−b^*, where *P(k)* is the probability that a gene interacts with other *k* genes and *b* is an exponent with an estimated value of 1.3. This means that our EC pathway map is more closely connected compared with all interactions in the human interactome, where most of nodes were sparsely connected with exponent *b* given as 2.9 (Jin, et al., 2013). Topological analysis on the shortest path distribution also confirmed dense modulation, with 75.9% of the node separated by only four steps (Fig. 4C; Supp. Table S7). With dense modularity, the hub nodes (defined as nodes with 20 or more connections) in this network may have prominent roles in the transfer of cellular signals by the shortest path. Eleven hub genes were identified in our network: *TP53* (47), *MYC* (37), *CTNNB1* (33), *AKT1* (23), *NFKB1* (23), *AR* (22), *ESR1* (22), *HDAC1* (22), *FOS* (21), *PIK3CA* (21), and *PIK3R1* (20) (Supp. Table S7). All 11 genes were present in ECGene. Annotation of these eleven genes found they are enriched in “cellular response to endogenous stimulus” and “enzyme‐linked receptor protein signaling pathway” (both corrected *P* values are 1.48×10^−11^) (Supp. Table S8). Moreover, 10 genes are involved in KEGG “Pathway in cancer” (corrected *P* value = 5.34 x ^−12^), “Colorectal cancer” (corrected *P* value = 1.94×10^−11^), “Prostate cancer” (corrected *P* value = 8.98×10^−11^), and “Endometrial cancer” (corrected *P* value = 3.39×10^−10^). In summary, our reconstructed map not only discovers multiple cancer pathways but also reveals a dense modular structure of previously unconnected EC signaling pathways.

### Coexpressed lncRNAs with EC‐Implicated Genes

To explore the role of lncRNAs in EC, we assessed the coexpression between 423 protein‐coding EC‐implicated genes with all 17,250 lncRNA transcripts from the MiTranscriptome database (Iyer, et al., 2015). We found 43 EC‐implicated genes that each had 100 or more positively coexpressed lncRNAs. As shown in Supp. Table S9, these genes are mainly enriched in the nucleoplasm (corrected *P* value = 1.97×10^−10^), associated with cell cycle (corrected *P* value = 1.83 10 ^−9^), and chromosome organization (corrected *P* value = 2.71×10^−9^). For example, *TERF1* was detected to be positively coexpressed with 539 lncRNAs in TCGA EC samples. The full name of this gene is Telomeric Repeat Binding Factor (NIMA‐Interacting) 1, which is present at telomeres throughout the cell cycle, inhibiting telomerase to interrupt the elongation of individual chromosome ends. These preliminary results imply coexpressed lncRNAs may be critical for tumorigenesis processes, such as cell cycle.

### Conclusions

In conclusion, we have consolidated 458 EC‐implicated genes (423 protein‐coding and 35 noncoding genes) by systematic data integration and literature curation. A user‐friendly Web interface was developed to access all gene, annotation, and literature information in our database, ECGene. We will continue to collect EC‐implicated genes from literatures in the future. In addition, we also plan to develop more tools that allow users to custom browse and search the Website.

## Supporting information

Disclaimer: Supplementary materials have been peer‐reviewed but not copyedited.

Supp. Figures S1 and S2.

Supp. Table S1. The basic annotations of curated 458 curated EC‐implicated genes.

Supp. Table S2. The eight genome‐wide expression datasets integrated in ECGene.

Supp. Table S3. The gene ranking results of EC‐implicated genes.

Supp. Table S4. The biological pathways enriched for EC‐implicated genes.

Supp. Table S5. The pan‐cancer mutational frequency for the top 99 ranked EC‐implicated genes.

Supp. Table S6. The mutational frequency for the top 100 ranked EC‐implicated genes in TCGA endometrial cancer samples.

Supp. Table S7. The network topological properties for the reconstructed EC‐implicated interactome.

Supp. Table S8. The enriched functional terms of 11 hub genes from the EC‐implicated interactome.

Supp. Table S9. The enriched functional terms of 43 EC‐implicated genes with 100 or more positively co‐expressed lncRNAs.
