# Supplementary material for: ECGene: A Literature‐Based Knowledgebase of Endometrial Cancer Genes
Source: Hum Mutat. 2016 Jan 13;37(4):337–43. doi: 10.1002/humu.22950 (PMC5066700; doi:10.1002/humu.22950)

Supporting Information for the Article:

ECGene: A literature-based knowledgebase of endometrial cancer genes

Min Zhao[1,2§], Yining Liu[1], Tracy A O'Mara[3]

[1] School of Engineering, Faculty of Science, Health, Education and Engineering, University of the Sunshine Coast, Maroochydore DC, Queensland, 4558, Australia.

[2] School of Biological Sciences, University of Queensland, St Lucia, Queensland, 4072, Australia.

[2] Genetics and Computational Biology Department, QIMR Berghofer Medical Research Institute, Brisbane, Queensland, 4006, Australia.

[§]Corresponding author:

Min Zhao

mzhao@usc.edu.au

Supp. Figures

Supp. Figure S1. Venn diagram displaying the number of genes mined from each data source.

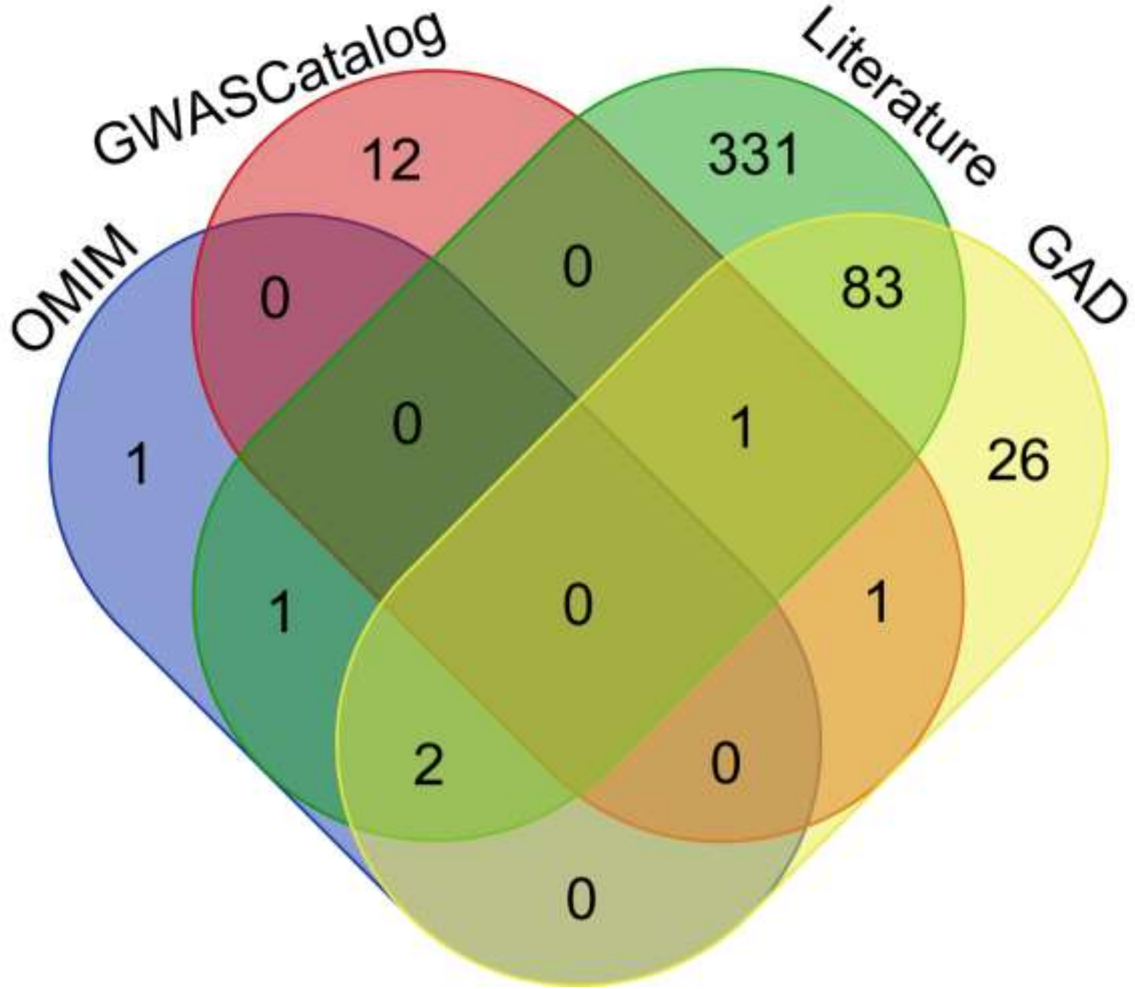

Supp. Figure S2. The mutational profile for the top 100 ranked EC-implicated genes in TCGA endometrial cancer samples.

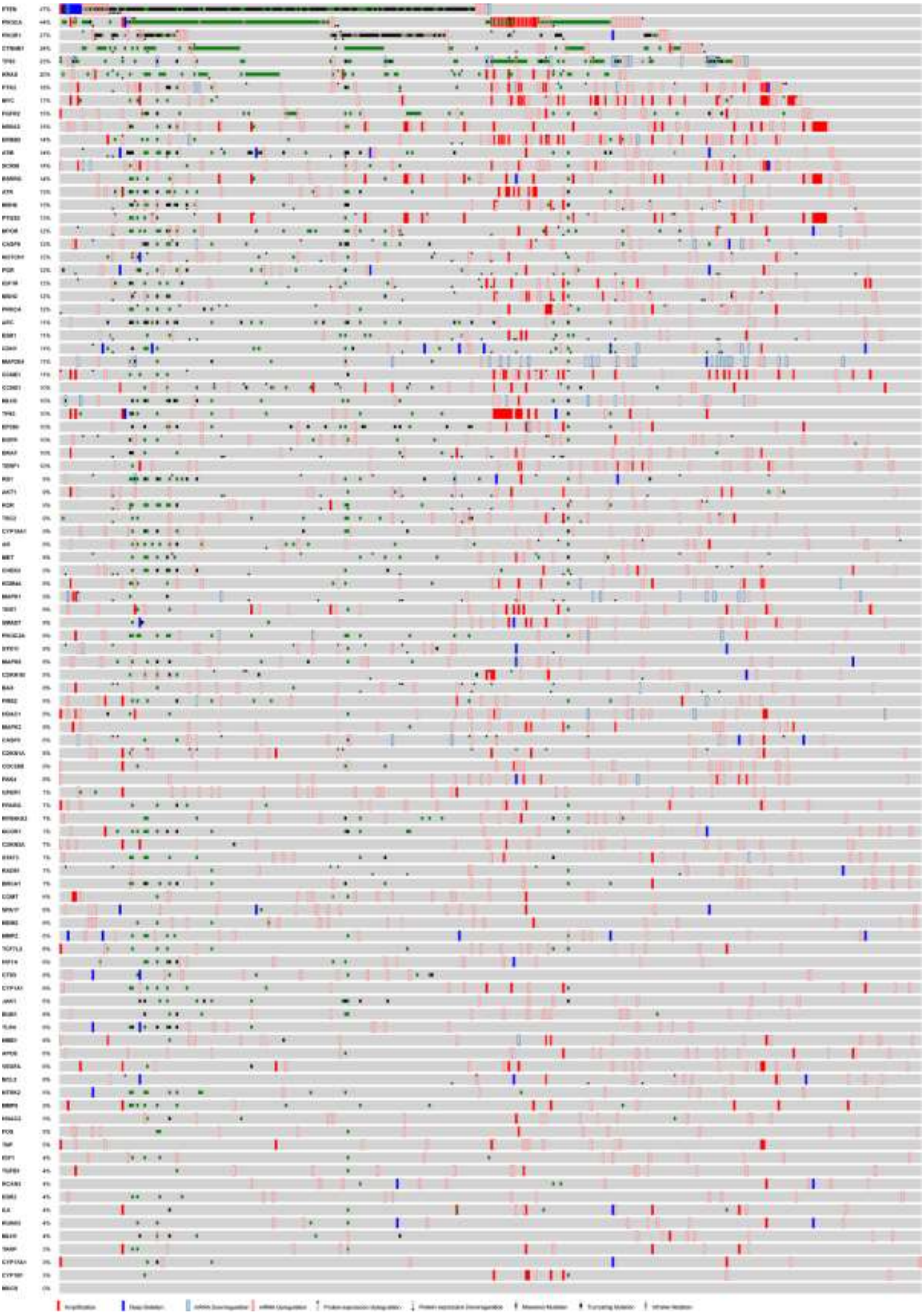

Supplement: Supplementary file 1 — Supp. Figures S1 and S2. [file HUMU-37-337-s001.pdf]
