# Supplementary material for: ECGene: A Literature‐Based Knowledgebase of Endometrial Cancer Genes
Source: Hum Mutat. 2016 Jan 13;37(4):337–43. doi: 10.1002/humu.22950 (PMC5066700; doi:10.1002/humu.22950)
Supplement: Supplementary file 2 — Supp. Table S1. The basic annotations of curated 458 curated EC‐implicated genes. [file HUMU-37-337-s003.docx]

| Supp. Table S1. The basic annotations of curated 458 curated EC-implicated genes. | | | | |
| --- | --- | --- | --- | --- |
|  | | | | |
|  | | | | |
| GeneID | **#of Evident** | **EvidenceDetail** | **#of PubMed** | **GeneSymb** |
| 5728 | 3 | GAD,OMIM,PubMed, | 43 | PTEN |
| 2956 | 3 | GAD,OMIM,PubMed, | 17 | MSH6 |
| 6928 | 3 | GWASCatalog,GAD,PubMed, | 2 | HNF1B |
| 7157 | 2 | GAD,PubMed, | 44 | TP53 |
| 2099 | 2 | GAD,PubMed, | 39 | ESR1 |
| 5241 | 2 | GAD,PubMed, | 26 | PGR |
| 4292 | 2 | GAD,PubMed, | 25 | MLH1 |
| 1588 | 2 | GAD,PubMed, | 24 | CYP19A1 |
| 4436 | 2 | GAD,PubMed, | 20 | MSH2 |
| 5290 | 2 | GAD,PubMed, | 19 | PIK3CA |
| 1586 | 2 | GAD,PubMed, | 19 | CYP17A1 |
| 3845 | 2 | GAD,PubMed, | 19 | KRAS |
| 2064 | 2 | GAD,PubMed, | 19 | ERBB2 |
| 1543 | 2 | GAD,PubMed, | 15 | CYP1A1 |
| 1545 | 2 | GAD,PubMed, | 15 | CYP1B1 |
| 7422 | 2 | GAD,PubMed, | 14 | VEGFA |
| 1499 | 2 | GAD,PubMed, | 13 | CTNNB1 |
| 207 | 2 | GAD,PubMed, | 12 | AKT1 |
| 367 | 2 | GAD,PubMed, | 11 | AR |
| 2100 | 2 | GAD,PubMed, | 11 | ESR2 |
| 4193 | 2 | GAD,PubMed, | 11 | MDM2 |
| 1312 | 2 | GAD,PubMed, | 10 | COMT |
| 4313 | 2 | GAD,PubMed, | 10 | MMP2 |
| 4318 | 2 | GAD,PubMed, | 10 | MMP9 |
| 3091 | 2 | GAD,PubMed, | 8 | HIF1A |
| 999 | 2 | OMIM,PubMed, | 7 | CDH1 |
| 1956 | 2 | GAD,PubMed, | 7 | EGFR |
| 673 | 2 | GAD,PubMed, | 6 | BRAF |
| 672 | 2 | GAD,PubMed, | 6 | BRCA1 |
| 54658 | 2 | GAD,PubMed, | 6 | UGT1A1 |
| 3292 | 2 | GAD,PubMed, | 5 | HSD17B1 |
| 595 | 2 | GAD,PubMed, | 5 | CCND1 |
| 7515 | 2 | GAD,PubMed, | 5 | XRCC1 |
| 1583 | 2 | GAD,PubMed, | 5 | CYP11A1 |
| 4595 | 2 | GAD,PubMed, | 5 | MUTYH |
| 3479 | 2 | GAD,PubMed, | 4 | IGF1 |
| 675 | 2 | GAD,PubMed, | 4 | BRCA2 |
| 3952 | 2 | GAD,PubMed, | 4 | LEP |
| 2950 | 2 | GAD,PubMed, | 4 | GSTP1 |
| 3569 | 2 | GAD,PubMed, | 4 | IL6 |
| 5468 | 2 | GAD,PubMed, | 4 | PPARG |
| 6783 | 2 | GAD,PubMed, | 4 | SULT1E1 |
| 4316 | 2 | GAD,PubMed, | 3 | MMP7 |
| 355 | 2 | GAD,PubMed, | 3 | FAS |
| 8202 | 2 | GAD,PubMed, | 3 | NCOA3 |
| 6817 | 2 | GAD,PubMed, | 3 | SULT1A1 |
| 4968 | 2 | GAD,PubMed, | 3 | OGG1 |
| 11200 | 2 | GAD,PubMed, | 3 | CHEK2 |
| 6462 | 2 | GAD,PubMed, | 3 | SHBG |
| 5395 | 2 | GAD,PubMed, | 3 | PMS2 |
| 7040 | 2 | GAD,PubMed, | 3 | TGFB1 |
| 3953 | 2 | GAD,PubMed, | 3 | LEPR |
| 2067 | 2 | GAD,PubMed, | 2 | ERCC1 |
| 79068 | 2 | GAD,PubMed, | 2 | FTO |
| 4524 | 2 | GAD,PubMed, | 2 | MTHFR |
| 3667 | 2 | GAD,PubMed, | 2 | IRS1 |
| 1401 | 2 | GAD,PubMed, | 2 | CRP |
| 1636 | 2 | GAD,PubMed, | 2 | ACE |
| 2952 | 2 | GAD,PubMed, | 2 | GSTT1 |
| 7015 | 2 | GAD,PubMed, | 2 | TERT |
| 7099 | 2 | GAD,PubMed, | 2 | TLR4 |
| 6770 | 2 | GAD,PubMed, | 2 | STAR |
| 4582 | 2 | GAD,PubMed, | 2 | MUC1 |
| 4314 | 2 | GAD,PubMed, | 2 | MMP3 |
| 25913 | 2 | GAD,PubMed, | 2 | POT1 |
| 5925 | 2 | GAD,PubMed, | 2 | RB1 |
| 3486 | 2 | GAD,PubMed, | 2 | IGFBP3 |
| 7077 | 2 | GAD,PubMed, | 2 | TIMP2 |
| 4312 | 2 | GAD,PubMed, | 2 | MMP1 |
| 6716 | 2 | GAD,PubMed, | 2 | SRD5A2 |
| 2944 | 2 | GAD,PubMed, | 2 | GSTM1 |
| 155 | 2 | GAD,PubMed, | 2 | ADRB3 |
| 4255 | 2 | GAD,PubMed, | 2 | MGMT |
| 6347 | 2 | GAD,PubMed, | 1 | CCL2 |
| 3484 | 2 | GAD,PubMed, | 1 | IGFBP1 |
| 3087 | 2 | GAD,PubMed, | 1 | HHEX |
| 7298 | 2 | GAD,PubMed, | 1 | TYMS |
| 841 | 2 | GAD,PubMed, | 1 | CASP8 |
| 6421 | 2 | GWASCatalog,GAD, | 1 | SFPQ |
| 8660 | 2 | GAD,PubMed, | 1 | IRS2 |
| 4830 | 2 | GAD,PubMed, | 1 | NME1 |
| 7364 | 2 | GAD,PubMed, | 1 | UGT2B7 |
| 5243 | 2 | GAD,PubMed, | 1 | ABCB1 |
| 472 | 2 | GAD,PubMed, | 1 | ATM |
| 7076 | 2 | GAD,PubMed, | 1 | TIMP1 |
| 545 | 2 | GAD,PubMed, | 1 | ATR |
| 840 | 2 | GAD,PubMed, | 1 | CASP7 |
| 3481 | 2 | GAD,PubMed, | 1 | IGF2 |
| 1029 | 1 | PubMed, | 13 | CDKN2A |
| 5743 | 1 | PubMed, | 13 | PTGS2 |
| 596 | 1 | PubMed, | 9 | BCL2 |
| 10406 | 1 | PubMed, | 9 | WFDC2 |
| 2263 | 1 | PubMed, | 7 | FGFR2 |
| 9370 | 1 | PubMed, | 7 | ADIPOQ |
| 7490 | 1 | PubMed, | 7 | WT1 |
| 1027 | 1 | PubMed, | 6 | CDKN1B |
| 2852 | 1 | PubMed, | 6 | GPER1 |
| 3480 | 1 | PubMed, | 6 | IGF1R |
| 1544 | 1 | GAD, | 6 | CYP1A2 |
| 3934 | 1 | PubMed, | 5 | LCN2 |
| 3309 | 1 | PubMed, | 5 | HSPA5 |
| 2475 | 1 | PubMed, | 5 | MTOR |
| 8837 | 1 | PubMed, | 4 | CFLAR |
| 94025 | 1 | PubMed, | 4 | MUC16 |
| 5737 | 1 | PubMed, | 4 | PTGFR |
| 4851 | 1 | PubMed, | 4 | NOTCH1 |
| 8842 | 1 | PubMed, | 4 | PROM1 |
| 5268 | 1 | PubMed, | 4 | SERPINB5 |
| 1026 | 1 | PubMed, | 4 | CDKN1A |
| 2308 | 1 | PubMed, | 4 | FOXO1 |
| 6387 | 1 | PubMed, | 4 | CXCL12 |
| 4288 | 1 | PubMed, | 3 | MKI67 |
| 648 | 1 | PubMed, | 3 | BMI1 |
| 5047 | 1 | PubMed, | 3 | PAEP |
| 3491 | 1 | PubMed, | 3 | CYR61 |
| 412 | 1 | PubMed, | 3 | STS |
| 5888 | 1 | PubMed, | 3 | RAD51 |
| 3589 | 1 | PubMed, | 3 | IL11 |
| 3206 | 1 | PubMed, | 3 | HOXA10 |
| 7424 | 1 | PubMed, | 3 | VEGFC |
| 5747 | 1 | PubMed, | 3 | PTK2 |
| 5426 | 1 | PubMed, | 3 | POLE |
| 23405 | 1 | PubMed, | 3 | DICER1 |
| 3169 | 1 | PubMed, | 3 | FOXA1 |
| 55281 | 1 | PubMed, | 3 | TMEM140 |
| 4790 | 1 | PubMed, | 3 | NFKB1 |
| 3576 | 1 | PubMed, | 3 | CXCL8 |
| 2119 | 1 | PubMed, | 3 | ETV5 |
| 6696 | 1 | PubMed, | 3 | SPP1 |
| 5424 | 1 | PubMed, | 3 | POLD1 |
| 6275 | 1 | PubMed, | 3 | S100A4 |
| 11186 | 1 | PubMed, | 3 | RASSF1 |
| 6774 | 1 | PubMed, | 3 | STAT3 |
| 2247 | 1 | PubMed, | 3 | FGF2 |
| 2678 | 1 | PubMed, | 2 | GGT1 |
| 3791 | 1 | PubMed, | 2 | KDR |
| 5578 | 1 | PubMed, | 2 | PRKCA |
| 11315 | 1 | PubMed, | 2 | PARK7 |
| 7049 | 1 | PubMed, | 2 | TGFBR3 |
| 2516 | 1 | PubMed, | 2 | NR5A1 |
| 1364 | 1 | PubMed, | 2 | CLDN4 |
| 7080 | 1 | PubMed, | 2 | NKX2-1 |
| 2706 | 1 | PubMed, | 2 | GJB2 |
| 8743 | 1 | PubMed, | 2 | TNFSF10 |
| 406987 | 1 | PubMed, | 2 | MIR204 |
| 861 | 1 | PubMed, | 2 | RUNX1 |
| 10643 | 1 | PubMed, | 2 | IGF2BP3 |
| 8626 | 1 | PubMed, | 2 | TP63 |
| 1048 | 1 | PubMed, | 2 | CEACAM5 |
| 2033 | 1 | PubMed, | 2 | EP300 |
| 3732 | 1 | PubMed, | 2 | CD82 |
| 9232 | 1 | PubMed, | 2 | PTTG1 |
| 324 | 1 | PubMed, | 2 | APC |
| 3925 | 1 | PubMed, | 2 | STMN1 |
| 1394 | 1 | PubMed, | 2 | CRHR1 |
| 4440 | 1 | PubMed, | 2 | MSI1 |
| 3036 | 1 | PubMed, | 2 | HAS1 |
| 7033 | 1 | PubMed, | 2 | TFF3 |
| 5478 | 1 | PubMed, | 2 | PPIA |
| 2146 | 1 | PubMed, | 2 | EZH2 |
| 56998 | 1 | PubMed, | 2 | CTNNBIP1 |
| 581 | 1 | PubMed, | 2 | BAX |
| 94031 | 1 | PubMed, | 2 | HTRA3 |
| 5295 | 1 | PubMed, | 2 | PIK3R1 |
| 3623 | 1 | PubMed, | 2 | INHA |
| 406937 | 1 | PubMed, | 2 | MIR145 |
| 27122 | 1 | PubMed, | 2 | DKK3 |
| 6382 | 1 | PubMed, | 2 | SDC1 |
| 6935 | 1 | PubMed, | 2 | ZEB1 |
| 6659 | 1 | PubMed, | 2 | SOX4 |
| 3626 | 1 | PubMed, | 2 | INHBC |
| 10855 | 1 | PubMed, | 2 | HPSE |
| 11191 | 1 | PubMed, | 2 | PTENP1 |
| 6934 | 1 | PubMed, | 2 | TCF7L2 |
| 9043 | 1 | PubMed, | 2 | SPAG9 |
| 406988 | 1 | PubMed, | 2 | MIR205 |
| 4915 | 1 | PubMed, | 2 | NTRK2 |
| 5654 | 1 | PubMed, | 2 | HTRA1 |
| 6513 | 1 | PubMed, | 2 | SLC2A1 |
| 27043 | 1 | PubMed, | 2 | PELP1 |
| 5518 | 1 | PubMed, | 2 | PPP2R1A |
| 7291 | 1 | PubMed, | 2 | TWIST1 |
| 4233 | 1 | PubMed, | 2 | MET |
| 9166 | 1 | PubMed, | 2 | EBAG9 |
| 7431 | 1 | PubMed, | 2 | VIM |
| 1978 | 1 | PubMed, | 2 | EIF4EBP1 |
| 7517 | 1 | GAD, | 2 | XRCC3 |
| 3082 | 1 | PubMed, | 2 | HGF |
| 100124700 | 1 | PubMed, | 2 | HOTAIR |
| 1574 | 1 | GAD, | 2 | CYP3A |
| 5580 | 1 | PubMed, | 2 | PRKCD |
| 699 | 1 | PubMed, | 2 | BUB1 |
| 960 | 1 | PubMed, | 2 | CD44 |
| 91746 | 1 | PubMed, | 2 | YTHDC1 |
| 7480 | 1 | PubMed, | 2 | WNT10B |
| 2104 | 1 | PubMed, | 2 | ESRRG |
| 6502 | 1 | PubMed, | 2 | SKP2 |
| 900 | 1 | PubMed, | 2 | CCNG1 |
| 22943 | 1 | PubMed, | 2 | DKK1 |
| 7852 | 1 | PubMed, | 2 | CXCR4 |
| 80310 | 1 | PubMed, | 2 | PDGFD |
| 4072 | 1 | PubMed, | 2 | EPCAM |
| 60561 | 1 | PubMed, | 1 | RINT1 |
| 406986 | 1 | PubMed, | 1 | MIR203A |
| 724031 | 1 | PubMed, | 1 | MIR661 |
| 8728 | 1 | PubMed, | 1 | ADAM19 |
| 7249 | 1 | PubMed, | 1 | TSC2 |
| 9682 | 1 | PubMed, | 1 | KDM4A |
| 85413 | 1 | PubMed, | 1 | SLC22A16 |
| 5979 | 1 | PubMed, | 1 | RET |
| 6279 | 1 | PubMed, | 1 | S100A8 |
| 1045 | 1 | PubMed, | 1 | CDX2 |
| 898 | 1 | PubMed, | 1 | CCNE1 |
| 3037 | 1 | PubMed, | 1 | HAS2 |
| 643623 | 1 | GWASCatalog, | 1 | LOC643623 |
| 7161 | 1 | GAD, | 1 | TP73 |
| 1890 | 1 | PubMed, | 1 | TYMP |
| 6434 | 1 | PubMed, | 1 | TRA2B |
| 407018 | 1 | PubMed, | 1 | MIR27A |
| 4549 | 1 | GAD, | 1 | RNR1 |
| 994 | 1 | PubMed, | 1 | CDC25B |
| 11123 | 1 | PubMed, | 1 | RCAN3 |
| 6605 | 1 | PubMed, | 1 | SMARCE1 |
| 1738 | 1 | PubMed, | 1 | DLD |
| 634 | 1 | PubMed, | 1 | CEACAM1 |
| 1786 | 1 | PubMed, | 1 | DNMT1 |
| 1021 | 1 | PubMed, | 1 | CDK6 |
| 8608 | 1 | PubMed, | 1 | RDH16 |
| 4035 | 1 | PubMed, | 1 | LRP1 |
| 9212 | 1 | PubMed, | 1 | AURKB |
| 4160 | 1 | PubMed, | 1 | MC4R |
| 3336 | 1 | PubMed, | 1 | HSPE1 |
| 8405 | 1 | PubMed, | 1 | SPOP |
| 30968 | 1 | PubMed, | 1 | STOML2 |
| 1969 | 1 | PubMed, | 1 | EPHA2 |
| 64127 | 1 | GAD, | 1 | NOD2 |
| 8473 | 1 | PubMed, | 1 | OGT |
| 3065 | 1 | PubMed, | 1 | HDAC1 |
| 2073 | 1 | GAD, | 1 | ERCC5 |
| 5054 | 1 | PubMed, | 1 | SERPINE1 |
| 4049 | 1 | GAD, | 1 | LTA |
| 3066 | 1 | PubMed, | 1 | HDAC2 |
| 4537 | 1 | GAD, | 1 | ND3 |
| 29102 | 1 | PubMed, | 1 | DROSHA |
| 57007 | 1 | PubMed, | 1 | ACKR3 |
| 79602 | 1 | PubMed, | 1 | ADIPOR2 |
| 4485 | 1 | PubMed, | 1 | MST1 |
| 7367 | 1 | PubMed, | 1 | UGT2B17 |
| 4609 | 1 | PubMed, | 1 | MYC |
| 3996 | 1 | PubMed, | 1 | LLGL1 |
| 6720 | 1 | PubMed, | 1 | SREBF1 |
| 348 | 1 | PubMed, | 1 | APOE |
| 51330 | 1 | PubMed, | 1 | TNFRSF12A |
| 8635 | 1 | GWASCatalog, | 1 | RNASET2 |
| 2587 | 1 | PubMed, | 1 | GALR1 |
| 7014 | 1 | GAD, | 1 | TERF2 |
| 4321 | 1 | PubMed, | 1 | MMP12 |
| 124 | 1 | PubMed, | 1 | ADH1A |
| 1308 | 1 | PubMed, | 1 | COL17A1 |
| 2057 | 1 | PubMed, | 1 | EPOR |
| 2194 | 1 | PubMed, | 1 | FASN |
| 7097 | 1 | GAD, | 1 | TLR2 |
| 83595 | 1 | PubMed, | 1 | SOX7 |
| 1462 | 1 | PubMed, | 1 | VCAN |
| 83729 | 1 | PubMed, | 1 | INHBE |
| 55353 | 1 | PubMed, | 1 | LAPTM4B |
| 9611 | 1 | PubMed, | 1 | NCOR1 |
| 7528 | 1 | PubMed, | 1 | YY1 |
| 2101 | 1 | PubMed, | 1 | ESRRA |
| 6416 | 1 | PubMed, | 1 | MAP2K4 |
| 3062 | 1 | PubMed, | 1 | HCRTR2 |
| 5599 | 1 | PubMed, | 1 | MAPK8 |
| 1848 | 1 | PubMed, | 1 | DUSP6 |
| 406969 | 1 | PubMed, | 1 | MIR194-1 |
| 10397 | 1 | PubMed, | 1 | NDRG1 |
| 1460 | 1 | PubMed, | 1 | CSNK2B |
| 780 | 1 | PubMed, | 1 | DDR1 |
| 637 | 1 | PubMed, | 1 | BID |
| 3490 | 1 | PubMed, | 1 | IGFBP7 |
| 890 | 1 | PubMed, | 1 | CCNA2 |
| 7098 | 1 | PubMed, | 1 | TLR3 |
| 5610 | 1 | PubMed, | 1 | EIF2AK2 |
| 9112 | 1 | PubMed, | 1 | MTA1 |
| 1012 | 1 | PubMed, | 1 | CDH13 |
| 4323 | 1 | PubMed, | 1 | MMP14 |
| 407046 | 1 | PubMed, | 1 | MIR9-1 |
| 54106 | 1 | GAD, | 1 | TLR9 |
| 55556 | 1 | GAD, | 1 | ENOSF1 |
| 255082 | 1 | PubMed, | 1 | CASC2 |
| 3987 | 1 | PubMed, | 1 | LIMS1 |
| 3855 | 1 | PubMed, | 1 | KRT7 |
| 3627 | 1 | PubMed, | 1 | CXCL10 |
| 5729 | 1 | GWASCatalog, | 1 | PTGDR |
| 9759 | 1 | PubMed, | 1 | HDAC4 |
| 3716 | 1 | PubMed, | 1 | JAK1 |
| 406940 | 1 | PubMed, | 1 | MIR148A |
| 727897 | 1 | PubMed, | 1 | MUC5B |
| 22926 | 1 | PubMed, | 1 | ATF6 |
| 407041 | 1 | PubMed, | 1 | MIR34B |
| 9670 | 1 | PubMed, | 1 | IPO13 |
| 10668 | 1 | PubMed, | 1 | CGRRF1 |
| 7508 | 1 | GAD, | 1 | XPC |
| 57758 | 1 | PubMed, | 1 | SCUBE2 |
| 1827 | 1 | PubMed, | 1 | RCAN1 |
| 10298 | 1 | PubMed, | 1 | PAK4 |
| 84525 | 1 | PubMed, | 1 | HOPX |
| 1395 | 1 | PubMed, | 1 | CRHR2 |
| 2737 | 1 | PubMed, | 1 | GLI3 |
| 896 | 1 | PubMed, | 1 | CCND3 |
| 2068 | 1 | GAD, | 1 | ERCC2 |
| 623 | 1 | PubMed, | 1 | BDKRB1 |
| 1365 | 1 | PubMed, | 1 | CLDN3 |
| 4567 | 1 | GAD, | 1 | TRNL1 |
| 407055 | 1 | PubMed, | 1 | MIR99A |
| 10253 | 1 | PubMed, | 1 | SPRY2 |
| 406918 | 1 | PubMed, | 1 | MIR129-2 |
| 2018 | 1 | PubMed, | 1 | EMX2 |
| 729230 | 1 | PubMed, | 1 | CCR2 |
| 2353 | 1 | PubMed, | 1 | FOS |
| 2296 | 1 | PubMed, | 1 | FOXC1 |
| 57650 | 1 | PubMed, | 1 | KIAA1524 |
| 84634 | 1 | PubMed, | 1 | KISS1R |
| 9817 | 1 | PubMed, | 1 | KEAP1 |
| 7078 | 1 | PubMed, | 1 | TIMP3 |
| 10392 | 1 | GAD, | 1 | NOD1 |
| 51741 | 1 | PubMed, | 1 | WWOX |
| 64073 | 1 | PubMed, | 1 | C19orf33 |
| 4780 | 1 | PubMed, | 1 | NFE2L2 |
| 836 | 1 | GAD, | 1 | CASP3 |
| 2736 | 1 | PubMed, | 1 | GLI2 |
| 2034 | 1 | PubMed, | 1 | EPAS1 |
| 4535 | 1 | GAD, | 1 | ND1 |
| 92140 | 1 | PubMed, | 1 | MTDH |
| 11156 | 1 | PubMed, | 1 | PTP4A3 |
| 54894 | 1 | PubMed, | 1 | RNF43 |
| 9510 | 1 | PubMed, | 1 | ADAMTS1 |
| 3552 | 1 | PubMed, | 1 | IL1A |
| 55799 | 1 | GWASCatalog, | 1 | CACNA2D3 |
| 4493 | 1 | PubMed, | 1 | MT1E |
| 329 | 1 | PubMed, | 1 | BIRC2 |
| 6196 | 1 | GWASCatalog, | 1 | RPS6KA2 |
| 5016 | 1 | PubMed, | 1 | OVGP1 |
| 8742 | 1 | PubMed, | 1 | TNFSF12 |
| 7428 | 1 | PubMed, | 1 | VHL |
| 7476 | 1 | PubMed, | 1 | WNT7A |
| 3177 | 1 | PubMed, | 1 | SLC29A2 |
| 406991 | 1 | PubMed, | 1 | MIR21 |
| 79923 | 1 | PubMed, | 1 | NANOG |
| 4192 | 1 | PubMed, | 1 | MDK |
| 54790 | 1 | PubMed, | 1 | TET2 |
| 100133941 | 1 | PubMed, | 1 | CD24 |
| 51684 | 1 | PubMed, | 1 | SUFU |
| 9622 | 1 | PubMed, | 1 | KLK4 |
| 112464 | 1 | PubMed, | 1 | PRKCDBP |
| 6794 | 1 | PubMed, | 1 | STK11 |
| 10891 | 1 | PubMed, | 1 | PPARGC1A |
| 321 | 1 | PubMed, | 1 | APBA2 |
| 8856 | 1 | PubMed, | 1 | NR1I2 |
| 624 | 1 | PubMed, | 1 | BDKRB2 |
| 3918 | 1 | PubMed, | 1 | LAMC2 |
| 100861523 | 1 | GWASCatalog, | 1 | RPS6KA2-AS1 |
| 3958 | 1 | PubMed, | 1 | LGALS3 |
| 8844 | 1 | PubMed, | 1 | KSR1 |
| 406920 | 1 | PubMed, | 1 | MIR130B |
| 406983 | 1 | PubMed, | 1 | MIR200A |
| 4092 | 1 | PubMed, | 1 | SMAD7 |
| 100129528 | 1 | PubMed, | 1 | MUC8 |
| 2072 | 1 | GAD, | 1 | ERCC4 |
| 2688 | 1 | PubMed, | 1 | GH1 |
| 5055 | 1 | PubMed, | 1 | SERPINB2 |
| 7257 | 1 | PubMed, | 1 | TSNAX |
| 406978 | 1 | PubMed, | 1 | MIR199B |
| 4927 | 1 | PubMed, | 1 | NUP88 |
| 3783 | 1 | PubMed, | 1 | KCNN4 |
| 7507 | 1 | GAD, | 1 | XPA |
| 27330 | 1 | PubMed, | 1 | RPS6KA6 |
| 4250 | 1 | PubMed, | 1 | SCGB2A2 |
| 6515 | 1 | PubMed, | 1 | SLC2A3 |
| 53340 | 1 | PubMed, | 1 | SPA17 |
| 23513 | 1 | PubMed, | 1 | SCRIB |
| 6692 | 1 | PubMed, | 1 | SPINT1 |
| 5286 | 1 | GAD, | 1 | PIK3C2A |
| 873 | 1 | PubMed, | 1 | CBR1 |
| 2346 | 1 | PubMed, | 1 | FOLH1 |
| 80351 | 1 | GAD, | 1 | TNKS2 |
| 574506 | 1 | PubMed, | 1 | MIR503 |
| 406968 | 1 | PubMed, | 1 | MIR193A |
| 5337 | 1 | PubMed, | 1 | PLD1 |
| 57504 | 1 | PubMed, | 1 | MTA3 |
| 7351 | 1 | GAD, | 1 | UCP2 |
| 5027 | 1 | PubMed, | 1 | P2RX7 |
| 4246 | 1 | PubMed, | 1 | SCGB2A1 |
| 9048 | 1 | PubMed, | 1 | ARTN |
| 25816 | 1 | PubMed, | 1 | TNFAIP8 |
| 51310 | 1 | PubMed, | 1 | SLC22A17 |
| 51738 | 1 | PubMed, | 1 | GHRL |
| 55294 | 1 | PubMed, | 1 | FBXW7 |
| 2272 | 1 | PubMed, | 1 | FHIT |
| 406900 | 1 | PubMed, | 1 | MIR106B |
| 6997 | 1 | PubMed, | 1 | TDGF1 |
| 2735 | 1 | PubMed, | 1 | GLI1 |
| 3852 | 1 | PubMed, | 1 | KRT5 |
| 84681 | 1 | PubMed, | 1 | HINT2 |
| 26136 | 1 | PubMed, | 1 | TES |
| 9202 | 1 | GWASCatalog, | 1 | ZMYM4 |
| 2739 | 1 | PubMed, | 1 | GLO1 |
| 3973 | 1 | PubMed, | 1 | LHCGR |
| 406984 | 1 | PubMed, | 1 | MIR200B |
| 6927 | 1 | PubMed, | 1 | HNF1A |
| 6373 | 1 | PubMed, | 1 | CXCL11 |
| 406943 | 1 | PubMed, | 1 | MIR152 |
| 5184 | 1 | PubMed, | 1 | PEPD |
| 10231 | 1 | PubMed, | 1 | RCAN2 |
| 6146 | 1 | PubMed, | 1 | RPL22 |
| 5594 | 1 | PubMed, | 1 | MAPK1 |
| 407004 | 1 | PubMed, | 1 | MIR22 |
| 9476 | 1 | PubMed, | 1 | NAPSA |
| 6623 | 1 | PubMed, | 1 | SNCG |
| 57535 | 1 | PubMed, | 1 | KIAA1324 |
| 1326 | 1 | PubMed, | 1 | MAP3K8 |
| 9518 | 1 | PubMed, | 1 | GDF15 |
| 5074 | 1 | PubMed, | 1 | PAWR |
| 100422916 | 1 | GWASCatalog, | 1 | MIR3201 |
| 158038 | 1 | GWASCatalog, | 1 | LINGO2 |
| 1230 | 1 | PubMed, | 1 | CCR1 |
| 3038 | 1 | PubMed, | 1 | HAS3 |
| 6678 | 1 | PubMed, | 1 | SPARC |
| 4512 | 1 | PubMed, | 1 | COX1 |
| 687 | 1 | PubMed, | 1 | KLF9 |
| 445347 | 1 | PubMed, | 1 | TARP |
| 2650 | 1 | PubMed, | 1 | GCNT1 |
| 7124 | 1 | PubMed, | 1 | TNF |
| 3161 | 1 | PubMed, | 1 | HMMR |
| 406947 | 1 | PubMed, | 1 | MIR155 |
| 1024 | 1 | PubMed, | 1 | CDK8 |
| 3590 | 1 | PubMed, | 1 | IL11RA |
| 6364 | 1 | PubMed, | 1 | CCL20 |
| 2626 | 1 | PubMed, | 1 | GATA4 |
| 407031 | 1 | PubMed, | 1 | MIR30C1 |
| 864 | 1 | PubMed, | 1 | RUNX3 |
| 8038 | 1 | GWASCatalog, | 1 | ADAM12 |
| 101928765 | 1 | GWASCatalog, | 1 | LOC101928765 |
| 2494 | 1 | PubMed, | 1 | NR5A2 |
| 5595 | 1 | PubMed, | 1 | MAPK3 |
| 406976 | 1 | PubMed, | 1 | MIR199A1 |
| 5063 | 1 | GAD, | 1 | PAK3 |
| 29028 | 1 | PubMed, | 1 | ATAD2 |
| 10202 | 1 | PubMed, | 1 | DHRS2 |
| 10653 | 1 | PubMed, | 1 | SPINT2 |
| 1515 | 1 | PubMed, | 1 | CTSV |
| 25817 | 1 | GWASCatalog, | 1 | FAM19A5 |
| 6469 | 1 | PubMed, | 1 | SHH |
| 2056 | 1 | PubMed, | 1 | EPO |
| 10481 | 1 | PubMed, | 1 | HOXB13 |
| 11065 | 1 | PubMed, | 1 | UBE2C |
| 7013 | 1 | GAD, | 1 | TERF1 |
| 3897 | 1 | PubMed, | 1 | L1CAM |
| 27030 | 1 | OMIM, | 0 | MLH3 |
