# Supplementary material for: ECGene: A Literature‐Based Knowledgebase of Endometrial Cancer Genes
Source: Hum Mutat. 2016 Jan 13;37(4):337–43. doi: 10.1002/humu.22950 (PMC5066700; doi:10.1002/humu.22950)
Supplement: Supplementary file 3 — Supp. Table S2. The eight genome‐wide expression datasets integrated in ECGene. [file HUMU-37-337-s004.docx]

| **Supp. Table S2. The eight genome-wide expression datasets integrated in ECGene.** | | | |  |  |
| --- | --- | --- | --- | --- | --- |
| **Study_Name** | **Number of Type1 samples** | **Number of Type2 samples** | **GEO Accession Number** | **PubMed ID** | **Platform** |
| Day | 79 | 12 | GSE17025 | 21619611 | Affymetrix HG U133 Plus 2.0 |
| Kharma | 51 | 12 | GSE56026 | 25267067 | Affymetrix HG U133 Plus 2.0 |
| Moreno-Bueno | 24 | 11 | NA | 14522886 | Oncochip, CNIO Genomic Unit |
| Mhawech-Fauceglia | 10 | 10 | GSE23518 | 21079744 | Illumina Human Ht-12 V3.0 |
| expO | 56 | 11 | GSE2109 | NA | Affymetrix HG U133 Plus 2.0 |
| MoMaTEC | 159 | 39 | E-MTAB-2532 | 24849812 | Agilent Human Genome 4x44K |
| Risinger | 19 | 16 | NA | 12517768 | Incyte Human GEM2 |
| TCGA | 41 | 13 | NA, from TCGA cancer browser | 23636398 | Agilent 244K Custom |
| Zorn | 7 | 24 | NA | 16166416 | UniGEM Custom |
| Shedden | 13 | 5 | NA | 15788657 | Affymetrix Hu6800 |
| Saidi | 17 | 3 | NA | 15247901 | Custom |
