# Supplementary material for: ECGene: A Literature‐Based Knowledgebase of Endometrial Cancer Genes
Source: Hum Mutat. 2016 Jan 13;37(4):337–43. doi: 10.1002/humu.22950 (PMC5066700; doi:10.1002/humu.22950)
Supplement: Supplementary file 4 — Supp. Table S3. The gene ranking results of EC‐implicated genes. [file HUMU-37-337-s005.docx]

| **Supp. Table S3. The gene ranking results of EC-implicated genes.** | | |
| --- | --- | --- |
| **Rank** | **GeneSymbol** | **Overall pValue** |
| 1 | EGFR | 1.27E-05 |
| 2 | PMS2 | 1.60E-05 |
| 3 | MET | 1.65E-05 |
| 4 | MUC8 | 2.25E-05 |
| 5 | TARP | 2.25E-05 |
| 6 | RB1 | 2.31E-05 |
| 7 | MAPK1 | 2.74E-05 |
| 8 | CDH1 | 2.79E-05 |
| 9 | APC | 2.81E-05 |
| 10 | EP300 | 2.84E-05 |
| 11 | MLH3 | 2.90E-05 |
| 12 | MTOR | 3.11E-05 |
| 13 | TCF7L2 | 3.89E-05 |
| 14 | STAT3 | 4.10E-05 |
| 15 | TP63 | 4.28E-05 |
| 16 | PPARG | 4.34E-05 |
| 17 | PIK3R1 | 4.47E-05 |
| 18 | HIF1A | 5.03E-05 |
| 19 | CHEK2 | 5.19E-05 |
| 20 | MAP2K4 | 5.55E-05 |
| 21 | HDAC2 | 5.82E-05 |
| 22 | HDAC1 | 6.00E-05 |
| 23 | NCOR1 | 6.67E-05 |
| 24 | BRAF | 7.02E-05 |
| 25 | TGFB1 | 7.08E-05 |
| 26 | CASP8 | 7.39E-05 |
| 27 | FGFR2 | 7.42E-05 |
| 28 | FOS | 7.45E-05 |
| 29 | MYC | 7.49E-05 |
| 30 | KDR | 7.87E-05 |
| 31 | PIK3C2A | 7.92E-05 |
| 32 | GPER1 | 8.08E-05 |
| 33 | MAPK8 | 8.14E-05 |
| 34 | BCL2 | 9.11E-05 |
| 35 | PTK2 | 9.33E-05 |
| 36 | MAPK3 | 9.55E-05 |
| 37 | RAD51 | 9.91E-05 |
| 38 | JAK1 | 1.01E-04 |
| 39 | BRCA1 | 1.16E-04 |
| 40 | TSC2 | 1.17E-04 |
| 41 | CCND1 | 1.22E-04 |
| 42 | IGF1R | 1.28E-04 |
| 43 | RCAN3 | 1.30E-04 |
| 44 | ATM | 1.33E-04 |
| 45 | ATR | 1.34E-04 |
| 46 | STK11 | 1.36E-04 |
| 47 | PRKCA | 1.42E-04 |
| 48 | CTSV | 1.43E-04 |
| 49 | BUB1 | 1.44E-04 |
| 50 | NTRK2 | 1.47E-04 |
| 51 | NME1 | 1.51E-04 |
| 52 | SCRIB | 1.60E-04 |
| 53 | CDKN1B | 1.62E-04 |
| 54 | APOE | 1.69E-04 |
| 55 | ESRRG | 1.74E-04 |
| 56 | CASP3 | 1.76E-04 |
| 57 | TERT | 1.76E-04 |
| 58 | BAX | 1.84E-04 |
| 59 | IGF1 | 1.88E-04 |
| 60 | RPS6KA2 | 1.89E-04 |
| 61 | TLR4 | 1.92E-04 |
| 62 | TERF1 | 2.11E-04 |
| 63 | CCNE1 | 2.18E-04 |
| 64 | TNF | 2.34E-04 |
| 65 | KDM4A | 2.35E-04 |
| 66 | CDKN1A | 2.38E-04 |
| 67 | PAK4 | 2.44E-04 |
| 68 | NOTCH1 | 2.52E-04 |
| 69 | SPA17 | 2.52E-04 |
| 70 | IL6 | 2.54E-04 |
| 71 | RUNX3 | 2.64E-04 |
| 72 | SMAD7 | 2.73E-04 |
| 73 | NR5A2 | 2.98E-04 |
| 74 | CDC25B | 3.00E-04 |
| 75 | MMP3 | 3.10E-04 |
| 76 | SOX7 | 3.12E-04 |
| 77 | XRCC3 | 3.14E-04 |
| 78 | KSR1 | 3.15E-04 |
| 79 | EZH2 | 3.16E-04 |
| 80 | STOML2 | 3.20E-04 |
| 81 | HSPE1 | 3.21E-04 |
| 82 | CCND3 | 3.32E-04 |
| 83 | ATF6 | 3.32E-04 |
| 84 | CCL2 | 3.37E-04 |
| 85 | WT1 | 3.38E-04 |
| 86 | CYP1A2 | 3.54E-04 |
| 87 | PRKCD | 3.55E-04 |
| 88 | CXCL8 | 3.59E-04 |
| 89 | NFE2L2 | 3.65E-04 |
| 90 | BRCA2 | 3.67E-04 |
| 91 | IGF2 | 3.75E-04 |
| 92 | ACE | 3.76E-04 |
| 93 | FGF2 | 3.76E-04 |
| 94 | SMARCE1 | 3.81E-04 |
| 95 | GATA4 | 3.82E-04 |
| 96 | HOPX | 3.82E-04 |
| 97 | TNKS2 | 3.92E-04 |
| 98 | GLI3 | 4.21E-04 |
| 99 | NCOA3 | 4.25E-04 |
| 100 | FAS | 4.31E-04 |
| 101 | GSTP1 | 4.33E-04 |
| 102 | RPS6KA6 | 4.34E-04 |
| 103 | CDK8 | 4.36E-04 |
| 104 | YY1 | 4.48E-04 |
| 105 | POLD1 | 4.69E-04 |
| 106 | ERCC2 | 4.70E-04 |
| 107 | NR1I2 | 4.79E-04 |
| 108 | VHL | 4.82E-04 |
| 109 | HSPA5 | 4.82E-04 |
| 110 | L1CAM | 4.85E-04 |
| 111 | HNF1A | 4.90E-04 |
| 112 | DHRS2 | 4.92E-04 |
| 113 | PPARGC1A | 4.98E-04 |
| 114 | MMP14 | 4.99E-04 |
| 115 | MKI67 | 5.00E-04 |
| 116 | CD44 | 5.11E-04 |
| 117 | PRKCDBP | 5.16E-04 |
| 118 | TSNAX | 5.17E-04 |
| 119 | MMP1 | 5.20E-04 |
| 120 | CYP11A1 | 5.21E-04 |
| 121 | NFKB1 | 5.32E-04 |
| 122 | FTO | 5.38E-04 |
| 123 | ADAM19 | 5.40E-04 |
| 124 | RINT1 | 5.43E-04 |
| 125 | SDC1 | 5.46E-04 |
| 126 | ABCB1 | 5.72E-04 |
| 127 | EPCAM | 5.79E-04 |
| 128 | TLR9 | 5.81E-04 |
| 129 | WNT7A | 5.82E-04 |
| 130 | IPO13 | 5.86E-04 |
| 131 | MTDH | 5.87E-04 |
| 132 | CCR2 | 5.97E-04 |
| 133 | ATAD2 | 6.02E-04 |
| 134 | HTRA3 | 6.06E-04 |
| 135 | TIMP1 | 6.13E-04 |
| 136 | SPP1 | 6.19E-04 |
| 137 | CTNNBIP1 | 6.24E-04 |
| 138 | LHCGR | 6.26E-04 |
| 139 | RET | 6.26E-04 |
| 140 | CRHR2 | 6.37E-04 |
| 141 | SREBF1 | 6.38E-04 |
| 142 | RNF43 | 6.40E-04 |
| 143 | EPHA2 | 6.43E-04 |
| 144 | FOXO1 | 6.47E-04 |
| 145 | AURKB | 6.59E-04 |
| 146 | PARK7 | 6.65E-04 |
| 147 | SPRY2 | 6.73E-04 |
| 148 | DKK3 | 6.76E-04 |
| 149 | DICER1 | 6.89E-04 |
| 150 | PELP1 | 6.99E-04 |
| 151 | LCN2 | 7.00E-04 |
| 152 | IRS1 | 7.23E-04 |
| 153 | MAP3K8 | 7.33E-04 |
| 154 | FOXC1 | 7.60E-04 |
| 155 | SKP2 | 7.64E-04 |
| 156 | LRP1 | 7.79E-04 |
| 157 | SOX4 | 7.87E-04 |
| 158 | YTHDC1 | 7.88E-04 |
| 159 | TLR2 | 7.91E-04 |
| 160 | SRD5A2 | 7.94E-04 |
| 161 | FHIT | 7.97E-04 |
| 162 | CGRRF1 | 8.04E-04 |
| 163 | TYMS | 8.04E-04 |
| 164 | ZMYM4 | 8.06E-04 |
| 165 | ESRRA | 8.17E-04 |
| 166 | GSTM1 | 8.28E-04 |
| 167 | CDX2 | 8.34E-04 |
| 168 | UCP2 | 8.38E-04 |
| 169 | MTHFR | 8.44E-04 |
| 170 | UGT1A1 | 8.48E-04 |
| 171 | HDAC4 | 8.55E-04 |
| 172 | BIRC2 | 8.60E-04 |
| 173 | PLD1 | 8.68E-04 |
| 174 | CRP | 8.71E-04 |
| 175 | RUNX1 | 8.74E-04 |
| 176 | WFDC2 | 8.79E-04 |
| 177 | S100A4 | 8.87E-04 |
| 178 | MUTYH | 9.06E-04 |
| 179 | ENOSF1 | 9.08E-04 |
| 180 | BID | 9.26E-04 |
| 181 | EIF4EBP1 | 9.32E-04 |
| 182 | DNMT1 | 9.35E-04 |
| 183 | BDKRB2 | 9.45E-04 |
| 184 | CFLAR | 9.54E-04 |
| 185 | SUFU | 9.60E-04 |
| 186 | ERCC1 | 9.66E-04 |
| 187 | CDK6 | 9.76E-04 |
| 188 | HHEX | 9.97E-04 |
| 189 | NR5A1 | 9.98E-04 |
| 190 | EPO | 0.001002179 |
| 191 | TIMP2 | 0.001009774 |
| 192 | CXCL10 | 0.001013482 |
| 193 | POLE | 0.001026395 |
| 194 | TP73 | 0.001044005 |
| 195 | BMI1 | 0.001045256 |
| 196 | EBAG9 | 0.001049999 |
| 197 | CXCR4 | 0.001056835 |
| 198 | EIF2AK2 | 0.001058355 |
| 199 | SPAG9 | 0.001059485 |
| 200 | HINT2 | 0.001071338 |
| 201 | FOXA1 | 0.001076562 |
| 202 | KIAA1524 | 0.001078688 |
| 203 | XPC | 0.001080472 |
| 204 | SCUBE2 | 0.001123235 |
| 205 | CXCL12 | 0.001143193 |
| 206 | ADAM12 | 0.001151974 |
| 207 | LGALS3 | 0.001152416 |
| 208 | CSNK2B | 0.001155134 |
| 209 | IL1A | 0.00116278 |
| 210 | MTA3 | 0.001173013 |
| 211 | HAS2 | 0.001175075 |
| 212 | ADIPOQ | 0.00117563 |
| 213 | GLI2 | 0.00119164 |
| 214 | XRCC1 | 0.001192114 |
| 215 | PPP2R1A | 0.001205786 |
| 216 | FASN | 0.00120693 |
| 217 | LEPR | 0.001216512 |
| 218 | C19orf33 | 0.001220325 |
| 219 | FBXW7 | 0.001229299 |
| 220 | HOXA10 | 0.001236844 |
| 221 | CCNG1 | 0.001256094 |
| 222 | MMP7 | 0.001269075 |
| 223 | HGF | 0.001282292 |
| 224 | UGT2B7 | 0.001298194 |
| 225 | RDH16 | 0.001311516 |
| 226 | TWIST1 | 0.001353419 |
| 227 | CRHR1 | 0.001380937 |
| 228 | ADH1A | 0.001390092 |
| 229 | IGFBP3 | 0.001394217 |
| 230 | CCNA2 | 0.001412415 |
| 231 | WWOX | 0.001414514 |
| 232 | SHBG | 0.001423144 |
| 233 | SLC22A17 | 0.001423385 |
| 234 | RASSF1 | 0.001433601 |
| 235 | KCNN4 | 0.001437564 |
| 236 | ADAMTS1 | 0.001444273 |
| 237 | SPARC | 0.001450555 |
| 238 | HNF1B | 0.00145157 |
| 239 | POT1 | 0.001451839 |
| 240 | ZEB1 | 0.001484032 |
| 241 | SERPINE1 | 0.001487657 |
| 242 | NDRG1 | 0.001489685 |
| 243 | SPINT1 | 0.001494944 |
| 244 | LTA | 0.001509438 |
| 245 | S100A8 | 0.001537235 |
| 246 | SHH | 0.001567479 |
| 247 | VIM | 0.001614709 |
| 248 | ERCC4 | 0.001615848 |
| 249 | CASP7 | 0.001634078 |
| 250 | GSTT1 | 0.001651649 |
| 251 | SLC2A1 | 0.001665196 |
| 252 | HSD17B1 | 0.001677243 |
| 253 | OGT | 0.001682952 |
| 254 | SCGB2A1 | 0.001719384 |
| 255 | CLDN4 | 0.001723618 |
| 256 | MMP12 | 0.001747015 |
| 257 | SULT1A1 | 0.001774261 |
| 258 | IRS2 | 0.001781388 |
| 259 | CEACAM1 | 0.001809162 |
| 260 | KEAP1 | 0.001811469 |
| 261 | PDGFD | 0.001856408 |
| 262 | EPAS1 | 0.001860029 |
| 263 | HPSE | 0.001882885 |
| 264 | GLI1 | 0.001884392 |
| 265 | EMX2 | 0.001909859 |
| 266 | PEPD | 0.001970188 |
| 267 | MTA1 | 0.00198428 |
| 268 | DKK1 | 0.002011592 |
| 269 | PPIA | 0.002024569 |
| 270 | STAR | 0.002044883 |
| 271 | SULT1E1 | 0.002054772 |
| 272 | MUC1 | 0.002090456 |
| 273 | RPL22 | 0.002108836 |
| 274 | PAWR | 0.002163074 |
| 275 | SERPINB5 | 0.002199947 |
| 276 | CDH13 | 0.002207718 |
| 277 | LLGL1 | 0.00221081 |
| 278 | UGT2B17 | 0.002244918 |
| 279 | LEP | 0.002246712 |
| 280 | GALR1 | 0.002271069 |
| 281 | LIMS1 | 0.002321461 |
| 282 | OGG1 | 0.002351482 |
| 283 | DDR1 | 0.002364765 |
| 284 | FAM19A5 | 0.002372217 |
| 285 | CCR1 | 0.002372982 |
| 286 | XPA | 0.002422559 |
| 287 | PROM1 | 0.002445564 |
| 288 | HAS1 | 0.002461704 |
| 289 | TNFSF10 | 0.002461967 |
| 290 | EPOR | 0.002467717 |
| 291 | TET2 | 0.002481464 |
| 292 | P2RX7 | 0.002495481 |
| 293 | PTTG1 | 0.002498574 |
| 294 | CD82 | 0.002501625 |
| 295 | SNCG | 0.002520887 |
| 296 | STMN1 | 0.002580007 |
| 297 | TERF2 | 0.002629906 |
| 298 | KIAA1324 | 0.002643334 |
| 299 | TGFBR3 | 0.002759624 |
| 300 | VEGFC | 0.002785333 |
| 301 | CLDN3 | 0.002855443 |
| 302 | TYMP | 0.003159212 |
| 303 | IGFBP1 | 0.003204088 |
| 304 | GHRL | 0.003208628 |
| 305 | PAEP | 0.003267532 |
| 306 | CYR61 | 0.0033041 |
| 307 | MGMT | 0.003386049 |
| 308 | UBE2C | 0.003398443 |
| 309 | GDF15 | 0.003427872 |
| 310 | GH1 | 0.003456286 |
| 311 | WNT10B | 0.003477618 |
| 312 | TES | 0.003503064 |
| 313 | SPOP | 0.003516838 |
| 314 | CBR1 | 0.003575208 |
| 315 | NAPSA | 0.003582655 |
| 316 | BDKRB1 | 0.003599761 |
| 317 | TIMP3 | 0.003622598 |
| 318 | CACNA2D3 | 0.003751268 |
| 319 | GGT1 | 0.003796787 |
| 320 | RCAN1 | 0.003842276 |
| 321 | HOXB13 | 0.003892798 |
| 322 | CCL20 | 0.003907061 |
| 323 | DUSP6 | 0.003913142 |
| 324 | PTP4A3 | 0.003929491 |
| 325 | ACKR3 | 0.003931191 |
| 326 | NOD2 | 0.003936584 |
| 327 | SPINT2 | 0.004012209 |
| 328 | MDK | 0.004065731 |
| 329 | KRT5 | 0.004236141 |
| 330 | ETV5 | 0.004248572 |
| 331 | ADIPOR2 | 0.004373403 |
| 332 | SCGB2A2 | 0.004394625 |
| 333 | CEACAM5 | 0.004572527 |
| 334 | DROSHA | 0.004586027 |
| 335 | TMEM140 | 0.004604414 |
| 336 | KISS1R | 0.004624188 |
| 337 | TNFSF12 | 0.004662626 |
| 338 | VCAN | 0.004713355 |
| 339 | NANOG | 0.004831378 |
| 340 | ERCC5 | 0.004904484 |
| 341 | INHA | 0.00492352 |
| 342 | STS | 0.005052201 |
| 343 | APBA2 | 0.005112632 |
| 344 | TNFRSF12A | 0.005333071 |
| 345 | MT1E | 0.005382222 |
| 346 | LAMC2 | 0.00541487 |
| 347 | MSI1 | 0.005414913 |
| 348 | KLF9 | 0.005504269 |
| 349 | NKX2-1 | 0.00556274 |
| 350 | TDGF1 | 0.005701066 |
| 351 | ADRB3 | 0.0058889 |
| 352 | SFPQ | 0.006042443 |
| 353 | TFF3 | 0.006123103 |
| 354 | HTRA1 | 0.006132181 |
| 355 | PAK3 | 0.006205531 |
| 356 | OVGP1 | 0.006218604 |
| 357 | MT-CO1 | 0.006383324 |
| 358 | SLC22A16 | 0.006396503 |
| 359 | SLC29A2 | 0.006486633 |
| 360 | SERPINB2 | 0.006494274 |
| 361 | MC4R | 0.006538752 |
| 362 | INHBC | 0.006646082 |
| 363 | SLC2A3 | 0.007365691 |
| 364 | IGFBP7 | 0.007426941 |
| 365 | TLR3 | 0.007707562 |
| 366 | GJB2 | 0.007867971 |
| 367 | ARTN | 0.008222932 |
| 368 | RCAN2 | 0.008347087 |
| 369 | LINGO2 | 0.00859135 |
| 370 | CXCL11 | 0.008618392 |
| 371 | COL17A1 | 0.00893523 |
| 372 | KRT7 | 0.008990736 |
| 373 | MST1 | 0.009181593 |
| 374 | IL11RA | 0.009335082 |
| 375 | HMMR | 0.010242032 |
| 376 | MT-ND1 | 0.010310611 |
| 377 | IL11 | 0.011392616 |
| 378 | IGF2BP3 | 0.011533568 |
| 379 | LAPTM4B | 0.011733782 |
| 380 | FOLH1 | 0.012141992 |
| 381 | MUC16 | 0.012154892 |
| 382 | TNFAIP8 | 0.013933002 |
| 383 | PTGFR | 0.014641591 |
| 384 | DLD | 0.014827735 |
| 385 | NOD1 | 0.015342271 |
| 386 | PTGDR | 0.017677767 |
| 387 | CD24 | 0.018077221 |
| 388 | GLO1 | 0.020543065 |
| 389 | NUP88 | 0.020724767 |
| 390 | RNASET2 | 0.021492724 |
| 391 | KLK4 | 0.021917686 |
| 392 | HAS3 | 0.022002222 |
| 393 | MT-ND3 | 0.024782119 |
| 394 | HCRTR2 | 0.027191368 |
| 395 | GCNT1 | 0.027387776 |
| 396 | INHBE | 0.031308661 |
| 397 | TRA2B | 0.046227831 |
| 398 | MUC5B | 0.049099375 |
