# Supplementary material for: ECGene: A Literature‐Based Knowledgebase of Endometrial Cancer Genes
Source: Hum Mutat. 2016 Jan 13;37(4):337–43. doi: 10.1002/humu.22950 (PMC5066700; doi:10.1002/humu.22950)
Supplement: Supplementary file 5 — Supp. Table S4. The biological pathways enriched for EC‐implicated genes. [file HUMU-37-337-s006.docx]

| **Supp. Table S4. The biological pathways enriched for EC-implicated genes.** | | | |
| --- | --- | --- | --- |
|  |  |  |  |
| **PathwasName** | **Database** | **P-Value** | **Corrected P-Value** |
| Signaling by SCF-KIT | Reactome | 1.11E-07 | 2.90E-05 |
| Pathways in cancer | KEGG PATHWAY | 2.99E-07 | 5.20E-05 |
| Signaling by ERBB4 | Reactome | 3.18E-07 | 5.28E-05 |
| p53 pathway feedback loops 2 | PANTHER | 4.22E-07 | 6.56E-05 |
| Signaling by FGFR in disease | Reactome | 8.34E-07 | 0.00010511 |
| NGF signalling via TRKA from the plasma membrane | Reactome | 2.44E-06 | 0.000217251 |
| @@Signaling by FGFR | Reactome | 2.70E-06 | 0.000226757 |
| Downstream signal transduction | Reactome | 2.95E-06 | 0.000239281 |
| Downstream signaling of activated FGFR | Reactome | 3.89E-06 | 0.000295983 |
| Fc epsilon receptor (FCERI) signaling | Reactome | 4.26E-06 | 0.000313523 |
| mTOR signaling pathway | KEGG PATHWAY | 5.28E-06 | 0.000354164 |
| Proteoglycans in cancer | KEGG PATHWAY | 5.72E-06 | 0.000360682 |
| Signalling by NGF | Reactome | 5.81E-06 | 0.000360682 |
| DAP12 signaling | Reactome | 1.38E-05 | 0.000722962 |
| Signaling by ERBB2 | Reactome | 1.38E-05 | 0.000722962 |
| IRS-related events triggered by IGF1R | Reactome | 2.56E-05 | 0.001067743 |
| SHC-related events triggered by IGF1R | Reactome | 2.59E-05 | 0.001067743 |
| Signaling by PDGF | Reactome | 2.62E-05 | 0.001068279 |
| p53 pathway | PANTHER | 2.72E-05 | 0.001093028 |
| ErbB signaling pathway | KEGG PATHWAY | 2.82E-05 | 0.001127657 |
| HIF-1 signaling pathway | KEGG PATHWAY | 3.32E-05 | 0.001282714 |
| Signaling by Type 1 Insulin-like Growth Factor 1 Receptor (IGF1R) | Reactome | 3.53E-05 | 0.001328553 |
| IGF1R signaling cascade | Reactome | 3.53E-05 | 0.001328553 |
| DAP12 interactions | Reactome | 3.94E-05 | 0.001455845 |
| Signaling by Overexpressed Wild-Type EGFR in Cancer | Reactome | 4.23E-05 | 0.001508443 |
| Signaling by EGFR | Reactome | 4.23E-05 | 0.001508443 |
| Constitutive PI3K/AKT Signaling in Cancer | Reactome | 4.79E-05 | 0.001634532 |
| Signaling by EGFR in Cancer | Reactome | 4.86E-05 | 0.001634532 |
| Signaling by EGFRvIII in Cancer | Reactome | 4.86E-05 | 0.001634532 |
| Signaling by Ligand-Responsive EGFR Variants in Cancer | Reactome | 4.86E-05 | 0.001634532 |
| Thyroid hormone signaling pathway | KEGG PATHWAY | 5.05E-05 | 0.001677806 |
| Central carbon metabolism in cancer | KEGG PATHWAY | 5.94E-05 | 0.001884259 |
| GRB2 events in ERBB2 signaling | Reactome | 6.17E-05 | 0.001918121 |
| p53 signaling pathway | KEGG PATHWAY | 6.87E-05 | 0.002024227 |
| Cellular responses to stress | Reactome | 7.16E-05 | 0.002092951 |
| SHC1 events in ERBB2 signaling | Reactome | 7.51E-05 | 0.002155131 |
| GRB2 events in EGFR signaling | Reactome | 8.38E-05 | 0.002346511 |
| FoxO signaling pathway | KEGG PATHWAY | 8.73E-05 | 0.002390805 |
| FCERI mediated MAPK activation | Reactome | 0.000106027 | 0.002712331 |
| SHC1 events in EGFR signaling | Reactome | 0.000106148 | 0.002712331 |
| VEGFR2 mediated cell proliferation | Reactome | 0.000130097 | 0.003148182 |
| Signalling to p38 via RIT and RIN | Reactome | 0.000132973 | 0.003207152 |
| Signaling by VEGF | Reactome | 0.00013796 | 0.003292807 |
| AKT phosphorylates targets in the cytosol | Reactome | 0.00015802 | 0.003666063 |
| PI-3K cascade | Reactome | 0.000163297 | 0.003694658 |
| PIP3 activates AKT signaling | Reactome | 0.000163297 | 0.003694658 |
| PI3K events in ERBB2 signaling | Reactome | 0.000163297 | 0.003694658 |
| PI3K events in ERBB4 signaling | Reactome | 0.000163297 | 0.003694658 |
| PI3K/AKT Signaling in Cancer | Reactome | 0.000163297 | 0.003694658 |
| ARMS-mediated activation | Reactome | 0.000164898 | 0.003719363 |
| Cellular Senescence | Reactome | 0.000184315 | 0.004026263 |
| Signaling by Leptin | Reactome | 0.000202585 | 0.004291288 |
| GAB1 signalosome | Reactome | 0.000214889 | 0.004474107 |
| PI3K/AKT activation | Reactome | 0.000214889 | 0.004474107 |
| VEGFA-VEGFR2 Pathway | Reactome | 0.000241501 | 0.004861948 |
| Frs2-mediated activation | Reactome | 0.000246745 | 0.004940308 |
| IRS-mediated signalling | Reactome | 0.000311833 | 0.005903817 |
| Role of LAT2/NTAL/LAB on calcium mobilization | Reactome | 0.000331362 | 0.00622517 |
| EGFR Transactivation by Gastrin | Reactome | 0.000344834 | 0.006445137 |
| RAF/MAP kinase cascade | Reactome | 0.000347868 | 0.006485246 |
| Prolonged ERK activation events | Reactome | 0.000357543 | 0.006598291 |
| Intrinsic Pathway for Apoptosis | Reactome | 0.000388799 | 0.007033033 |
| IRS-related events | Reactome | 0.000410835 | 0.007340781 |
| Acute myeloid leukemia | KEGG PATHWAY | 0.000515233 | 0.008592523 |
| Renal cell carcinoma | KEGG PATHWAY | 0.000532909 | 0.008771391 |
| VEGF signaling pathway | PANTHER | 0.000544252 | 0.008831678 |
| Insulin receptor signalling cascade | Reactome | 0.000582229 | 0.009150387 |
| Activation of the AP-1 family of transcription factors | Reactome | 0.000664217 | 0.010049899 |
| SOS-mediated signalling | Reactome | 0.000673626 | 0.010171202 |
| SHC-mediated signalling | Reactome | 0.000821082 | 0.011854454 |
| ERK activation | Reactome | 0.000884019 | 0.012517861 |
| Insulin/IGF pathway-protein kinase B signaling cascade | PANTHER | 0.000942297 | 0.013176272 |
| MAP kinase activation in TLR cascade | Reactome | 0.000947341 | 0.013212148 |
| Interleukin-2 signaling | Reactome | 0.000953293 | 0.013244617 |
| NOTCH1 Intracellular Domain Regulates Transcription | Reactome | 0.000953293 | 0.013244617 |
| Angiogenesis | PANTHER | 0.001052844 | 0.014117772 |
| Prolactin signaling pathway | KEGG PATHWAY | 0.001084854 | 0.014414755 |
| Cell cycle | KEGG PATHWAY | 0.001324784 | 0.016217973 |
| SHC-related events | Reactome | 0.001408441 | 0.016901293 |
| MicroRNAs in cancer | KEGG PATHWAY | 0.001446049 | 0.017267526 |
| Toll Like Receptor 3 (TLR3) Cascade | Reactome | 0.001826424 | 0.020725937 |
| TRIF-mediated TLR3/TLR4 signaling | Reactome | 0.001826424 | 0.020725937 |
| MyD88-independent cascade | Reactome | 0.001826424 | 0.020725937 |
| Constitutive Signaling by EGFRvIII | Reactome | 0.001842805 | 0.020852486 |
| telomeres telomerase cellular aging and immortality | BioCarta | 0.001920662 | 0.021364073 |
| SHC1 events in ERBB4 signaling | Reactome | 0.001942244 | 0.021561073 |
| Cellular response to heat stress | Reactome | 0.002043401 | 0.022221981 |
| Nuclear Receptor transcription pathway | Reactome | 0.002043401 | 0.022221981 |
| Hypoxia response via HIF activation | PANTHER | 0.002192813 | 0.023394274 |
| Progesterone-mediated oocyte maturation | KEGG PATHWAY | 0.002270785 | 0.023985291 |
| Mismatch repair (MMR) directed by MSH2:MSH6 (MutSalpha) | Reactome | 0.002278462 | 0.023985291 |
| @@VEGF signaling pathway | KEGG PATHWAY | 0.002578514 | 0.026428866 |
| Mismatch Repair | Reactome | 0.00278012 | 0.027492716 |
| binding of TCF/LEF:CTNNB1 to target gene promoters | Reactome | 0.002856322 | 0.027864805 |
| Signalling to ERKs | Reactome | 0.002877771 | 0.028003664 |
| role of erbb2 in signal transduction and oncology | BioCarta | 0.003247972 | 0.030161602 |
| Signaling by Insulin receptor | Reactome | 0.003272512 | 0.03019636 |
| ERK2 activation | Reactome | 0.003839108 | 0.0343826 |
| Oncogene Induced Senescence | Reactome | 0.003904077 | 0.034793897 |
| MAPK targets/ Nuclear events mediated by MAP kinases | Reactome | 0.003904077 | 0.034793897 |
| MyD88:Mal cascade initiated on plasma membrane | Reactome | 0.003939594 | 0.03501149 |
| Toll Like Receptor TLR6:TLR2 Cascade | Reactome | 0.003939594 | 0.03501149 |
| Constitutive Signaling by Ligand-Responsive EGFR Cancer Variants | Reactome | 0.003999851 | 0.035257981 |
| Activated TLR4 signalling | Reactome | 0.004154313 | 0.035928665 |
| Signalling to RAS | Reactome | 0.004422001 | 0.037796475 |
| Toll Like Receptor TLR1:TLR2 Cascade | Reactome | 0.004770614 | 0.039889757 |
| Toll Like Receptor 2 (TLR2) Cascade | Reactome | 0.004770614 | 0.039889757 |
| Regulation of gene expression by Hypoxia-inducible Factor | Reactome | 0.005003866 | 0.041087924 |
| ERK1 activation | Reactome | 0.005003866 | 0.041087924 |
| cell cycle: g1/s check point | BioCarta | 0.005145492 | 0.042061805 |
| Downstream signaling events of B Cell Receptor (BCR) | Reactome | 0.005217746 | 0.042179296 |
| TNF signaling pathway | KEGG PATHWAY | 0.005409844 | 0.043397518 |
| Regulation of HSF1-mediated heat shock response | Reactome | 0.005537418 | 0.04426928 |
| deactivation of the beta-catenin transactivating complex | Reactome | 0.005601295 | 0.044542181 |
| MAP kinase cascade | BioCyc | 0.006272429 | 0.048251482 |
| Interleukin-6 signaling | Reactome | 0.006359191 | 0.048713799 |
| CD28 dependent PI3K/Akt signaling | Reactome | 0.006435768 | 0.049197273 |
