# Supplementary material for: ECGene: A Literature‐Based Knowledgebase of Endometrial Cancer Genes
Source: Hum Mutat. 2016 Jan 13;37(4):337–43. doi: 10.1002/humu.22950 (PMC5066700; doi:10.1002/humu.22950)
Supplement: Supplementary file 6 — Supp. Table S5. The pan‐cancer mutational frequency for the top 99 ranked EC‐implicated genes. [file HUMU-37-337-s007.docx]

**Supp. Table S5: The pan-cancer mutational frequency for the top 99 ranked EC-implicated genes.**

| **STUDY_ABBREVIATION** | **STUDY_NAME** | **NUM_OF_CASES_ALTERED** | **PERCENT_CASES_ALTERED** |
| --- | --- | --- | --- |
| Breast (BCCRC Xenograft) | Breast cancer patient xenografts (British Columbia, Nature 2014) | 29 | 100% |
| Colorectal (MSKCC) | Colorectal Adenocarcinoma Triplets (MSKCC, Genome Biology 2014) | 138 | 100% |
| NCI-60 | NCI-60 Cell Lines (NCI, Cancer Res. 2012) | 53 | 100% |
| Lung squ (TCGA) | Lung Squamous Cell Carcinoma (TCGA, Provisional) | 176 | 98.90% |
| Uterine (TCGA) | Uterine Corpus Endometrial Carcinoma (TCGA, Provisional) | 239 | 98.80% |
| Uterine (TCGA pub) | Uterine Corpus Endometrioid Carcinoma (TCGA, Nature 2013) | 237 | 98.80% |
| Colorectal (TCGA pub) | Colorectal Adenocarcinoma (TCGA, Nature 2012) | 209 | 98.60% |
| Bladder (TCGA pub) | Bladder Urothelial Carcinoma (TCGA, Nature 2014) | 125 | 98.40% |
| Bladder (TCGA) | Bladder Urothelial Carcinoma (TCGA, Provisional) | 125 | 98.40% |
| Lung adeno (TCGA) | Lung Adenocarcinoma (TCGA, Provisional) | 169 | 98.30% |
| Melanoma (TCGA) | Skin Cutaneous Melanoma (TCGA, Provisional) | 273 | 98.20% |
| CCLE | Cancer Cell Line Encyclopedia (Novartis/Broad, Nature 2012) | 862 | 97.80% |
| Lung adeno (TCGA pub) | Lung Adenocarcinoma (TCGA, Nature 2014) | 223 | 97% |
| Stomach (TCGA pub) | Stomach Adenocarcinoma (TCGA, Nature 2014) | 278 | 96.90% |
| Colorectal (TCGA) | Colorectal Adenocarcinoma (TCGA, Provisional) | 213 | 96.80% |
| Lung SC (CLCGP) | Small Cell Lung Cancer (CLCGP, Nature Genetics 2012) | 28 | 96.60% |
| GBM (TCGA) | Glioblastoma Multiforme (TCGA, Provisional) | 263 | 96.30% |
| GBM (TCGA 2013) | Glioblastoma (TCGA, Cell 2013) | 270 | 96.10% |
| Prostate (SU2C) | Metastatic Prostate Cancer, SU2C/PCF Dream Team (Robinson et al., Cell 2015) | 144 | 96% |
| Pancreas (ICGC) | Pancreatic Adenocarcinoma (ICGC, Nature 2012) | 95 | 96% |
| Stomach (TCGA) | Stomach Adenocarcinoma (TCGA, Provisional) | 211 | 95.90% |
| Lung squ (TCGA pub) | Lung Squamous Cell Carcinoma (TCGA, Nature 2012) | 169 | 94.90% |
| Uterine CS (TCGA) | Uterine Carcinosarcoma (TCGA, Provisional) | 53 | 94.60% |
| Ovarian (TCGA) | Ovarian Serous Cystadenocarcinoma (TCGA, Provisional) | 294 | 94.50% |
| Prostate (MICH) | Prostate Adenocarcinoma, Metastatic (Michigan, Nature 2012) | 57 | 93.40% |
| MPNST (MSKCC) | Malignant Peripheral Nerve Sheath Tumor (MSKCC, Nature Genetics 2014) | 14 | 93.30% |
| Pancreas (TCGA) | Pancreatic Adenocarcinoma (TCGA, Provisional) | 84 | 93.30% |
| Head & neck (TCGA pub) | Head and Neck Squamous Cell Carcinoma (TCGA, in revision) | 260 | 93.20% |
| Colorectal (Genentech) | Colorectal Adenocarcinoma (Genentech, Nature 2012) | 67 | 93.10% |
| Head & neck (TCGA) | Head and Neck Squamous Cell Carcinoma (TCGA, Provisional) | 281 | 93% |
| Melanoma(broad/dfarber) | Melanoma (Broad/Dana Farber, Nature 2012) | 23 | 92% |
| Esophagus (TCGA) | Esophageal Carcinoma (TCGA, Provisional) | 169 | 91.80% |
| Melanoma (Broad) | Skin Cutaneous Melanoma (Broad, Cell 2012) | 111 | 91.70% |
| Breast (TCGA pub) | Breast Invasive Carcinoma (TCGA, Nature 2012) | 442 | 91.70% |
| GBM (TCGA 2008) | Glioblastoma (TCGA, Nature 2008) | 83 | 91.20% |
| Liver (TCGA) | Liver Hepatocellular Carcinoma (TCGA, Provisional) | 176 | 91.20% |
| Breast (TCGA) | Breast Invasive Carcinoma (TCGA, Provisional) | 872 | 90.60% |
| Cervical (TCGA) | Cervical Squamous Cell Carcinoma and Endocervical Adenocarcinoma (TCGA, Provisional) | 171 | 89.50% |
| Ovarian (TCGA pub) | Ovarian Serous Cystadenocarcinoma (TCGA, Nature 2011) | 282 | 89.20% |
| Lung adeno (Broad) | Lung Adenocarcinoma (Broad, Cell 2012) | 162 | 89% |
| Lung SC (JHU) | Small Cell Lung Cancer (Johns Hopkins, Nature Genetics 2012) | 37 | 88.10% |
| Sarcoma (TCGA) | Sarcoma (TCGA, Provisional) | 212 | 82.50% |
| Lung adeno (TSP) | Lung Adenocarcinoma (TSP, Nature 2008) | 134 | 82.20% |
| Bladder (MSKCC 2014) | Bladder Cancer (MSKCC, Eur Urol 2014) | 88 | 80.70% |
| Bladder (MSKCC 2012) | Bladder Cancer (MSKCC, JCO 2013) | 74 | 76.30% |
| Glioma (TCGA) | Brain Lower Grade Glioma (TCGA, Provisional) | 218 | 76.20% |
| ACC (TCGA) | Adrenocortical Carcinoma (TCGA, Provisional) | 67 | 76.10% |
| Prostate (TCGA) | Prostate Adenocarcinoma (TCGA, Provisional) | 187 | 72.50% |
| Thyroid (TCGA pub) | Papillary Thyroid Carcinoma (TCGA, Cell 2014) | 284 | 71.20% |
| Thyroid (TCGA) | Thyroid Carcinoma (TCGA, Provisional) | 276 | 69.20% |
| Prostate (TCGA 2015) | Prostate Adenocarcinoma (TCGA, in preparation) | 230 | 69.10% |
| Head & neck (Broad) | Head and Neck Squamous Cell Carcinoma (Broad, Science 2011) | 51 | 68.90% |
| Esophagus (Broad) | Esophageal Adenocarcinoma (Broad, Nature Genetics 2013) | 100 | 68.50% |
| Stomach (UHK) | Stomach Adenocarcinoma (UHK, Nature Genetics 2011) | 15 | 68.20% |
| Bladder (BGI) | Bladder Urothelial Carcinoma (BGI, Nature Genetics 2013) | 66 | 66.70% |
| DLBC (TCGA) | Lymphoid Neoplasm Diffuse Large B-cell Lymphoma (TCGA, Provisional) | 32 | 66.70% |
| Prostate (Broad/Cornell 2013) | Prostate Adenocarcinoma (Broad/Cornell, Cell 2013) | 36 | 64.30% |
| Stomach (Pfizer UHK) | Stomach Adenocarcinoma (Pfizer and UHK, Nature Genetics 2014) | 64 | 64% |
| ACyC (MSKCC) | Adenoid Cystic Carcinoma (MSKCC, Nature Genetics 2013) | 38 | 63.30% |
| Melanoma (Yale) | Skin Cutaneous Melanoma (Yale, Nature Genetics 2012) | 57 | 62.60% |
| Cholangiocarcinoma (NUS) | Cholangiocarcinoma (National University of Singapore,Nature Genetics 2012) | 5 | 62.50% |
| Liver (AMC) | Liver Hepatocellular Carcinoma (AMC, Hepatology 2014) | 142 | 61.50% |
| Breast (Broad) | Breast Invasive Carcinoma (Broad, Nature 2012) | 61 | 59.20% |
| Breast (Sanger) | Breast Invasive Carcinoma (Sanger, Nature 2012) | 59 | 59% |
| Sarcoma (MSKCC) | Sarcoma (MSKCC/Broad, Nature Genetics 2010) | 122 | 58.90% |
| pRCC (TCGA) | Kidney Renal Papillary Cell Carcinoma (TCGA, Provisional) | 94 | 58.40% |
| ccRCC (TCGA) | Kidney Renal Clear Cell Carcinoma (TCGA, Provisional) | 233 | 56.10% |
| chRCC (TCGA) | Kidney Chromophobe (TCGA, Provisional) | 37 | 56.10% |
| Stomach (UTokyo) | Stomach Adenocarcinoma (U Tokyo, Nature Genetics 2014) | 16 | 53.30% |
| Breast (BCCRC) | Breast Invasive Carcinoma (British Columbia, Nature 2012) | 31 | 47.70% |
| Prostate (MSKCC 2010) | Prostate Adenocarcinoma (MSKCC, Cancer Cell 2010) | 49 | 47.60% |
| MM (Broad) | Multiple Myeloma (Broad, Cancer Cell 2014) | 95 | 46.30% |
| ccRCC (TCGA pub) | Kidney Renal Clear Cell Carcinoma (TCGA, Nature 2013) | 193 | 46.20% |
| Prostate (Broad/Cornell 2012) | Prostate Adenocarcinoma (Broad/Cornell, Nature Genetics 2012) | 50 | 45.90% |
| Head & neck (JHU) | Head and Neck Squamous Cell Carcinoma (Johns Hopkins, Science 2011) | 14 | 43.80% |
| Esophagus sq (ICGC) | Esophageal Squamous Cell Carcinoma (ICGC, Nature 2014) | 38 | 43.20% |
| Cholangiocarcinoma (JHU) | Intrahepatic Cholangiocarcinoma (Johns Hopkins University, Nature Genetics 2013) | 17 | 42.50% |
| chRCC (TCGA) | Kidney Chromophobe (TCGA, Cancer Cell 2014) | 24 | 36.90% |
| PCPG (TCGA) | Pheochromocytoma and Paraganglioma (TCGA, Provisional) | 58 | 36% |
| Liver (RIKEN) | Liver Hepatocellular Carcinoma (RIKEN, Nature Genetics 2012) | 7 | 33.30% |
| Prostate Organoids | Prostate Adenocarcinoma Organoids (MSKCC, Cell 2014) | 4 | 33.30% |
| Cholangiocarcinoma (NCCS) | Cholangiocarcinoma (National Cancer Centre of Singapore, Nature Genetics 2013) | 4 | 26.70% |
| NPC (Singapore) | Nasopharyngeal Carcinoma (Singapore, Nature Genetics 2014) | 14 | 25% |
| AML (TCGA) | Acute Myeloid Leukemia (TCGA, Provisional) | 45 | 23.90% |
| MBL (ICGC) | Medulloblastoma (ICGC, Nature 2012) | 27 | 23.70% |
| AML (TCGA pub) | Acute Myeloid Leukemia (TCGA, NEJM 2013) | 45 | 23.60% |
| MBL (Broad) | Medulloblastoma (Broad, Nature 2012) | 20 | 21.70% |
| MBL (PCGP) | Medulloblastoma (PCGP, Nature 2012) | 7 | 18.90% |
| ccRCC (BGI) | Kidney Renal Clear Cell Carcinoma (BGI, Nature Genetics 2012) | 15 | 18.50% |
| Prostate (MSKCC 2014) | Prostate Adenocarcinoma CNA study (MSKCC, PNAS 2014) | 6 | 5.80% |
| Ovary SC (MSKCC) | Small Cell Carcinoma of the Ovary (MSKCC, Nature Genetics 2014) | 0 | 0% |
