# Supplementary material for: ECGene: A Literature‐Based Knowledgebase of Endometrial Cancer Genes
Source: Hum Mutat. 2016 Jan 13;37(4):337–43. doi: 10.1002/humu.22950 (PMC5066700; doi:10.1002/humu.22950)
Supplement: Supplementary file 7 — Supp. Table S6. The mutational frequency for the top 100 ranked EC‐implicated genes in TCGA endometrial cancer samples. [file HUMU-37-337-s008.docx]

| **Supp. Table S6. The mutational frequency for the top 100 ranked EC-implicated genes in TCGA endometrial cancer samples.** | | |
| --- | --- | --- |
|  |  |  |
| **GENE_SYMBOL** | **NUM_CASES_ALTERED** | **PERCENT_CASES_ALTERED** |
| PTEN | 174 | 47% |
| PIK3CA | 163 | 44% |
| PIK3R1 | 99 | 27% |
| CTNNB1 | 91 | 24% |
| TP53 | 85 | 23% |
| KRAS | 74 | 20% |
| PTK2 | 69 | 18% |
| MYC | 62 | 17% |
| FGFR2 | 55 | 15% |
| NR5A2 | 55 | 15% |
| ERBB2 | 53 | 14% |
| ATM | 51 | 14% |
| SCRIB | 52 | 14% |
| ESRRG | 52 | 14% |
| ATR | 47 | 13% |
| MSH6 | 49 | 13% |
| PTGS2 | 48 | 13% |
| MTOR | 45 | 12% |
| CASP8 | 46 | 12% |
| NOTCH1 | 44 | 12% |
| PGR | 43 | 12% |
| IGF1R | 45 | 12% |
| MSH2 | 44 | 12% |
| PRKCA | 46 | 12% |
| APC | 41 | 11% |
| ESR1 | 40 | 11% |
| CDH1 | 41 | 11% |
| MAP2K4 | 42 | 11% |
| CCNE1 | 40 | 11% |
| CCND1 | 39 | 10% |
| MLH3 | 37 | 10% |
| TP63 | 38 | 10% |
| EP300 | 38 | 10% |
| EGFR | 37 | 10% |
| BRAF | 39 | 10% |
| TERF1 | 36 | 10% |
| RB1 | 34 | 9% |
| AKT1 | 34 | 9% |
| KDR | 35 | 9% |
| TSC2 | 34 | 9% |
| CYP19A1 | 32 | 9% |
| AR | 35 | 9% |
| MET | 34 | 9% |
| CHEK2 | 33 | 9% |
| KDM4A | 32 | 9% |
| MAPK1 | 32 | 9% |
| TERT | 32 | 9% |
| SMAD7 | 32 | 9% |
| PIK3C2A | 30 | 8% |
| STK11 | 28 | 8% |
| MAPK8 | 29 | 8% |
| CDKN1B | 30 | 8% |
| BAX | 31 | 8% |
| PMS2 | 30 | 8% |
| HDAC1 | 30 | 8% |
| MAPK3 | 28 | 8% |
| CASP3 | 30 | 8% |
| CDKN1A | 29 | 8% |
| CDC25B | 28 | 8% |
| PAK4 | 29 | 8% |
| GPER1 | 27 | 7% |
| PPARG | 27 | 7% |
| RPS6KA2 | 27 | 7% |
| NCOR1 | 27 | 7% |
| CDKN2A | 26 | 7% |
| STAT3 | 27 | 7% |
| RAD51 | 26 | 7% |
| BRCA1 | 27 | 7% |
| COMT | 23 | 6% |
| SPA17 | 22 | 6% |
| MDM2 | 24 | 6% |
| MMP2 | 24 | 6% |
| TCF7L2 | 23 | 6% |
| HIF1A | 21 | 6% |
| CTSV | 21 | 6% |
| CYP1A1 | 23 | 6% |
| JAK1 | 22 | 6% |
| BUB1 | 23 | 6% |
| TLR4 | 22 | 6% |
| NME1 | 22 | 6% |
| APOE | 23 | 6% |
| VEGFA | 23 | 6% |
| BCL2 | 21 | 6% |
| NTRK2 | 20 | 5% |
| MMP9 | 20 | 5% |
| HDAC2 | 19 | 5% |
| FOS | 17 | 5% |
| TNF | 17 | 5% |
| IGF1 | 16 | 4% |
| TGFB1 | 16 | 4% |
| RCAN3 | 16 | 4% |
| ESR2 | 15 | 4% |
| IL6 | 15 | 4% |
| RUNX3 | 15 | 4% |
| MLH1 | 15 | 4% |
| TARP | 13 | 3% |
| CYP17A1 | 10 | 3% |
| CYP1B1 | 10 | 3% |
| MUC8 | 0 | 0% |
