# Supplementary material for: ECGene: A Literature‐Based Knowledgebase of Endometrial Cancer Genes
Source: Hum Mutat. 2016 Jan 13;37(4):337–43. doi: 10.1002/humu.22950 (PMC5066700; doi:10.1002/humu.22950)
Supplement: Supplementary file 8 — Supp. Table S7. The network topological properties for the reconstructed EC‐implicated interactome. [file HUMU-37-337-s009.docx]

| **Supp. Table S7. The network topological properties for the reconstructed EC-implicated interactome.** | |
| --- | --- |
| **GeneSymbol** | **Degree** |
| TP53 | 47 |
| MYC | 37 |
| CTNNB1 | 33 |
| AKT1 | 23 |
| NFKB1 | 23 |
| ESR1 | 22 |
| HDAC1 | 22 |
| AR | 22 |
| PIK3CA | 21 |
| FOS | 21 |
| PIK3R1 | 20 |
| JAK2 | 19 |
| STAT3 | 19 |
| MTOR | 17 |
| EP300 | 17 |
| RPA1 | 17 |
| RB1 | 16 |
| CCND1 | 16 |
| HIF1A | 16 |
| ITGAV | 16 |

| **ShortPathStep** | **Numer of pairs** |
| --- | --- |
| 1 | 1520 |
| 2 | 10520 |
| 3 | 24504 |
| 4 | 24000 |
| 5 | 12508 |
| 6 | 5130 |
| 7 | 1306 |
| 8 | 288 |
| 9 | 28 |
| 10 | 2 |
