# Supplementary material for: ECGene: A Literature‐Based Knowledgebase of Endometrial Cancer Genes
Source: Hum Mutat. 2016 Jan 13;37(4):337–43. doi: 10.1002/humu.22950 (PMC5066700; doi:10.1002/humu.22950)
Supplement: Supplementary file 9 — Supp. Table S8. The enriched functional terms of 11 hub genes from the EC‐implicated interactome. [file HUMU-37-337-s010.docx]

| **Supp. Table S8. The enriched functional terms of 11 hub genes from the EC-implicated interactome.** | |
| --- | --- |
|  |  |
| **Name** | **q-value FDR B&H** |
| Pathways in cancer | 5.34E-12 |
| cellular response to endogenous stimulus | 1.48E-11 |
| enzyme linked receptor protein signaling pathway | 1.48E-11 |
| Colorectal cancer | 1.94E-11 |
| Chronic myeloid leukemia | 3.82E-11 |
| TGF-beta Receptor Signaling Pathway | 3.82E-11 |
| Integrated Breast Cancer Pathway | 3.82E-11 |
| Prostate cancer | 8.98E-11 |
| Integrated Pancreatic Cancer Pathway | 1.70E-10 |
| p53 pathway feedback loops 2 | 1.70E-10 |
| Nongenotropic Androgen signaling | 2.83E-10 |
| Endometrial cancer | 3.39E-10 |
| response to endogenous stimulus | 3.60E-10 |
| Estrogen signaling pathway | 4.18E-10 |
| positive regulation of transcription from RNA polymerase II promoter | 4.71E-10 |
| Inhibition of Cellular Proliferation by Gleevec | 8.30E-10 |
| HTLV-I infection | 1.07E-09 |
| Regulation of Telomerase | 1.07E-09 |
| Hepatitis B | 1.24E-09 |
| Inactivation of Gsk3 by AKT causes accumulation of b-catenin in Alveolar Macrophages | 1.49E-09 |
| Prolactin signaling pathway | 1.52E-09 |
| Prolactin Signaling Pathway | 1.85E-09 |
| cellular response to growth factor stimulus | 1.95E-09 |
| Regulation of nuclear SMAD2/3 signaling | 2.23E-09 |
| response to growth factor | 2.03E-09 |
| cellular response to organic substance | 2.61E-09 |
| Small cell lung cancer | 3.86E-09 |
| transcription factor binding | 5.20E-09 |
| transcription regulatory region DNA binding | 5.20E-09 |
| regulatory region DNA binding | 5.20E-09 |
| regulatory region nucleic acid binding | 5.20E-09 |
| positive regulation of transcription, DNA-templated | 3.89E-09 |
| positive regulation of RNA biosynthetic process | 4.81E-09 |
| positive regulation of RNA metabolic process | 5.14E-09 |
| negative regulation of cell death | 5.19E-09 |
| IL2 signaling events mediated by PI3K | 6.11E-09 |
| IL-2 Receptor Beta Chain in T cell Activation | 6.71E-09 |
| positive regulation of gene expression | 6.18E-09 |
| IL-6 Signaling Pathway | 7.96E-09 |
| Epstein-Barr virus infection | 7.96E-09 |
| regulation of apoptotic process | 7.88E-09 |
| regulation of programmed cell death | 8.22E-09 |
| cellular response to hormone stimulus | 9.46E-09 |
| positive regulation of macromolecule biosynthetic process | 9.46E-09 |
| positive regulation of developmental process | 9.46E-09 |
| positive regulation of nucleobase-containing compound metabolic process | 9.46E-09 |
| IL6-mediated signaling events | 1.13E-08 |
| regulation of cell death | 1.01E-08 |
| positive regulation of nitrogen compound metabolic process | 1.09E-08 |
| Prostate Cancer | 1.27E-08 |
| transcription regulatory region sequence-specific DNA binding | 1.71E-08 |
| Proteoglycans in cancer | 1.46E-08 |
| positive regulation of cellular biosynthetic process | 1.57E-08 |
| positive regulation of biosynthetic process | 1.82E-08 |
| regulation of transcription from RNA polymerase II promoter | 2.39E-08 |
| B Cell Survival Pathway | 2.91E-08 |
| Tumor Suppressor Arf Inhibits Ribosomal Biogenesis | 3.55E-08 |
| Human Cytomegalovirus and Map Kinase Pathways | 3.55E-08 |
| Acute myeloid leukemia | 3.94E-08 |
| Fc-epsilon receptor I signaling in mast cells | 4.55E-08 |
| Oncostatin M Signaling Pathway | 6.19E-08 |
| Corticosteroids and cardioprotection | 6.33E-08 |
| TSH signaling pathway | 6.33E-08 |
| transcription from RNA polymerase II promoter | 6.07E-08 |
| apoptotic process | 6.13E-08 |
| BCR signaling pathway | 7.01E-08 |
| Pancreatic cancer | 7.01E-08 |
| transmembrane receptor protein tyrosine kinase signaling pathway | 6.14E-08 |
| Trefoil Factors Initiate Mucosal Healing | 7.03E-08 |
| programmed cell death | 6.35E-08 |
| negative regulation of apoptotic process | 6.35E-08 |
| Lung Neoplasms | 8.67E-08 |
| negative regulation of programmed cell death | 7.05E-08 |
| AKT Signaling Pathway | 8.37E-08 |
| Ras Signaling Pathway | 9.64E-08 |
| CTCF: First Multivalent Nuclear Factor | 9.64E-08 |
| B cell receptor signaling pathway | 9.64E-08 |
| cell-type specific apoptotic process | 8.70E-08 |
| regulation of developmental process | 8.70E-08 |
| T cell activation | 1.24E-07 |
| structure-specific DNA binding | 1.58E-07 |
| EGFR1 Signaling Pathway | 1.41E-07 |
| p53 pathway | 1.42E-07 |
| Plasma membrane estrogen receptor signaling | 1.42E-07 |
| Influence of Ras and Rho proteins on G1 to S Transition | 1.42E-07 |
| cellular response to abiotic stimulus | 1.34E-07 |
| RNA polymerase II regulatory region sequence-specific DNA binding | 1.96E-07 |
| RNA polymerase II regulatory region DNA binding | 1.96E-07 |
| double-stranded DNA binding | 1.96E-07 |
| Glucocorticoid receptor regulatory network | 1.64E-07 |
| Apoptosis | 1.71E-07 |
| Apoptosis | 2.00E-07 |
| Androgen receptor signaling pathway | 2.21E-07 |
| response to hormone | 2.04E-07 |
| DNA damage response (only ATM dependent) | 2.56E-07 |
| regulation of cell differentiation | 2.29E-07 |
| positive regulation of phosphorus metabolic process | 2.51E-07 |
| positive regulation of phosphate metabolic process | 2.51E-07 |
| Osteopontin-mediated events | 3.01E-07 |
| E-cadherin signaling in the nascent adherens junction | 3.36E-07 |
| regulation of multicellular organismal development | 3.12E-07 |
| Estrogen signaling pathway | 3.91E-07 |
| nucleoplasm | 5.40E-07 |
| ErbB2/ErbB3 signaling events | 4.10E-07 |
| Apoptosis signaling pathway | 4.10E-07 |
| Regulation of toll-like receptor signaling pathway | 4.10E-07 |
| Toll-like receptor signaling pathway | 4.16E-07 |
| ErbB1 downstream signaling | 4.16E-07 |
| Adrenergic Pathway | 4.16E-07 |
| Chagas disease (American trypanosomiasis) | 4.16E-07 |
| T cell receptor signaling pathway | 4.16E-07 |
| Toll-like receptor signaling pathway | 4.51E-07 |
| TSLP Signaling Pathway | 5.03E-07 |
| MicroRNAs in cardiomyocyte hypertrophy | 5.03E-07 |
| TNF signaling pathway | 5.19E-07 |
| B Cell Antigen Receptor | 5.94E-07 |
| transcription factor activity, sequence-specific DNA binding | 7.64E-07 |
| nucleic acid binding transcription factor activity | 7.64E-07 |
| positive regulation of response to stimulus | 5.93E-07 |
| IL-2 Signaling pathway | 7.16E-07 |
| apoptotic signaling pathway | 6.40E-07 |
| Neurotrophin signaling pathway | 7.67E-07 |
| TWEAK Signaling Pathway | 7.67E-07 |
| COLORECTAL CANCER; CRC | 1.04E-06 |
| Familial cancer of breast | 1.04E-06 |
| BREAST CANCER | 1.04E-06 |
| negative regulation of cell proliferation | 7.79E-07 |
| Presenilin action in Notch and Wnt signaling | 9.99E-07 |
| T Cell Receptor Signaling Pathway | 1.06E-06 |
| FGF signaling pathway | 1.06E-06 |
| Angiopoietin receptor Tie2-mediated signaling | 1.14E-06 |
| Osteoclast differentiation | 1.14E-06 |
| Hepatitis C | 1.17E-06 |
| Measles | 1.20E-06 |
| sequence-specific DNA binding | 1.50E-06 |
| negative regulation of macromolecule metabolic process | 1.15E-06 |
| negative regulation of response to stimulus | 1.17E-06 |
| regulation of apoptotic signaling pathway | 1.18E-06 |
| Neoplasm of ovary | 1.63E-06 |
| IL2-mediated signaling events | 1.51E-06 |
| regulation of glucose import | 1.36E-06 |
| Trka Receptor Signaling Pathway | 1.63E-06 |
| BDNF signaling pathway | 1.66E-06 |
| RANKL/RANK Signaling Pathway | 1.67E-06 |
| PDGFR-beta signaling pathway | 1.67E-06 |
| tissue development | 1.51E-06 |
| Non-small cell lung cancer | 1.92E-06 |
| Angiogenesis | 1.94E-06 |
| Fc-epsilon receptor signaling pathway | 1.74E-06 |
| OVARIAN CANCER | 2.38E-06 |
| Carcinoma, Hepatocellular | 2.43E-06 |
| glucose import | 1.89E-06 |
| regulation of immune system process | 1.89E-06 |
| response to mechanical stimulus | 1.97E-06 |
| negative regulation of metabolic process | 2.05E-06 |
| response to organic cyclic compound | 2.11E-06 |
| Leptin signaling pathway | 2.47E-06 |
| Atypical NF-kappaB pathway | 2.47E-06 |
| regulation of phosphorylation | 2.20E-06 |
| response to wounding | 2.22E-06 |
| response to organonitrogen compound | 2.22E-06 |
| p75(NTR)-mediated signaling | 2.59E-06 |
| Breast Neoplasms | 3.11E-06 |
| Adenocarcinoma | 3.11E-06 |
| HEPATOCELLULAR CARCINOMA | 3.11E-06 |
| Hepatocellular carcinoma | 3.11E-06 |
| RNA polymerase II transcription factor binding | 3.30E-06 |
| Signaling events mediated by VEGFR1 and VEGFR2 | 2.92E-06 |
| The IGF-1 Receptor and Longevity | 2.99E-06 |
| MAPK signaling pathway | 3.00E-06 |
| IL-4 signaling Pathway | 3.01E-06 |
| phosphorylation | 2.73E-06 |
| LPA receptor mediated events | 3.14E-06 |
| Glioma | 3.14E-06 |
| Cadmium induces DNA synthesis and proliferation in macrophages | 3.45E-06 |
| Role of nicotinic acetylcholine receptors in the regulation of apoptosis | 3.45E-06 |
| core promoter binding | 4.23E-06 |
| PI3K-Akt signaling pathway | 3.47E-06 |
| positive regulation of phosphorylation | 3.09E-06 |
| AMPK signaling | 3.62E-06 |
| IL-5 Signaling Pathway | 3.80E-06 |
| cellular response to oxygen-containing compound | 3.47E-06 |
| Alpha6-Beta4 Integrin Signaling Pathway | 3.99E-06 |
| regulation of fibroblast proliferation | 3.55E-06 |
| response to nitrogen compound | 3.55E-06 |
| Melanoma | 4.18E-06 |
| Prostatic Neoplasms | 5.06E-06 |
| Signaling events mediated by Hepatocyte Growth Factor Receptor (c-Met) | 4.38E-06 |
| Telomeres, Telomerase, Cellular Aging, and Immortality | 4.50E-06 |
| PTEN dependent cell cycle arrest and apoptosis | 4.50E-06 |
| Role of EGF Receptor Transactivation by GPCRs in Cardiac Hypertrophy | 4.50E-06 |
| Nerve growth factor pathway (NGF) | 4.50E-06 |
| Role of Erk5 in Neuronal Survival | 4.50E-06 |
| fibroblast proliferation | 4.00E-06 |
| E-cadherin signaling in keratinocytes | 5.29E-06 |
| Fc epsilon receptor (FCERI) signaling | 5.44E-06 |
| Fc receptor signaling pathway | 5.06E-06 |
| Regulation of Wnt-mediated beta catenin signaling and target gene transcription | 5.92E-06 |
| Skeletal muscle hypertrophy is regulated via AKT/mTOR pathway | 6.05E-06 |
| Signaling Pathways in Glioblastoma | 6.11E-06 |
| protein phosphatase binding | 7.75E-06 |
| regulation of cell proliferation | 5.61E-06 |
| regulation of neuron death | 5.88E-06 |
| CD28 dependent PI3K/Akt signaling | 6.87E-06 |
| IGF-1 Signaling Pathway | 6.87E-06 |
| Viral carcinogenesis | 6.95E-06 |
| Delta-Notch Signaling Pathway | 7.53E-06 |
| Signaling events mediated by the Hedgehog family | 7.55E-06 |
| Role of ERBB2 in Signal Transduction and Oncology | 7.55E-06 |
| PDGFR-alpha signaling pathway | 7.55E-06 |
| Insulin Signaling Pathway | 7.55E-06 |
| negative regulation of signal transduction | 6.79E-06 |
| cellular response to external stimulus | 6.79E-06 |
| ErbB signaling pathway | 8.31E-06 |
| mTOR Signaling Pathway | 8.40E-06 |
| Multiple antiapoptotic pathways from IGF-1R signaling lead to BAD phosphorylation | 8.40E-06 |
| VEGFR3 signaling in lymphatic endothelium | 8.40E-06 |
| regulation of glucose transport | 7.41E-06 |
| Corticotropin-releasing hormone | 8.80E-06 |
| regulation of smooth muscle cell proliferation | 7.91E-06 |
| TCR Signaling Pathway | 9.13E-06 |
| CXCR4 Signaling Pathway | 9.23E-06 |
| TPO Signaling Pathway | 9.23E-06 |
| Regulation of eIF4e and p70 S6 Kinase | 9.23E-06 |
| Interleukin signaling pathway | 9.24E-06 |
| intrinsic apoptotic signaling pathway | 8.52E-06 |
| muscle structure development | 8.52E-06 |
| smooth muscle cell proliferation | 8.52E-06 |
| negative regulation of signaling | 8.69E-06 |
| negative regulation of cell communication | 8.69E-06 |
| Nectin adhesion pathway | 1.03E-05 |
| neurotrophin TRK receptor signaling pathway | 9.22E-06 |
| neurotrophin signaling pathway | 9.59E-06 |
| neuron death | 9.79E-06 |
| regulation of myeloid leukocyte differentiation | 9.83E-06 |
| Nephrin/Neph1 signaling in the kidney podocyte | 1.13E-05 |
| Regulation of BAD phosphorylation | 1.13E-05 |
| WNT Signaling Pathway | 1.13E-05 |
| Hypoxia response via HIF activation | 1.13E-05 |
| Phospholipids as signalling intermediaries | 1.24E-05 |
| Transcription factor CREB and its extracellular signals | 1.24E-05 |
| Genes related to IL4 rceptor signaling in B lymphocytes | 1.24E-05 |
| response to abiotic stimulus | 1.10E-05 |
| Neural Crest Differentiation | 1.26E-05 |
| phosphatidylinositol 3-kinase complex, class IA | 1.75E-05 |
| IGF1 pathway | 1.37E-05 |
| IL-3 Signaling Pathway | 1.39E-05 |
| response to UV | 1.23E-05 |
| HIF-1 signaling pathway | 1.48E-05 |
| Thyroid cancer | 1.48E-05 |
| Reelin signaling pathway | 1.48E-05 |
| VEGFR1 specific signals | 1.48E-05 |
| cell projection | 2.05E-05 |
| neuron part | 2.05E-05 |
| cellular response to organic cyclic compound | 1.41E-05 |
| IL2 signaling events mediated by STAT5 | 1.61E-05 |
| CD40/CD40L signaling | 1.61E-05 |
| Control of skeletal myogenesis by HDAC and calcium/calmodulin-dependent kinase (CaMK) | 1.61E-05 |
| MAPK signaling pathway | 1.66E-05 |
| intracellular steroid hormone receptor signaling pathway | 1.46E-05 |
| epithelial cell proliferation | 1.46E-05 |
| EGF Signaling Pathway | 1.74E-05 |
| a6b1 and a6b4 Integrin signaling | 1.74E-05 |
| Hemangiosarcoma | 2.12E-05 |
| Cholinergic synapse | 1.80E-05 |
| GPVI-mediated activation cascade | 1.86E-05 |
| Trk receptor signaling mediated by PI3K and PLC-gamma | 1.86E-05 |
| CD28 co-stimulation | 1.86E-05 |
| PDGF Signaling Pathway | 1.86E-05 |
| cellular response to peptide hormone stimulus | 1.63E-05 |
| response to light stimulus | 1.66E-05 |
| negative regulation of cellular metabolic process | 1.68E-05 |
| epithelium development | 1.71E-05 |
| MAPK cascade | 1.73E-05 |
| phosphatase binding | 2.47E-05 |
| Intracellular Signalling Through Adenosine Receptor A2a and Adenosine | 2.02E-05 |
| N-cadherin signaling events | 2.02E-05 |
| transcriptional activator activity, RNA polymerase II core promoter proximal region sequence-specific binding | 2.49E-05 |
| cellular response to peptide | 1.82E-05 |
| response to oxidative stress | 1.86E-05 |
| positive regulation of multicellular organismal process | 1.86E-05 |
| Insulin/IGF pathway-protein kinase B signaling cascade | 2.13E-05 |
| Intracellular Signalling Through Adenosine Receptor A2b and Adenosine | 2.13E-05 |
| Genes related to CD40 signaling | 2.13E-05 |
| CXCR3-mediated signaling events | 2.13E-05 |
| phosphatidylinositol 3-kinase-Akt signaling | 2.13E-05 |
| Gastric cancer network 2 | 2.13E-05 |
| positive regulation of cell differentiation | 1.94E-05 |
| Toxoplasmosis | 2.25E-05 |
| glucose transport | 1.98E-05 |
| IL1-mediated signaling events | 2.29E-05 |
| FAS (CD95) signaling pathway | 2.29E-05 |
| hexose transport | 2.02E-05 |
| Signalling by NGF | 2.31E-05 |
| wound healing | 2.04E-05 |
| monosaccharide transport | 2.09E-05 |
| negative regulation of smooth muscle cell proliferation | 2.11E-05 |
| RNA polymerase II core promoter proximal region sequence-specific DNA binding | 3.01E-05 |
| chromatin binding | 3.01E-05 |
| signal transduction by protein phosphorylation | 2.19E-05 |
| immune response-regulating cell surface receptor signaling pathway | 2.20E-05 |
| regulation of sequence-specific DNA binding transcription factor activity | 2.21E-05 |
| PDGF signaling pathway | 2.58E-05 |
| Signaling of Hepatocyte Growth Factor Receptor | 2.65E-05 |
| IL23-mediated signaling events | 2.65E-05 |
| core promoter proximal region sequence-specific DNA binding | 3.30E-05 |
| core promoter proximal region DNA binding | 3.34E-05 |
| MicroRNAs in cancer | 2.77E-05 |
| repressing transcription factor binding | 3.57E-05 |
| muscle cell proliferation | 2.55E-05 |
| insulin-like growth factor receptor signaling pathway | 2.57E-05 |
| response to lipid | 2.59E-05 |
| FOXM1 transcription factor network | 3.06E-05 |
| Fc Epsilon Receptor I Signaling in Mast Cells | 3.06E-05 |
| cell proliferation | 2.79E-05 |
| positive regulation of immune system process | 2.80E-05 |
| regulation of phosphate metabolic process | 2.80E-05 |
| Signal Transduction | 3.22E-05 |
| response to steroid hormone | 2.84E-05 |
| negative regulation of myeloid leukocyte differentiation | 2.84E-05 |
| IFN-gamma pathway | 3.27E-05 |
| regulation of phosphorus metabolic process | 2.86E-05 |
| regulation of establishment of protein localization to plasma membrane | 3.01E-05 |
| enzyme binding | 4.38E-05 |
| regulation of protein localization to plasma membrane | 3.21E-05 |
| LKB1 signaling events | 3.78E-05 |
| fungiform papilla morphogenesis | 3.42E-05 |
| fungiform papilla formation | 3.42E-05 |
| altered phosphatodylinositol 3-kinase-Akt signaling | 3.92E-05 |
| Insulin Pathway | 3.99E-05 |
| FOXA1 transcription factor network | 3.99E-05 |
| regulation of organ morphogenesis | 3.55E-05 |
| transcriptional activator activity, RNA polymerase II transcription regulatory region sequence-specific binding | 4.99E-05 |
| transcription factor activity, RNA polymerase II core promoter proximal region sequence-specific binding | 4.99E-05 |
| nitric-oxide synthase regulator activity | 4.99E-05 |
| cellular response to UV | 3.57E-05 |
| Ceramide signaling pathway | 4.25E-05 |
| carbohydrate transport | 3.74E-05 |
| negative regulation of neuron death | 3.80E-05 |
| Signaling by ERBB4 | 4.46E-05 |
| Carbohydrate digestion and absorption | 4.46E-05 |
| Differentiation Pathway in PC12 Cells; this is a specific case of PAC1 Receptor Pathway. | 4.46E-05 |
| Genes related to chemotaxis | 4.46E-05 |
| response to oxygen-containing compound | 3.91E-05 |
| cellular response to oxidative stress | 3.91E-05 |
| hematopoietic or lymphoid organ development | 3.98E-05 |
| Non-alcoholic fatty liver disease (NAFLD) | 4.66E-05 |
| Members of the BCR signaling pathway | 4.69E-05 |
| Integrins in angiogenesis | 4.69E-05 |
| core promoter sequence-specific DNA binding | 6.03E-05 |
| Downstream TCR signaling | 4.98E-05 |
| regulation of monooxygenase activity | 4.39E-05 |
| T cell activation | 4.39E-05 |
| positive regulation of cell proliferation | 4.48E-05 |
| branching morphogenesis of an epithelial tube | 4.48E-05 |
| female pregnancy | 4.48E-05 |
| regulation of myeloid cell differentiation | 4.48E-05 |
| genitalia development | 4.48E-05 |
| negative regulation of apoptotic signaling pathway | 4.54E-05 |
| Jak-STAT signaling pathway | 5.19E-05 |
| PI3 kinase pathway | 5.25E-05 |
| response to peptide hormone | 4.60E-05 |
| Liver Neoplasms | 6.51E-05 |
| Carcinoma, Squamous Cell | 6.53E-05 |
| Infertility, Female | 6.53E-05 |
| immune system development | 4.90E-05 |
| fungiform papilla development | 4.90E-05 |
| fibroblast growth factor receptor signaling pathway | 4.90E-05 |
| SIDS Susceptibility Pathways | 5.68E-05 |
| response to radiation | 5.15E-05 |
| myeloid leukocyte differentiation | 5.25E-05 |
| Insulin Signaling | 6.08E-05 |
| prostate gland development | 5.35E-05 |
| response to peptide | 5.37E-05 |
| Genes related to the insulin receptor pathway | 6.22E-05 |
| transforming growth factor beta receptor signaling pathway | 5.45E-05 |
| regulation of cell cycle | 5.45E-05 |
| immune response | 5.50E-05 |
| hormone binding | 7.75E-05 |
| leukocyte differentiation | 5.62E-05 |
| Signaling events mediated by PTP1B | 6.53E-05 |
| Signaling events mediated by Stem cell factor receptor (c-Kit) | 6.53E-05 |
| transcription initiation from RNA polymerase II promoter | 5.86E-05 |
| cellular response to organonitrogen compound | 6.08E-05 |
| innate immune response | 6.13E-05 |
| regulation of morphogenesis of a branching structure | 6.21E-05 |
| maternal process involved in female pregnancy | 6.21E-05 |
| steroid hormone receptor binding | 8.72E-05 |
| Downstream Signaling Events Of B Cell Receptor (BCR) | 7.20E-05 |
| NFAT and Hypertrophy of the heart (Transcription in the broken heart) | 7.24E-05 |
| cellular response to fibroblast growth factor stimulus | 6.46E-05 |
| mammary gland branching involved in pregnancy | 6.46E-05 |
| canonical Wnt signaling pathway involved in negative regulation of apoptotic process | 6.46E-05 |
| hair follicle placode formation | 6.46E-05 |
| regulation of neuron apoptotic process | 6.55E-05 |
| response to fibroblast growth factor | 6.63E-05 |
| TGF Beta Signaling Pathway | 7.62E-05 |
| immune response-regulating signaling pathway | 6.82E-05 |
| morphogenesis of a branching epithelium | 6.92E-05 |
| Coregulation of Androgen receptor activity | 8.40E-05 |
| Transcriptional misregulation in cancer | 8.40E-05 |
| Influenza A | 8.40E-05 |
| canonical Wnt signaling pathway | 7.40E-05 |
| positive regulation of carbohydrate metabolic process | 7.49E-05 |
| Mechanism of Gene Regulation by Peroxisome Proliferators via PPARa(alpha) | 8.72E-05 |
| VEGF signaling pathway | 8.72E-05 |
| regulation of transport | 7.71E-05 |
| regulation of intracellular signal transduction | 7.71E-05 |
| regulation of osteoclast differentiation | 7.71E-05 |
| positive regulation of sequence-specific DNA binding transcription factor activity | 7.93E-05 |
| cellular response to nitrogen compound | 7.93E-05 |
| androgen receptor signaling pathway | 7.93E-05 |
| growth | 7.96E-05 |
| morphogenesis of a branching structure | 8.16E-05 |
| cellular response to transforming growth factor beta stimulus | 8.16E-05 |
| response to transforming growth factor beta | 8.25E-05 |
| mTOR signaling pathway | 9.62E-05 |
| neuron projection | 1.31E-04 |
| VEGF signaling pathway | 1.01E-04 |
| negative regulation of lipid metabolic process | 8.83E-05 |
| Chemokine signaling pathway | 1.02E-04 |
| blood coagulation | 8.93E-05 |
| epidermal growth factor receptor signaling pathway | 8.93E-05 |
| neuron apoptotic process | 8.93E-05 |
| coagulation | 9.08E-05 |
| multi-multicellular organism process | 9.08E-05 |
| regulation of leukocyte differentiation | 9.08E-05 |
| hemostasis | 9.10E-05 |
| DNA-templated transcription, initiation | 9.12E-05 |
| Carcinoma of colon | 1.25E-04 |
| Penile Neoplasms | 1.25E-04 |
| ERBB signaling pathway | 9.38E-05 |
| cellular response to mechanical stimulus | 9.50E-05 |
| IL4-mediated signaling events | 1.10E-04 |
| 1-phosphatidylinositol-3-kinase activity | 1.36E-04 |
| glucose metabolic process | 9.74E-05 |
| TCR signaling | 1.15E-04 |
| tongue morphogenesis | 1.01E-04 |
| T cell costimulation | 1.05E-04 |
| regulation of protein localization | 1.05E-04 |
| lymphocyte costimulation | 1.09E-04 |
| lymphocyte apoptotic process | 1.09E-04 |
| Renal cell carcinoma | 1.25E-04 |
| insulin receptor substrate binding | 1.55E-04 |
| negative regulation of leukocyte differentiation | 1.12E-04 |
| mammary gland epithelium development | 1.12E-04 |
| Genes related to PIP3 signaling in cardiac myocytes | 1.30E-04 |
| negative regulation of cell differentiation | 1.16E-04 |
| Focal adhesion | 1.39E-04 |
| regulation of MAPK cascade | 1.23E-04 |
| embryonic morphogenesis | 1.23E-04 |
| regulation of oxidoreductase activity | 1.23E-04 |
| Double Stranded RNA Induced Gene Expression | 1.47E-04 |
| Fc epsilon RI signaling pathway | 1.47E-04 |
| CDC42 signaling events | 1.52E-04 |
| lymphocyte activation | 1.33E-04 |
| epithelial cell differentiation | 1.33E-04 |
| regulation of lipid metabolic process | 1.35E-04 |
| Rap1 signaling pathway | 1.56E-04 |
| Costimulation by the CD28 family | 1.57E-04 |
| regulation of epithelial cell proliferation | 1.38E-04 |
| regulation of T cell activation | 1.40E-04 |
| embryo development | 1.40E-04 |
| cellular response to light stimulus | 1.42E-04 |
| negative regulation of myeloid cell differentiation | 1.42E-04 |
| Endothelin signaling pathway | 1.63E-04 |
| Signaling by the B Cell Receptor (BCR) | 1.65E-04 |
| regulation of cellular component organization | 1.49E-04 |
| hexose metabolic process | 1.51E-04 |
| Infertility, Male | 2.07E-04 |
| mitochondrial membrane organization | 1.56E-04 |
| CXCR4-mediated signaling events | 1.82E-04 |
| Bacterial invasion of epithelial cells | 1.82E-04 |
| intracellular receptor signaling pathway | 1.69E-04 |
| positive regulation of signal transduction | 1.70E-04 |
| Ras signaling pathway | 1.94E-04 |
| regulation of stem cell proliferation | 1.70E-04 |
| negative regulation of gene expression | 1.72E-04 |
| osteoclast differentiation | 1.75E-04 |
| hair follicle development | 1.84E-04 |
| molting cycle process | 1.84E-04 |
| hair cycle process | 1.84E-04 |
| regulation of reproductive process | 1.89E-04 |
| negative regulation of macromolecule biosynthetic process | 2.03E-04 |
| BREAST-OVARIAN CANCER, FAMILIAL, SUSCEPTIBILITY TO, 1; BROVCA1 | 2.81E-04 |
| negative regulation of transcription from RNA polymerase II promoter | 2.15E-04 |
| Hemostasis | 2.52E-04 |
| establishment of protein localization to membrane | 2.23E-04 |
| regulation of body fluid levels | 2.23E-04 |
| positive regulation of signaling | 2.27E-04 |
| Progesterone-mediated oocyte maturation | 2.59E-04 |
| RB in Cancer | 2.65E-04 |
| Constitutive PI3K/AKT Signaling in Cancer | 2.65E-04 |
| MAPKinase Signaling Pathway | 2.65E-04 |
| prostate gland growth | 2.32E-04 |
| positive regulation of cell communication | 2.33E-04 |
| monosaccharide metabolic process | 2.34E-04 |
| single-organism membrane organization | 2.35E-04 |
| Colonic Neoplasms | 3.19E-04 |
| establishment of protein localization to plasma membrane | 2.38E-04 |
| Bone Remodelling | 2.77E-04 |
| leukocyte apoptotic process | 2.44E-04 |
| urogenital system development | 2.48E-04 |
| leukocyte activation | 2.55E-04 |
| transmembrane receptor protein serine/threonine kinase signaling pathway | 2.58E-04 |
| positive regulation of nitric-oxide synthase activity | 2.58E-04 |
| epithelial cell differentiation involved in prostate gland development | 2.58E-04 |
| genitalia morphogenesis | 2.58E-04 |
| phosphatidylinositol 3-kinase activity | 3.65E-04 |
| Fc gamma R-mediated phagocytosis | 3.00E-04 |
| negative regulation of biosynthetic process | 2.64E-04 |
| muscle cell differentiation | 2.64E-04 |
| Multi-step Regulation of Transcription by Pitx2 | 3.15E-04 |
| TFs Regulate miRNAs related to cardiac hypertrophy | 3.15E-04 |
| R-SMAD binding | 3.92E-04 |
| negative regulation of developmental process | 2.85E-04 |
| hair cycle | 2.87E-04 |
| molting cycle | 2.87E-04 |
| ectodermal placode formation | 2.87E-04 |
| ectodermal placode morphogenesis | 2.87E-04 |
| ectodermal placode development | 2.87E-04 |
| single-organism carbohydrate metabolic process | 2.91E-04 |
| cell development | 2.92E-04 |
| system process | 2.99E-04 |
| regulation of cell development | 2.99E-04 |
| myeloid cell differentiation | 2.99E-04 |
| intrinsic apoptotic signaling pathway in response to DNA damage | 3.01E-04 |
| T cell receptor signaling pathway | 3.01E-04 |
| epithelial tube morphogenesis | 3.06E-04 |
| RNA polymerase II transcription factor activity, sequence-specific DNA binding | 4.30E-04 |
| nuclear hormone receptor binding | 4.30E-04 |
| negative regulation of intracellular signal transduction | 3.08E-04 |
| phosphatidylinositol kinase activity | 4.32E-04 |
| Role of PI3K subunit p85 in regulation of Actin Organization and Cell Migration | 3.57E-04 |
| p53 signaling pathway | 3.57E-04 |
| phosphatidylinositol-3-phosphate biosynthetic process | 3.13E-04 |
| negative regulation of glucose import | 3.13E-04 |
| transcription factor complex | 4.83E-04 |
| Wnt signaling pathway | 3.15E-04 |
| positive regulation of cellular component organization | 3.25E-04 |
| striated muscle tissue development | 3.25E-04 |
| regulation of lymphocyte activation | 3.25E-04 |
| PIP3 activates AKT signaling | 3.71E-04 |
| PI3K events in ERBB4 signaling | 3.71E-04 |
| PI3K events in ERBB2 signaling | 3.71E-04 |
| PI3K/AKT Signaling in Cancer | 3.71E-04 |
| PI-3K cascade | 3.71E-04 |
| regulation of organelle organization | 3.30E-04 |
| Carcinoma | 4.56E-04 |
| Sarcoma | 4.56E-04 |
| Wnt Signaling Pathway and Pluripotency | 3.88E-04 |
| PTEN is a tumor suppressor that dephosphorylates the lipid messenger phosphatidylinositol triphosphate. | 3.88E-04 |
| IL-7 Signal Transduction | 3.88E-04 |
| EPHA2 forward signaling | 3.88E-04 |
| The TrkA receptor binds nerve growth factor to activate MAP kinase pathways and promote cell growth. | 3.88E-04 |
| hemopoiesis | 3.39E-04 |
| fibroblast apoptotic process | 3.43E-04 |
| positive regulation of lymphocyte apoptotic process | 3.43E-04 |
| negative regulation of cell cycle | 3.46E-04 |
| tube morphogenesis | 3.46E-04 |
| PI3K/AKT activation | 3.96E-04 |
| GAB1 signalosome | 4.06E-04 |
| muscle organ development | 3.55E-04 |
| chromatin remodeling | 3.62E-04 |
| muscle tissue development | 3.76E-04 |
| Regulation of signaling by CBL | 4.31E-04 |
| morphogenesis of an epithelial bud | 3.77E-04 |
| regulation of muscle cell differentiation | 3.85E-04 |
| enzyme regulator activity | 5.46E-04 |
| regulation of intrinsic apoptotic signaling pathway | 3.92E-04 |
| immune response-activating signal transduction | 3.98E-04 |
| cellular response to radiation | 3.98E-04 |
| Amoebiasis | 4.68E-04 |
| Chaperones modulate interferon Signaling Pathway | 4.68E-04 |
| Tie2 Signaling | 4.68E-04 |
| Signaling by FGFR1 fusion mutants | 4.68E-04 |
| Downregulated of MTA-3 in ER-negative Breast Tumors | 4.68E-04 |
| Paxillin-dependent events mediated by a4b1 | 4.68E-04 |
| TP53 network | 4.68E-04 |
| protein complex binding | 5.74E-04 |
| regulation of cell fate specification | 4.11E-04 |
| negative regulation of glucose transport | 4.11E-04 |
| protein localization to membrane | 4.12E-04 |
| hormone receptor binding | 5.84E-04 |
| regulation of immune response | 4.18E-04 |
| positive regulation of cell cycle | 4.28E-04 |
| protein localization to plasma membrane | 4.36E-04 |
| Spinal Cord Injury | 5.02E-04 |
| phosphatidylinositol 3-kinase complex | 6.75E-04 |
| positive regulation of monooxygenase activity | 4.47E-04 |
| negative regulation of osteoclast differentiation | 4.47E-04 |
| Ras-Independent pathway in NK cell-mediated cytotoxicity | 5.11E-04 |
| ATM Signaling Pathway | 5.11E-04 |
| Signaling by constitutively active EGFR | 5.11E-04 |
| negative regulation of transport | 4.52E-04 |
| in utero embryonic development | 4.52E-04 |
| Signaling by NOTCH | 5.21E-04 |
| Wnt signaling pathway | 5.24E-04 |
| Signaling by Interleukins | 5.44E-04 |
| stem cell proliferation | 4.77E-04 |
| regulation of leukocyte activation | 4.80E-04 |
| The Co-Stimulatory Signal During T-cell Activation | 5.54E-04 |
| p53 pathway by glucose deprivation | 5.54E-04 |
| Leukocyte transendothelial migration | 5.66E-04 |
| protein kinase binding | 7.05E-04 |
| membrane organization | 5.05E-04 |
| Prostatic Intraepithelial Neoplasia | 7.01E-04 |
| mammary gland development | 5.23E-04 |
| regulation of chromosome organization | 5.23E-04 |
| mammary gland alveolus development | 5.23E-04 |
| tongue development | 5.23E-04 |
| mammary gland lobule development | 5.23E-04 |
| Paxillin-independent events mediated by a4b1 and a4b7 | 6.02E-04 |
| Apoptotic Signaling in Response to DNA Damage | 6.02E-04 |
| response to drug | 5.44E-04 |
| antigen receptor-mediated signaling pathway | 5.46E-04 |
| Huntington disease | 6.32E-04 |
| gland development | 5.55E-04 |
| Role of LAT2/NTAL/LAB on calcium mobilization | 6.42E-04 |
| Cell cycle | 6.42E-04 |
| Angiogenesis | 6.42E-04 |
| Rac 1 cell motility signaling pathway | 6.42E-04 |
| Hypoxia and p53 in the Cardiovascular system | 6.42E-04 |
| Nephrin interactions | 6.42E-04 |
| positive regulation of establishment of protein localization to plasma membrane | 5.63E-04 |
| mesenchyme morphogenesis | 5.63E-04 |
| protein phosphatase 2A binding | 7.89E-04 |
| protein C-terminus binding | 8.02E-04 |
| development of primary male sexual characteristics | 5.83E-04 |
| chromosome organization | 5.84E-04 |
| positive regulation of epithelial cell proliferation | 5.90E-04 |
| activation of immune response | 5.90E-04 |
| cellular response to stress | 6.00E-04 |
| male genitalia development | 6.00E-04 |
| single-organism biosynthetic process | 6.00E-04 |
| regulation of cell activation | 6.03E-04 |
| Adrenal Gland Neoplasms | 8.22E-04 |
| IL-9 Signaling Pathway | 6.95E-04 |
| Erk and PI-3 Kinase Are Necessary for Collagen Binding in Corneal Epithelia | 6.95E-04 |
| blood circulation | 6.12E-04 |
| positive regulation of apoptotic process | 6.15E-04 |
| circulatory system process | 6.16E-04 |
| embryonic organ development | 6.16E-04 |
| negative regulation of autophagy | 6.38E-04 |
| defense response | 6.41E-04 |
| positive regulation of programmed cell death | 6.43E-04 |
| carbohydrate metabolic process | 6.44E-04 |
| Cell-Cell communication | 7.37E-04 |
| cell growth | 6.50E-04 |
| Retinoic acid receptors-mediated signaling | 7.44E-04 |
| EGF receptor (ErbB1) signaling pathway | 7.44E-04 |
| TRAIL signaling pathway | 7.44E-04 |
| male sex differentiation | 6.53E-04 |
| single-organism localization | 6.58E-04 |
| single-organism cellular localization | 6.58E-04 |
| regulation of carbohydrate metabolic process | 6.58E-04 |
| organ induction | 6.70E-04 |
| cell activation | 6.84E-04 |
| reproductive structure development | 6.92E-04 |
| organ morphogenesis | 6.93E-04 |
| morphogenesis of an epithelium | 6.93E-04 |
| Heat Stroke | 9.47E-04 |
| Interleukin 4 (IL-4) Pathway | 8.03E-04 |
| reproductive system development | 7.03E-04 |
| positive regulation of protein insertion into mitochondrial membrane involved in apoptotic signaling pathway | 7.03E-04 |
| positive regulation of leukocyte apoptotic process | 7.03E-04 |
| branching involved in mammary gland duct morphogenesis | 7.03E-04 |
| regulation of protein insertion into mitochondrial membrane involved in apoptotic signaling pathway | 7.03E-04 |
| mitotic cell cycle | 7.03E-04 |
| kinase binding | 9.98E-04 |
| regulation of establishment of protein localization | 7.24E-04 |
| multi-organism reproductive process | 7.32E-04 |
| Signaling by SCF-KIT | 8.43E-04 |
| negative regulation of lipid transport | 7.42E-04 |
| morphogenesis of an epithelial fold | 7.42E-04 |
| regulation of neuroblast proliferation | 7.42E-04 |
| Wnt signaling pathway | 8.51E-04 |
| Ephrin B reverse signaling | 8.51E-04 |
| Neuropeptides VIP and PACAP inhibit the apoptosis of activated T cells | 8.51E-04 |
| 3-phosphoinositide biosynthesis | 8.51E-04 |
| lipid modification | 7.56E-04 |
| Notch signaling pathway | 7.56E-04 |
| Insulin signaling pathway | 8.83E-04 |
| androgen receptor binding | 1.08E-03 |
| multicellular organism growth | 7.80E-04 |
| negative regulation of stress-activated protein kinase signaling cascade | 7.80E-04 |
| negative regulation of stress-activated MAPK cascade | 7.80E-04 |
| protein insertion into mitochondrial membrane involved in apoptotic signaling pathway | 7.80E-04 |
| negative regulation of fibroblast proliferation | 7.80E-04 |
| positive regulation of cell death | 7.82E-04 |
| Cell Cycle: G1/S Check Point | 9.00E-04 |
| G alpha q Pathway | 9.00E-04 |
| Growth Hormone Signaling Pathway | 9.00E-04 |
| Activation of BH3-only proteins | 9.00E-04 |
| glutamate receptor binding | 1.11E-03 |
| Downstream signaling of activated FGFR | 9.23E-04 |
| estrogen receptor binding | 1.14E-03 |
| cell cycle | 8.17E-04 |
| protein insertion into mitochondrial membrane | 8.27E-04 |
| positive regulation of molecular function | 8.27E-04 |
| regulation of cellular protein metabolic process | 8.31E-04 |
| regulation of cellular localization | 8.31E-04 |
| negative regulation of molecular function | 8.31E-04 |
| skeletal muscle tissue development | 8.31E-04 |
| Urinary Bladder Neoplasms | 1.13E-03 |
| Ovarian Neoplasms | 1.13E-03 |
| Colorectal Neoplasms | 1.13E-03 |
| Adenocarcinoma Of Esophagus | 1.13E-03 |
| Interleukin receptor SHC signaling | 9.56E-04 |
| VEGF, Hypoxia, and Angiogenesis | 9.56E-04 |
| plasma membrane organization | 8.40E-04 |
| glial cell differentiation | 8.40E-04 |
| regulation of cell cycle process | 8.61E-04 |
| regulation of oligodendrocyte differentiation | 8.61E-04 |
| negative regulation of transcription, DNA-templated | 8.69E-04 |
| insulin receptor signaling pathway | 8.72E-04 |
| Adrenergic signaling in cardiomyocytes | 1.01E-03 |
| Axon guidance mediated by netrin | 1.01E-03 |
| Signaling by FGFR1 mutants | 1.01E-03 |
| regulation of cell fate commitment | 9.06E-04 |
| prostate gland epithelium morphogenesis | 9.06E-04 |
| skeletal muscle organ development | 9.06E-04 |
| RNA polymerase II core promoter sequence-specific DNA binding | 1.29E-03 |
| Burkitt Lymphoma | 1.26E-03 |
| Aurora A signaling | 1.07E-03 |
| SMAD2/SMAD3:SMAD4 heterotrimer regulates transcription | 1.07E-03 |
| IL17 signaling pathway | 1.07E-03 |
| negative regulation of RNA biosynthetic process | 9.39E-04 |
| regulation of response to stress | 9.39E-04 |
| anatomical structure formation involved in morphogenesis | 9.54E-04 |
| Liver Neoplasms, Experimental | 1.32E-03 |
| Downstream signal transduction | 1.13E-03 |
| ErbB4 signaling events | 1.13E-03 |
| Oxidative Stress | 1.13E-03 |
| protein dimerization activity | 1.39E-03 |
| receptor tyrosine kinase binding | 1.40E-03 |
| phosphatidylinositol phosphorylation | 1.01E-03 |
| prostate gland morphogenesis | 1.01E-03 |
| positive regulation of T cell activation | 1.01E-03 |
| positive regulation of immune response | 1.01E-03 |
| Signaling by FGFR | 1.16E-03 |
| Signaling by ERBB2 | 1.17E-03 |
| B Cell Receptor Signaling Pathway | 1.17E-03 |
| DAP12 signaling | 1.17E-03 |
| EPHB forward signaling | 1.17E-03 |
| EPO signaling pathway | 1.17E-03 |
| Synthesis of PIPs at the plasma membrane | 1.17E-03 |
| Signaling of Hepatocyte Growth Factor Receptor | 1.17E-03 |
| transcription factor activity, direct ligand regulated sequence-specific DNA binding | 1.46E-03 |
| RNA polymerase II transcription factor activity, ligand-activated sequence-specific DNA binding | 1.46E-03 |
| positive regulation of glucose import | 1.06E-03 |
| Apoptosis | 1.22E-03 |
| negative regulation of RNA metabolic process | 1.08E-03 |
| Wnt/beta-catenin Pathway | 1.23E-03 |
| Internalization of ErbB1 | 1.23E-03 |
| epithelial cell development | 1.09E-03 |
| Adrenocortical Carcinoma | 1.49E-03 |
| Psychoses, Substance-Induced | 1.49E-03 |
| regulation of polysaccharide biosynthetic process | 1.11E-03 |
| positive regulation of mesenchymal cell proliferation | 1.11E-03 |
| chromatin modification | 1.11E-03 |
| CARM1 and Regulation of the Estrogen Receptor | 1.30E-03 |
| Signaling mediated by p38-alpha and p38-beta | 1.30E-03 |
| Integrated Cancer pathway | 1.30E-03 |
| hyaluronan metabolic process | 1.16E-03 |
| regulation of nitric-oxide synthase activity | 1.16E-03 |
| specification of organ identity | 1.16E-03 |
| Signaling events mediated by TCPTP | 1.36E-03 |
| Genes related to PIP3 signaling in B lymphocytes | 1.36E-03 |
| Nasopharyngeal carcinoma | 1.62E-03 |
| activating transcription factor binding | 1.71E-03 |
| steroid hormone receptor activity | 1.71E-03 |
| positive regulation of reproductive process | 1.22E-03 |
| positive regulation of oxidoreductase activity | 1.22E-03 |
| cell migration | 1.22E-03 |
| negative regulation of cellular protein metabolic process | 1.23E-03 |
| p38 MAPK Pathway | 1.42E-03 |
| Thrombin signaling and protease-activated receptors | 1.42E-03 |
| G alpha 13 Pathway | 1.42E-03 |
| PI3K Pathway | 1.42E-03 |
| Toll-Like Receptor Pathway | 1.42E-03 |
| Signaling by FGFR in disease | 1.42E-03 |
| phosphatidylinositol-mediated signaling | 1.25E-03 |
| lipid phosphorylation | 1.26E-03 |
| mammary gland duct morphogenesis | 1.26E-03 |
| regulation of organ formation | 1.26E-03 |
| inositol lipid-mediated signaling | 1.26E-03 |
| Hyperplasia | 1.72E-03 |
| Cardiomyopathy, Hypertrophic | 1.72E-03 |
| Signaling events regulated by Ret tyrosine kinase | 1.47E-03 |
| Bladder cancer | 1.47E-03 |
| Nuclear Receptors | 1.47E-03 |
| Signaling by EGFR | 1.47E-03 |
| positive regulation of protein metabolic process | 1.31E-03 |
| developmental induction | 1.31E-03 |
| regulation of DNA biosynthetic process | 1.31E-03 |
| gliogenesis | 1.31E-03 |
| tissue morphogenesis | 1.32E-03 |
| regulation of neurogenesis | 1.32E-03 |
| Signaling by EGFR in Cancer | 1.52E-03 |
| DAP12 interactions | 1.53E-03 |
| Signaling by PDGF | 1.53E-03 |
| Aldosterone-regulated sodium reabsorption | 1.53E-03 |
| cell-cell signaling involved in cell fate commitment | 1.35E-03 |
| positive regulation of glucose transport | 1.35E-03 |
| regulation of release of cytochrome c from mitochondria | 1.35E-03 |
| regulation of mitochondrial outer membrane permeabilization involved in apoptotic signaling pathway | 1.35E-03 |
| intracellular estrogen receptor signaling pathway | 1.35E-03 |
| negative regulation of cellular macromolecule biosynthetic process | 1.35E-03 |
| regulation of mesenchymal cell proliferation | 1.40E-03 |
| positive regulation of nitric oxide biosynthetic process | 1.40E-03 |
| protein insertion into membrane | 1.40E-03 |
| regulation of polysaccharide metabolic process | 1.40E-03 |
| JNK MAPK Pathway | 1.60E-03 |
| Interleukin-11 Signaling Pathway | 1.60E-03 |
| regulation of I-kappaB kinase/NF-kappaB signaling | 1.43E-03 |
| Focal Adhesion | 1.64E-03 |
| protein import into nucleus, translocation | 1.46E-03 |
| Class I PI3K signaling events | 1.67E-03 |
| Intrinsic Pathway for Apoptosis | 1.67E-03 |
| RNA polymerase II transcription factor activity, estrogen-activated sequence-specific DNA binding | 2.06E-03 |
| type 1 metabotropic glutamate receptor binding | 2.06E-03 |
| Herpes simplex infection | 1.73E-03 |
| platelet activation | 1.54E-03 |
| negative regulation of nucleobase-containing compound metabolic process | 1.54E-03 |
| immune response-activating cell surface receptor signaling pathway | 1.55E-03 |
| extrinsic apoptotic signaling pathway | 1.56E-03 |
| lipid biosynthetic process | 1.57E-03 |
| negative regulation of I-kappaB kinase/NF-kappaB signaling | 1.57E-03 |
| regulation of muscle cell apoptotic process | 1.57E-03 |
| Transcriptional activity of SMAD2/SMAD3:SMAD4 heterotrimer | 1.81E-03 |
| Selenium Metabolism and Selenoproteins | 1.81E-03 |
| Interleukin-2 signaling | 1.81E-03 |
| Hedgehog signaling events mediated by Gli proteins | 1.81E-03 |
| single-organism organelle organization | 1.64E-03 |
| IL-7 Signaling Pathway | 1.88E-03 |
| Regulation of Microtubule Cytoskeleton | 1.88E-03 |
| negative regulation of nitrogen compound metabolic process | 1.66E-03 |
| localization of cell | 1.67E-03 |
| cell motility | 1.67E-03 |
| cellular response to insulin stimulus | 1.67E-03 |
| TNF-alpha/NF-kB Signaling Pathway | 1.92E-03 |
| aging | 1.68E-03 |
| response to gamma radiation | 1.68E-03 |
| striated muscle cell differentiation | 1.70E-03 |
| tube development | 1.70E-03 |
| stress-activated MAPK cascade | 1.71E-03 |
| Signaling by FGFR mutants | 1.96E-03 |
| protein targeting to nucleus | 1.72E-03 |
| single-organism nuclear import | 1.72E-03 |
| protein import into nucleus | 1.72E-03 |
| regulation of growth | 1.72E-03 |
| epithelial cell morphogenesis | 1.72E-03 |
| response to cytokine | 1.74E-03 |
| nuclear import | 1.77E-03 |
| stress-activated protein kinase signaling cascade | 1.77E-03 |
| positive regulation of lymphocyte activation | 1.77E-03 |
| mitochondrial outer membrane permeabilization | 1.77E-03 |
| Role of phospholipids in phagocytosis | 2.03E-03 |
| Keratinocyte Differentiation | 2.03E-03 |
| NGF signalling via TRKA from the plasma membrane | 2.04E-03 |
| cell cycle process | 1.79E-03 |
| negative regulation of protein metabolic process | 1.80E-03 |
| I-kappaB kinase/NF-kappaB signaling | 1.80E-03 |
| regulation of nervous system development | 1.81E-03 |
| mesenchymal cell proliferation | 1.81E-03 |
| muscle cell apoptotic process | 1.81E-03 |
| positive regulation of mitochondrial membrane permeability involved in apoptotic process | 1.81E-03 |
| mitochondrial outer membrane permeabilization involved in programmed cell death | 1.81E-03 |
| positive regulation of fibroblast proliferation | 1.81E-03 |
| FoxO family signaling | 2.11E-03 |
| spinobulbar muscular atrophy | 2.11E-03 |
| negative regulation of cellular biosynthetic process | 1.85E-03 |
| localization within membrane | 1.87E-03 |
| Notch-mediated HES/HEY network | 2.17E-03 |
| amb2 Integrin signaling | 2.17E-03 |
| Type II diabetes mellitus | 2.17E-03 |
| NOTCH1 Intracellular Domain Regulates Transcription | 2.17E-03 |
| beta-catenin binding | 2.68E-03 |
| SMAD binding | 2.68E-03 |
| receptor binding | 2.68E-03 |
| T cell apoptotic process | 1.94E-03 |
| positive regulation of mitochondrial membrane permeability | 1.94E-03 |
| cell fate commitment | 1.94E-03 |
| chordate embryonic development | 1.94E-03 |
| Generic Transcription Pathway | 2.25E-03 |
| Interleukin-3, 5 and GM-CSF signaling | 2.25E-03 |
| cellular response to drug | 1.99E-03 |
| negative regulation of proteolysis | 1.99E-03 |
| cellular protein localization | 1.99E-03 |
| developmental process involved in reproduction | 2.01E-03 |
| embryo development ending in birth or egg hatching | 2.02E-03 |
| cellular macromolecule localization | 2.03E-03 |
| neuroblast proliferation | 2.04E-03 |
| regulation of mitochondrial membrane permeability involved in apoptotic process | 2.04E-03 |
| Regulation of Androgen receptor activity | 2.33E-03 |
| chromatin organization | 2.05E-03 |
| Platelet activation, signaling and aggregation | 2.37E-03 |
| positive regulation of cellular carbohydrate metabolic process | 2.10E-03 |
| regulation of nitric oxide biosynthetic process | 2.10E-03 |
| positive regulation of leukocyte activation | 2.10E-03 |
| PI Metabolism | 2.41E-03 |
| development of primary sexual characteristics | 2.12E-03 |
| lipid metabolic process | 2.16E-03 |
| release of cytochrome c from mitochondria | 2.16E-03 |
| reproductive process | 2.18E-03 |
| Id Signaling Pathway | 2.49E-03 |
| Nuclear Receptor transcription pathway | 2.49E-03 |
| regulation of kidney development | 2.21E-03 |
| DNA alkylation | 2.21E-03 |
| DNA methylation | 2.21E-03 |
| histone deacetylation | 2.21E-03 |
| protein import | 2.25E-03 |
| Endothelins | 2.58E-03 |
| response to metal ion | 2.26E-03 |
| mammary gland morphogenesis | 2.26E-03 |
| regulation of lymphocyte apoptotic process | 2.26E-03 |
| positive regulation of protein phosphorylation | 2.26E-03 |
| regulation of DNA metabolic process | 2.26E-03 |
| positive regulation of cell activation | 2.26E-03 |
| regulation of protein modification process | 2.29E-03 |
| regulation of intracellular steroid hormone receptor signaling pathway | 2.32E-03 |
| regulation of myotube differentiation | 2.32E-03 |
| Signaling events mediated by focal adhesion kinase | 2.67E-03 |
| steroid hormone mediated signaling pathway | 2.39E-03 |
| regulation of glial cell differentiation | 2.39E-03 |
| response to alcohol | 2.40E-03 |
| Basal cell carcinoma | 2.76E-03 |
| ErbB signaling pathway | 2.76E-03 |
| positive regulation of mitochondrion organization | 2.44E-03 |
| embryonic digit morphogenesis | 2.44E-03 |
| response to insulin | 2.44E-03 |
| viral process | 2.44E-03 |
| positive regulation of cytokine production | 2.44E-03 |
| hypomethylation of CpG island | 2.44E-03 |
| DNA hypomethylation | 2.44E-03 |
| positive regulation of metanephric cap mesenchymal cell proliferation | 2.44E-03 |
| cellular response to insulin-like growth factor stimulus | 2.44E-03 |
| prostate field specification | 2.44E-03 |
| negative regulation of interleukin-12 biosynthetic process | 2.44E-03 |
| lactic acid secretion | 2.44E-03 |
| glycogen cell differentiation involved in embryonic placenta development | 2.44E-03 |
| prostate induction | 2.44E-03 |
| male somatic sex determination | 2.44E-03 |
| activation of prostate induction by androgen receptor signaling pathway | 2.44E-03 |
| regulation of metanephric cap mesenchymal cell proliferation | 2.44E-03 |
| negative regulation of integrin biosynthetic process | 2.44E-03 |
| myoblast differentiation | 2.44E-03 |
| nuclear chromatin | 3.78E-03 |
| Neurotrophic factor-mediated Trk receptor signaling | 2.85E-03 |
| sex differentiation | 2.51E-03 |
| organ formation | 2.51E-03 |
| regulation of mitochondrial membrane permeability | 2.51E-03 |
| multi-organism cellular process | 2.52E-03 |
| protein localization to nucleus | 2.52E-03 |
| branching involved in ureteric bud morphogenesis | 2.57E-03 |
| regulation of membrane permeability | 2.57E-03 |
| toll-like receptor 10 signaling pathway | 2.64E-03 |
| endoderm development | 2.64E-03 |
| Constitutive Signaling by NOTCH1 HD+PEST Domain Mutants | 3.02E-03 |
| p53 pathway | 3.02E-03 |
| Constitutive Signaling by NOTCH1 PEST Domain Mutants | 3.02E-03 |
| Notch signaling pathway | 3.02E-03 |
| B cell activation | 3.02E-03 |
| lymphocyte differentiation | 2.67E-03 |
| positive regulation of intracellular signal transduction | 2.69E-03 |
| nitric oxide biosynthetic process | 2.70E-03 |
| positive regulation of muscle cell differentiation | 2.70E-03 |
| POU domain binding | 3.85E-03 |
| skeletal muscle cell differentiation | 2.76E-03 |
| toll-like receptor 5 signaling pathway | 2.76E-03 |
| positive regulation of stem cell proliferation | 2.76E-03 |
| Wnt Signaling Pathway | 3.22E-03 |
| leukocyte migration | 2.83E-03 |
| protein deacetylation | 2.83E-03 |
| Leukemia | 3.84E-03 |
| Adenoma | 3.84E-03 |
| positive regulation of cell migration | 2.88E-03 |
| epidermis development | 2.90E-03 |
| steroid binding | 4.10E-03 |
| modification of morphology or physiology of other organism involved in symbiotic interaction | 2.97E-03 |
| DNA methylation or demethylation | 2.97E-03 |
| ureteric bud morphogenesis | 2.97E-03 |
| Neoplasm Metastasis | 4.03E-03 |
| Hypertension | 4.03E-03 |
| Insulin Resistance | 4.03E-03 |
| Medulloblastoma | 4.03E-03 |
| Reifenstein syndrome | 4.03E-03 |
| Nasopharyngeal carcinoma | 4.03E-03 |
| Proteus syndrome | 4.03E-03 |
| Feminization | 4.03E-03 |
| ANDROGEN INSENSITIVITY, PARTIAL | 4.03E-03 |
| Megalencephaly cutis marmorata telangiectatica congenita | 4.03E-03 |
| BURKITT LYMPHOMA; BL | 4.03E-03 |
| KERATOSIS, SEBORRHEIC | 4.03E-03 |
| Bulbo-Spinal Atrophy, X-Linked | 4.03E-03 |
| Androgen resistance syndrome | 4.03E-03 |
| Neuroectodermal Tumors | 4.03E-03 |
| Immunodeficiency 36 | 4.03E-03 |
| Congenital lipomatous overgrowth, vascular malformations, and epidermal nevi | 4.03E-03 |
| Cowden syndrome 6 | 4.03E-03 |
| X-linked hypospadias 1 | 4.03E-03 |
| Papilloma, Choroid Plexus | 4.03E-03 |
| Burkitt lymphoma | 4.03E-03 |
| Mental retardation, autosomal dominant 19 | 4.03E-03 |
| Bulbospinal neuronopathy, X-linked recessive | 4.03E-03 |
| Balkan Nephropathy | 4.03E-03 |
| Keratosis, seborrheic | 4.03E-03 |
| SPINAL AND BULBAR MUSCULAR ATROPHY, X-LINKED 1; SMAX1 | 4.03E-03 |
| Li-Fraumeni syndrome 1 | 4.03E-03 |
| SHORT syndrome | 4.03E-03 |
| Keratosis, Seborrheic | 4.03E-03 |
| Basal cell carcinoma, susceptibility to, 7 | 4.03E-03 |
| Cowden syndrome 5 | 4.03E-03 |
| Estrogen resistance | 4.03E-03 |
| Androgen-Insensitivity Syndrome | 4.03E-03 |
| PAPILLOMA OF CHOROID PLEXUS | 4.03E-03 |
| Agammaglobulinemia 7, autosomal recessive | 4.03E-03 |
| Adrenocortical carcinoma, hereditary | 4.03E-03 |
| Choroid plexus papilloma | 4.03E-03 |
| Bulbo-spinal atrophy X-linked | 4.03E-03 |
| Mouth Neoplasms | 4.03E-03 |
| regulation of anatomical structure morphogenesis | 2.98E-03 |
| Validated targets of C-MYC transcriptional repression | 3.41E-03 |
| IL12-mediated signaling events | 3.41E-03 |
| positive regulation of cell motility | 3.00E-03 |
| protein deacylation | 3.03E-03 |
| toll-like receptor TLR6:TLR2 signaling pathway | 3.03E-03 |
| toll-like receptor TLR1:TLR2 signaling pathway | 3.03E-03 |
| SREBP signalling | 3.63E-03 |
| negative regulation of cellular catabolic process | 3.19E-03 |
| mitochondrion organization | 3.19E-03 |
| positive regulation of cellular component movement | 3.22E-03 |
| interspecies interaction between organisms | 3.24E-03 |
| symbiosis, encompassing mutualism through parasitism | 3.24E-03 |
| Fas Signaling Pathway | 3.72E-03 |
| HIF-1-alpha transcription factor network | 3.72E-03 |
| homeostatic process | 3.29E-03 |
| cellular response to lipid | 3.29E-03 |
| multicellular organismal reproductive process | 3.29E-03 |
| negative regulation of intrinsic apoptotic signaling pathway | 3.29E-03 |
| positive regulation of peptidyl-serine phosphorylation | 3.29E-03 |
| negative regulation of protein processing | 3.29E-03 |
| toll-like receptor 9 signaling pathway | 3.29E-03 |
| positive regulation of type I interferon production | 3.29E-03 |
| Znf_hrmn_rcpt | 4.80E-03 |
| NUCLEAR_REC_DBD_2 | 4.80E-03 |
| NUCLEAR_REC_DBD_1 | 4.80E-03 |
| ZnF_C4 | 4.80E-03 |
| zf-C4 | 4.80E-03 |
| Nucl_hrmn_rcpt_lig_bd | 4.80E-03 |
| Nucl_hrmn_rcpt_lig-bd_core | 4.80E-03 |
| Hormone_recep | 4.80E-03 |
| HOLI | 4.80E-03 |
| p53-like_TF_DNA-bd | 4.80E-03 |
| Nucl_hormone_rcpt_ligand-bd | 4.80E-03 |
| Znf_NHR/GATA | 4.80E-03 |
| Znf_NHR/GATA | 4.80E-03 |
| P53_TAD | 4.80E-03 |
| Oestr_rcpt | 4.80E-03 |
| Oest_recep | 4.80E-03 |
| Andrgn_rcpt | 4.80E-03 |
| Myc-LZ | 4.80E-03 |
| p53_transactivation_domain | 4.80E-03 |
| Myc-LZ | 4.80E-03 |
| Androgen_recep | 4.80E-03 |
| positive regulation of locomotion | 3.34E-03 |
| glycerolipid metabolic process | 3.34E-03 |
| AGE/RAGE pathway | 3.82E-03 |
| TRIF-dependent toll-like receptor signaling pathway | 3.35E-03 |
| regulation of generation of precursor metabolites and energy | 3.35E-03 |
| p53-dependent G1/S DNA damage checkpoint | 3.84E-03 |
| Transcriptional activation of p53 responsive genes | 3.84E-03 |
| altered p53 signaling pathway | 3.84E-03 |
| E-cadherin signaling events | 3.84E-03 |
| estrogen signaling | 3.84E-03 |
| Transcriptional activation of cell cycle inhibitor p21 | 3.84E-03 |
| Kit Receptor Signaling Pathway | 3.84E-03 |
| Signaling events mediated by HDAC Class I | 3.84E-03 |
| DNA damage response | 3.84E-03 |
| superpathway of inositol phosphate compounds | 3.84E-03 |
| Skin Neoplasms | 4.57E-03 |
| Cytosolic sensors of pathogen-associated DNA | 3.91E-03 |
| Amphetamine addiction | 3.91E-03 |
| G1 to S cell cycle control | 3.91E-03 |
| Ras Pathway | 3.91E-03 |
| toll-like receptor 2 signaling pathway | 3.43E-03 |
| regulation of neural precursor cell proliferation | 3.52E-03 |
| multicellular organism reproduction | 3.56E-03 |
| spindle | 5.52E-03 |
| MyD88-independent toll-like receptor signaling pathway | 3.59E-03 |
| Adipocytokine signaling pathway | 4.13E-03 |
| nitric oxide metabolic process | 3.66E-03 |
| T cell differentiation in thymus | 3.66E-03 |
| oligodendrocyte differentiation | 3.66E-03 |
| PI3K Cascade | 4.21E-03 |
| TGFBR2 MSI Frameshift Mutants in Cancer | 4.21E-03 |
| SMAD4 MH2 Domain Mutants in Cancer | 4.21E-03 |
| Signaling by TGF-beta Receptor Complex | 4.21E-03 |
| Loss of Function of TGFBR1 in Cancer | 4.21E-03 |
| SMAD2/3 Phosphorylation Motif Mutants in Cancer | 4.21E-03 |
| Loss of Function of SMAD2/3 in Cancer | 4.21E-03 |
| TGFBR1 LBD Mutants in Cancer | 4.21E-03 |
| TGFBR2 Kinase Domain Mutants in Cancer | 4.21E-03 |
| TGFBR1 KD Mutants in Cancer | 4.21E-03 |
| SMAD2/3 MH2 Domain Mutants in Cancer | 4.21E-03 |
| Loss of Function of SMAD4 in Cancer | 4.21E-03 |
| Loss of Function of TGFBR2 in Cancer | 4.21E-03 |
| miRNAs involved in DDR | 4.21E-03 |
| Signaling by TGF-beta Receptor Complex in Cancer | 4.21E-03 |
| Signaling by NOTCH1 | 4.24E-03 |
| Signaling by NOTCH1 in Cancer | 4.24E-03 |
| Signaling by NOTCH1 t(7;9)(NOTCH1:M1580_K2555) Translocation Mutant | 4.24E-03 |
| Signaling by NOTCH1 HD Domain Mutants in Cancer | 4.24E-03 |
| FBXW7 Mutants and NOTCH1 in Cancer | 4.24E-03 |
| Signaling by NOTCH1 HD+PEST Domain Mutants in Cancer | 4.24E-03 |
| E2F transcription factor network | 4.24E-03 |
| Signaling by NOTCH1 PEST Domain Mutants in Cancer | 4.24E-03 |
| cellular response to alcohol | 3.74E-03 |
| odontogenesis of dentin-containing tooth | 3.74E-03 |
| somatodendritic compartment | 5.84E-03 |
| DNA biosynthetic process | 3.82E-03 |
| polysaccharide biosynthetic process | 3.82E-03 |
| skin development | 3.87E-03 |
| regionalization | 3.87E-03 |
| 3q26.3 | 1.05E-02 |
| 5q13.1 | 1.05E-02 |
| regulation of gliogenesis | 3.88E-03 |
| regulation of leukocyte apoptotic process | 3.88E-03 |
| cell fate specification | 3.88E-03 |
| Pertussis | 4.46E-03 |
| androgen receptor activity | 5.49E-03 |
| Krueppel-associated box domain binding | 5.49E-03 |
| regulation of autophagy | 3.95E-03 |
| toll-like receptor 3 signaling pathway | 3.95E-03 |
| MyD88-dependent toll-like receptor signaling pathway | 3.95E-03 |
| endomembrane system organization | 3.98E-03 |
| Leishmaniasis | 4.57E-03 |
| locomotion | 4.10E-03 |
| establishment of protein localization to mitochondrion | 4.10E-03 |
| DNA modification | 4.10E-03 |
| modification of morphology or physiology of other organism | 4.10E-03 |
| canonical Wnt signaling pathway involved in positive regulation of wound healing | 4.10E-03 |
| regulation of cell proliferation involved in outflow tract morphogenesis | 4.10E-03 |
| regulation of heparan sulfate proteoglycan biosynthetic process | 4.10E-03 |
| renal outer medulla development | 4.10E-03 |
| negative regulation of macromitophagy | 4.10E-03 |
| regulation of mitophagy | 4.10E-03 |
| somatic sex determination | 4.10E-03 |
| canonical Wnt signaling pathway involved in positive regulation of epithelial to mesenchymal transition | 4.10E-03 |
| lateral sprouting involved in mammary gland duct morphogenesis | 4.10E-03 |
| canonical Wnt signaling pathway involved in positive regulation of cardiac outflow tract cell proliferation | 4.10E-03 |
| epicardium-derived cardiac vascular smooth muscle cell differentiation | 4.10E-03 |
| regulation of macromitophagy | 4.10E-03 |
| positive regulation of epithelial cell proliferation involved in prostate gland development | 4.10E-03 |
| positive regulation of proteoglycan biosynthetic process | 4.10E-03 |
| metanephric cap development | 4.10E-03 |
| macromitophagy | 4.10E-03 |
| metanephric cap morphogenesis | 4.10E-03 |
| metanephric cap mesenchymal cell proliferation involved in metanephros development | 4.10E-03 |
| positive regulation of heparan sulfate proteoglycan biosynthetic process | 4.10E-03 |
| positive regulation of branching involved in lung morphogenesis | 4.10E-03 |
| negative regulation of mitophagy | 4.10E-03 |
| regulation of striated muscle cell differentiation | 4.22E-03 |
| Cytokine Signaling in Immune system | 4.89E-03 |
| Apoptosis Modulation and Signaling | 4.89E-03 |
| G alpha (12/13) signalling events | 4.89E-03 |
| pancreas development | 4.30E-03 |
| regulation of carbohydrate biosynthetic process | 4.30E-03 |
| Fc-gamma receptor signaling pathway involved in phagocytosis | 4.37E-03 |
| immune response-regulating cell surface receptor signaling pathway involved in phagocytosis | 4.37E-03 |
| protein localization to mitochondrion | 4.37E-03 |
| Neoplasm Recurrence, Local | 6.01E-03 |
| regulation of lipid transport | 4.45E-03 |
| Fc-gamma receptor signaling pathway | 4.45E-03 |
| positive regulation of protein modification process | 4.46E-03 |
| Phosphatidylinositol signaling system | 5.12E-03 |
| Validated targets of C-MYC transcriptional activation | 5.12E-03 |
| regulation of mitochondrion organization | 4.53E-03 |
| positive regulation of MAPK cascade | 4.55E-03 |
| Disease | 5.20E-03 |
| IRS-mediated signalling | 5.20E-03 |
| Integrin Signaling Pathway | 5.20E-03 |
| regulation of peptidyl-serine phosphorylation | 4.61E-03 |
| TRAF6 mediated induction of NFkB and MAP kinases upon TLR7/8 or 9 activation | 5.32E-03 |
| Pancreatic Neoplasms | 6.34E-03 |
| Pilomatrixoma | 6.34E-03 |
| Sarcoma family syndrome of Li and Fraumeni | 6.34E-03 |
| PILOMATRIXOMA | 6.34E-03 |
| ADRENOCORTICAL CARCINOMA, HEREDITARY; ADCC | 6.34E-03 |
| Pilomatrixoma | 6.34E-03 |
| LI-FRAUMENI SYNDROME 1; LFS1 | 6.34E-03 |
| Vulvar Lichen Sclerosus | 6.34E-03 |
| Hypospadias 1, X-Linked | 6.34E-03 |
| Craniopharyngioma | 6.34E-03 |
| Fibrous Dysplasia of Bone | 6.34E-03 |
| Nevus, Epidermal | 6.34E-03 |
| Urinary bladder cancer | 6.34E-03 |
| Adrenocortical Carcinoma, Hereditary | 6.34E-03 |
| Chromosome 17 deletion | 6.34E-03 |
| Aortic Valve, Calcification of | 6.34E-03 |
| HYPOSPADIAS 1, X-LINKED; HYSP1 | 6.34E-03 |
| Adenocarcinoma, Clear Cell | 6.34E-03 |
| Adaptive Immune System | 5.41E-03 |
| regulation of membrane potential | 4.74E-03 |
| Fc receptor mediated stimulatory signaling pathway | 4.78E-03 |
| metanephros development | 4.78E-03 |
| phosphatidylinositol biosynthetic process | 4.78E-03 |
| MyD88 dependent cascade initiated on endosome | 5.51E-03 |
| Toll Like Receptor 7/8 (TLR7/8) Cascade | 5.51E-03 |
| IRS-related events | 5.51E-03 |
| p75 NTR receptor-mediated signalling | 5.51E-03 |
| response to extracellular stimulus | 4.88E-03 |
| IRS-related events triggered by IGF1R | 5.61E-03 |
| Salmonella infection | 5.61E-03 |
| response to cAMP | 4.96E-03 |
| nucleocytoplasmic transport | 5.01E-03 |
| estrogen response element binding | 7.09E-03 |
| androgen binding | 7.09E-03 |
| regulation of mitotic cell cycle | 5.07E-03 |
| cellular lipid metabolic process | 5.09E-03 |
| 14q32.32 | 1.39E-02 |
| 8q24.21 | 1.39E-02 |
| nuclear transport | 5.16E-03 |
| regulation of cellular response to stress | 5.19E-03 |
| response to estradiol | 5.19E-03 |
| hormone-mediated signaling pathway | 5.19E-03 |
| negative regulation of extrinsic apoptotic signaling pathway | 5.19E-03 |
| IGF1R signaling cascade | 5.94E-03 |
| Signaling by Type 1 Insulin-like Growth Factor 1 Receptor (IGF1R) | 5.94E-03 |
| Toll Like Receptor 9 (TLR9) Cascade | 5.94E-03 |
| Genes related to Wnt-mediated signal transduction | 5.94E-03 |
| chemical homeostasis | 5.23E-03 |
| Insulin receptor signalling cascade | 6.06E-03 |
| response to inorganic substance | 5.33E-03 |
| protein heterodimerization activity | 7.50E-03 |
| regulation of embryonic development | 5.47E-03 |
| myotube differentiation | 5.52E-03 |
| ureteric bud development | 5.52E-03 |
| mesonephric tubule development | 5.52E-03 |
| mesonephric epithelium development | 5.52E-03 |
| cellular response to hypoxia | 5.52E-03 |
| oligodendrocyte apoptotic process | 5.52E-03 |
| regulation of centriole-centriole cohesion | 5.52E-03 |
| positive regulation of determination of dorsal identity | 5.52E-03 |
| negative regulation of heart induction | 5.52E-03 |
| cardioblast cell fate commitment | 5.52E-03 |
| regulation of cardioblast cell fate specification | 5.52E-03 |
| negative regulation of calcidiol 1-monooxygenase activity | 5.52E-03 |
| embryonic skeletal limb joint morphogenesis | 5.52E-03 |
| canonical Wnt signaling pathway involved in positive regulation of apoptotic process | 5.52E-03 |
| positive regulation of cellular respiration | 5.52E-03 |
| positive regulation of mitochondrial membrane potential | 5.52E-03 |
| negative regulation of cardiac cell fate specification | 5.52E-03 |
| negative regulation of helicase activity | 5.52E-03 |
| glial cell fate determination | 5.52E-03 |
| negative regulation of cardioblast cell fate specification | 5.52E-03 |
| foregut regionalization | 5.52E-03 |
| cardioblast cell fate specification | 5.52E-03 |
| trachea formation | 5.52E-03 |
| oviduct development | 5.52E-03 |
| renal inner medulla development | 5.52E-03 |
| lung induction | 5.52E-03 |
| positive regulation of ATP biosynthetic process | 5.52E-03 |
| negative regulation of heart induction by canonical Wnt signaling pathway | 5.52E-03 |
| prostate epithelial cord elongation | 5.52E-03 |
| ventricular compact myocardium morphogenesis | 5.52E-03 |
| tertiary branching involved in mammary gland duct morphogenesis | 5.52E-03 |
| limb joint morphogenesis | 5.52E-03 |
| lung field specification | 5.52E-03 |
| positive regulation of sulfur metabolic process | 5.52E-03 |
| positive regulation of hyaluronan biosynthetic process | 5.52E-03 |
| positive regulation of integrin biosynthetic process | 5.52E-03 |
| toll-like receptor 4 signaling pathway | 5.55E-03 |
| Xq12 | 1.50E-02 |
| 1p34 | 1.50E-02 |
| 3p21 | 1.50E-02 |
| 4q24 | 1.50E-02 |
| 6q25.1 | 1.50E-02 |
| cellular response to decreased oxygen levels | 5.64E-03 |
| negative regulation of neurogenesis | 5.64E-03 |
| regulation of type I interferon production | 5.71E-03 |
| mesonephros development | 5.71E-03 |
| myelination | 5.71E-03 |
| ErbB-3 class receptor binding | 8.09E-03 |
| phosphatidylinositol-4,5-bisphosphate 3-kinase activity | 8.09E-03 |
| 1-phosphatidylinositol-3-kinase regulator activity | 8.09E-03 |
| estrogen receptor activity | 8.09E-03 |
| histone deacetylase regulator activity | 8.09E-03 |
| movement of cell or subcellular component | 5.81E-03 |
| AKT-mediated inactivation of FOXO1A | 6.70E-03 |
| nuclear chromosome part | 9.14E-03 |
| execution phase of apoptosis | 5.98E-03 |
| ensheathment of neurons | 5.98E-03 |
| axon ensheathment | 5.98E-03 |
| type I interferon production | 5.98E-03 |
| catalytic complex | 9.31E-03 |
| B cell differentiation | 6.08E-03 |
| cell-cell junction | 9.35E-03 |
| beta-catenin-TCF7L2 complex | 9.35E-03 |
| chromatin | 9.35E-03 |
| Toll Like Receptor 3 (TLR3) Cascade | 6.97E-03 |
| MyD88-independent cascade | 6.97E-03 |
| Beta-catenin | 8.88E-03 |
| P53 | 8.88E-03 |
| P53_tetramer | 8.88E-03 |
| PI3K_p85-bd | 8.88E-03 |
| p53_tumour_suppressor | 8.88E-03 |
| p53_deltaN | 8.88E-03 |
| p53_DNA-bd | 8.88E-03 |
| Serine/threonine_Kinase_Rac | 8.88E-03 |
| p53_tetrameristn | 8.88E-03 |
| PI3K_p85B | 8.88E-03 |
| p53_tetrameristn | 8.88E-03 |
| P53 | 8.88E-03 |
| PI3K_p85B | 8.88E-03 |
