# Supplementary material for: ECGene: A Literature‐Based Knowledgebase of Endometrial Cancer Genes
Source: Hum Mutat. 2016 Jan 13;37(4):337–43. doi: 10.1002/humu.22950 (PMC5066700; doi:10.1002/humu.22950)
Supplement: Supplementary file 10 — Supp. Table S9. The enriched functional terms of 43 EC‐implicated genes with 100 or more positively co‐expressed lncRNAs. [file HUMU-37-337-s002.docx]

| **Supp. Table S9. The enriched functional terms of 43 EC-implicated genes with 100 or more positively co-expressed lncRNAs.** | |
| --- | --- |
|  |  |
| **Name** | **q-value FDR B&H** |
| Pathways in cancer | 1.23E-10 |
| nucleoplasm | 1.97E-10 |
| cell cycle | 1.83E-09 |
| regulation of cell cycle | 2.71E-09 |
| chromosome organization | 2.71E-09 |
| apoptotic process | 4.56E-09 |
| programmed cell death | 4.85E-09 |
| single-organism organelle organization | 7.28E-09 |
| regulation of intracellular signal transduction | 9.30E-09 |
| positive regulation of signaling | 1.14E-08 |
| response to abiotic stimulus | 1.14E-08 |
| regulation of apoptotic process | 1.14E-08 |
| regulation of programmed cell death | 1.27E-08 |
| regulation of organelle organization | 1.30E-08 |
| regulation of cell death | 2.62E-08 |
| cell proliferation | 2.62E-08 |
| positive regulation of signal transduction | 2.62E-08 |
| negative regulation of cell death | 2.62E-08 |
| cellular response to stress | 2.62E-08 |
| response to UV | 3.35E-08 |
| Integrated Breast Cancer Pathway | 4.90E-08 |
| p53 pathway feedback loops 2 | 5.42E-08 |
| positive regulation of cell communication | 5.85E-08 |
| cell cycle process | 5.85E-08 |
| positive regulation of response to stimulus | 6.09E-08 |
| Signaling Pathways in Glioblastoma | 7.00E-08 |
| regulation of cell proliferation | 1.04E-07 |
| chromosome | 1.12E-07 |
| positive regulation of protein metabolic process | 1.27E-07 |
| regulation of chromosome organization | 1.32E-07 |
| regulation of protein modification process | 1.46E-07 |
| regulation of developmental process | 1.56E-07 |
| Integrated Pancreatic Cancer Pathway | 1.90E-07 |
| negative regulation of macromolecule metabolic process | 1.92E-07 |
| phosphorylation | 2.30E-07 |
| Colorectal cancer | 2.81E-07 |
| Cell cycle | 2.86E-07 |
| response to radiation | 3.83E-07 |
| cellular response to DNA damage stimulus | 3.95E-07 |
| Prostate Cancer | 4.26E-07 |
| regulation of cellular protein metabolic process | 4.89E-07 |
| negative regulation of apoptotic process | 5.33E-07 |
| positive regulation of intracellular signal transduction | 5.52E-07 |
| negative regulation of programmed cell death | 6.19E-07 |
| negative regulation of metabolic process | 6.47E-07 |
| Scrib-APC-beta-catenin complex | 8.48E-07 |
| regulation of cell cycle process | 8.82E-07 |
| regulation of DNA metabolic process | 9.15E-07 |
| negative regulation of cellular metabolic process | 1.05E-06 |
| Cell cycle | 1.06E-06 |
| cell development | 1.25E-06 |
| negative regulation of nucleobase-containing compound metabolic process | 1.25E-06 |
| regulation of mitotic cell cycle | 1.42E-06 |
| cytoskeleton organization | 1.51E-06 |
| negative regulation of nitrogen compound metabolic process | 1.52E-06 |
| generation of neurons | 1.53E-06 |
| positive regulation of cellular protein metabolic process | 1.53E-06 |
| cellular response to oxygen-containing compound | 1.53E-06 |
| Cell Cycle: G2/M Checkpoint | 1.69E-06 |
| enzyme binding | 1.80E-06 |
| structure-specific DNA binding | 1.80E-06 |
| negative regulation of DNA metabolic process | 2.06E-06 |
| Familial cancer of breast | 2.35E-06 |
| BREAST CANCER | 2.35E-06 |
| response to light stimulus | 2.54E-06 |
| neurogenesis | 3.04E-06 |
| neuron differentiation | 3.47E-06 |
| positive regulation of developmental process | 4.16E-06 |
| PI3/4_kinase_CS | 4.17E-06 |
| PI3Kc | 4.17E-06 |
| PI3/4_kinase_cat | 4.17E-06 |
| PI3_4_KINASE_3 | 4.17E-06 |
| PI3_4_KINASE_1 | 4.17E-06 |
| PI3_4_KINASE_2 | 4.17E-06 |
| PI3/4_kinase_cat | 4.17E-06 |
| PI3_PI4_kinase | 4.17E-06 |
| p53 pathway | 4.19E-06 |
| MicroRNAs in cancer | 4.58E-06 |
| signal transduction by p53 class mediator | 4.79E-06 |
| cell cycle checkpoint | 4.79E-06 |
| peptidyl-amino acid modification | 5.38E-06 |
| regulation of cellular component organization | 5.45E-06 |
| mitotic cell cycle phase transition | 5.45E-06 |
| response to oxygen-containing compound | 5.45E-06 |
| negative regulation of cell cycle | 5.50E-06 |
| meiotic cell cycle process | 5.59E-06 |
| positive regulation of gene expression | 6.09E-06 |
| cell cycle phase transition | 6.09E-06 |
| negative regulation of cell cycle process | 6.49E-06 |
| hematopoietic or lymphoid organ development | 6.49E-06 |
| mitotic cell cycle checkpoint | 6.59E-06 |
| nucleolus | 7.27E-06 |
| chromosomal part | 7.27E-06 |
| chromosome, telomeric region | 7.27E-06 |
| chromosomal region | 7.27E-06 |
| nuclear chromosome | 7.30E-06 |
| nuclear chromosome, telomeric region | 7.30E-06 |
| cellular response to nitrogen compound | 7.68E-06 |
| macromolecular complex assembly | 7.73E-06 |
| RNA polymerase II transcription factor binding | 8.88E-06 |
| protein complex assembly | 9.56E-06 |
| positive regulation of cell differentiation | 9.56E-06 |
| protein complex biogenesis | 9.56E-06 |
| Integrated Cancer pathway | 9.64E-06 |
| response to nitrogen compound | 9.90E-06 |
| immune system development | 9.90E-06 |
| cellular response to organic substance | 9.93E-06 |
| positive regulation of molecular function | 9.93E-06 |
| regulation of cell differentiation | 1.00E-05 |
| DNA metabolic process | 1.02E-05 |
| regulation of DNA replication | 1.17E-05 |
| glial cell proliferation | 1.17E-05 |
| transferase complex | 1.18E-05 |
| negative regulation of nuclear division | 1.27E-05 |
| positive regulation of nucleobase-containing compound metabolic process | 1.39E-05 |
| negative regulation of mitotic cell cycle phase transition | 1.41E-05 |
| positive regulation of protein modification process | 1.60E-05 |
| negative regulation of cell cycle phase transition | 1.62E-05 |
| transmembrane receptor protein tyrosine kinase signaling pathway | 1.73E-05 |
| positive regulation of nitrogen compound metabolic process | 1.79E-05 |
| cardiovascular system development | 1.91E-05 |
| circulatory system development | 1.91E-05 |
| nuclear chromosome part | 1.96E-05 |
| cellular response to peptide hormone stimulus | 1.98E-05 |
| p53 pathway | 2.14E-05 |
| telomere maintenance | 2.15E-05 |
| negative regulation of cellular macromolecule biosynthetic process | 2.25E-05 |
| telomere organization | 2.25E-05 |
| Ovarian Neoplasms | 2.28E-05 |
| Carcinoma, Hepatocellular | 2.28E-05 |
| mitotic cell cycle | 2.33E-05 |
| regulation of protein deacetylation | 2.33E-05 |
| hemopoiesis | 2.33E-05 |
| cellular response to peptide | 2.35E-05 |
| colorectal cancer | 2.55E-05 |
| Delta-Notch Signaling Pathway | 2.77E-05 |
| Viral carcinogenesis | 2.77E-05 |
| Progesterone-mediated oocyte maturation | 2.77E-05 |
| Prostate cancer | 3.21E-05 |
| nucleoplasm part | 3.23E-05 |
| catalytic complex | 3.39E-05 |
| DNA damage response (only ATM dependent) | 3.48E-05 |
| Trefoil Factors Initiate Mucosal Healing | 3.54E-05 |
| cellular response to abiotic stimulus | 3.55E-05 |
| negative regulation of macromolecule biosynthetic process | 3.55E-05 |
| Angiogenesis | 3.65E-05 |
| regulation of cytoskeleton organization | 3.67E-05 |
| meiosis I | 3.86E-05 |
| Carcinoma, Squamous Cell | 4.03E-05 |
| negative regulation of cellular component organization | 4.13E-05 |
| Endometrial cancer | 4.17E-05 |
| histone modification | 4.29E-05 |
| regulation of cell division | 4.29E-05 |
| positive regulation of cell proliferation | 4.43E-05 |
| MAPK cascade | 4.52E-05 |
| response to UV-C | 4.52E-05 |
| microtubule cytoskeleton organization | 4.52E-05 |
| covalent chromatin modification | 4.55E-05 |
| negative regulation of DNA replication | 4.55E-05 |
| Hypoxia and p53 in the Cardiovascular system | 4.56E-05 |
| developmental process involved in reproduction | 4.95E-05 |
| negative regulation of cellular biosynthetic process | 5.06E-05 |
| ErbB signaling pathway | 5.10E-05 |
| COLORECTAL CANCER; CRC | 5.19E-05 |
| positive regulation of neurogenesis | 5.53E-05 |
| regulation of mitotic cell cycle phase transition | 5.54E-05 |
| positive regulation of macromolecule biosynthetic process | 5.54E-05 |
| enzyme linked receptor protein signaling pathway | 5.54E-05 |
| protein complex binding | 5.72E-05 |
| oocyte development | 5.78E-05 |
| negative regulation of biosynthetic process | 5.80E-05 |
| multicellular organism growth | 6.18E-05 |
| cellular component morphogenesis | 6.18E-05 |
| Lung Neoplasms | 6.21E-05 |
| intrinsic apoptotic signaling pathway | 6.23E-05 |
| central nervous system development | 6.24E-05 |
| regulation of cell cycle phase transition | 6.26E-05 |
| regulation of phosphorylation | 6.26E-05 |
| protein modification by small protein conjugation | 6.26E-05 |
| chromosome segregation | 6.26E-05 |
| protein phosphorylation | 6.27E-05 |
| signal transduction by protein phosphorylation | 6.27E-05 |
| mitotic spindle assembly checkpoint | 6.27E-05 |
| positive regulation of mitotic cell cycle | 6.27E-05 |
| regulation of multicellular organismal development | 6.33E-05 |
| chromatin modification | 6.46E-05 |
| oocyte differentiation | 6.61E-05 |
| spindle assembly checkpoint | 6.61E-05 |
| negative regulation of mitotic metaphase/anaphase transition | 6.61E-05 |
| protein-DNA complex | 6.66E-05 |
| macromolecule catabolic process | 6.73E-05 |
| reproductive process | 6.85E-05 |
| negative regulation of metaphase/anaphase transition of cell cycle | 7.07E-05 |
| innate immune response | 7.12E-05 |
| cell migration | 7.13E-05 |
| embryo development | 7.21E-05 |
| positive regulation of microtubule polymerization | 7.46E-05 |
| positive regulation of RNA metabolic process | 7.46E-05 |
| MEDULLOBLASTOMA; MDB | 7.82E-05 |
| microtubule-based process | 7.84E-05 |
| mitotic spindle checkpoint | 7.98E-05 |
| anatomical structure homeostasis | 8.37E-05 |
| macromolecular complex subunit organization | 8.43E-05 |
| blood vessel development | 8.43E-05 |
| Adenoma | 8.51E-05 |
| HEPATOCELLULAR CARCINOMA | 8.51E-05 |
| Hepatocellular carcinoma | 8.51E-05 |
| DNA Repair | 9.59E-05 |
| protein kinase binding | 9.62E-05 |
| organ morphogenesis | 9.83E-05 |
| positive regulation of cellular biosynthetic process | 1.01E-04 |
| DNA damage response, signal transduction by p53 class mediator | 1.04E-04 |
| response to peptide hormone | 1.05E-04 |
| phosphatidylinositol-3-phosphate biosynthetic process | 1.05E-04 |
| regulation of telomere maintenance | 1.05E-04 |
| meiotic cell cycle | 1.06E-04 |
| HTLV-I infection | 1.06E-04 |
| Pancreatic cancer | 1.06E-04 |
| Renal cell carcinoma | 1.06E-04 |
| Regulation of Telomerase | 1.06E-04 |
| regulation of mitotic metaphase/anaphase transition | 1.11E-04 |
| peptidyl-lysine modification | 1.13E-04 |
| single organism reproductive process | 1.13E-04 |
| protein complex subunit organization | 1.13E-04 |
| Breast Neoplasms | 1.14E-04 |
| transcription factor complex | 1.15E-04 |
| microtubule cytoskeleton | 1.15E-04 |
| metaphase/anaphase transition of mitotic cell cycle | 1.15E-04 |
| regulation of metaphase/anaphase transition of cell cycle | 1.15E-04 |
| vasculature development | 1.15E-04 |
| positive regulation of biosynthetic process | 1.17E-04 |
| metaphase/anaphase transition of cell cycle | 1.22E-04 |
| response to organonitrogen compound | 1.26E-04 |
| negative regulation of mitotic nuclear division | 1.29E-04 |
| protein modification by small protein conjugation or removal | 1.29E-04 |
| positive regulation of catalytic activity | 1.30E-04 |
| positive regulation of organelle organization | 1.30E-04 |
| response to peptide | 1.30E-04 |
| cell morphogenesis | 1.35E-04 |
| reproductive structure development | 1.35E-04 |
| 1-phosphatidylinositol-3-kinase activity | 1.36E-04 |
| phosphatidylinositol-mediated signaling | 1.36E-04 |
| apoptotic signaling pathway | 1.36E-04 |
| inositol lipid-mediated signaling | 1.36E-04 |
| localization of cell | 1.36E-04 |
| cell motility | 1.36E-04 |
| leukocyte differentiation | 1.37E-04 |
| reproductive system development | 1.37E-04 |
| spindle checkpoint | 1.37E-04 |
| ErbB4 signaling events | 1.40E-04 |
| CDC42 signaling events | 1.43E-04 |
| Signaling events mediated by Hepatocyte Growth Factor Receptor (c-Met) | 1.45E-04 |
| Signaling of Hepatocyte Growth Factor Receptor | 1.45E-04 |
| signal transduction in response to DNA damage | 1.47E-04 |
| gliogenesis | 1.47E-04 |
| Chronic myeloid leukemia | 1.50E-04 |
| positive regulation of microtubule polymerization or depolymerization | 1.52E-04 |
| cellular response to organonitrogen compound | 1.54E-04 |
| cellular response to radiation | 1.62E-04 |
| Urinary Bladder Neoplasms | 1.62E-04 |
| GASTRIC CANCER | 1.62E-04 |
| Wilms Tumor | 1.62E-04 |
| mitotic cell cycle process | 1.66E-04 |
| negative regulation of cell proliferation | 1.66E-04 |
| kinase binding | 1.68E-04 |
| Cellular responses to stress | 1.68E-04 |
| regulation of phosphate metabolic process | 1.68E-04 |
| response to ionizing radiation | 1.71E-04 |
| positive regulation of cell death | 1.73E-04 |
| positive regulation of phosphorylation | 1.73E-04 |
| ARM-type_fold | 1.75E-04 |
| regulation of immune system process | 1.78E-04 |
| regulation of phosphorus metabolic process | 1.78E-04 |
| neuron development | 1.79E-04 |
| anatomical structure formation involved in morphogenesis | 1.79E-04 |
| homeostatic process | 1.79E-04 |
| brain development | 1.81E-04 |
| protein ubiquitination | 1.81E-04 |
| positive regulation of transcription, DNA-templated | 1.86E-04 |
| positive regulation of cell development | 1.90E-04 |
| double-strand break repair | 1.92E-04 |
| epidermal growth factor receptor signaling pathway | 1.93E-04 |
| positive regulation of determination of dorsal identity | 1.94E-04 |
| telomere maintenance via telomere shortening | 1.94E-04 |
| nucleotide-excision repair, DNA incision, 3'-to lesion | 1.94E-04 |
| blood vessel morphogenesis | 2.08E-04 |
| ERBB signaling pathway | 2.12E-04 |
| Signaling of Hepatocyte Growth Factor Receptor | 2.13E-04 |
| Medulloblastoma | 2.13E-04 |
| regulation of protein stability | 2.14E-04 |
| protein deacetylation | 2.23E-04 |
| chromatin organization | 2.23E-04 |
| Fc receptor signaling pathway | 2.23E-04 |
| protein kinase B signaling | 2.32E-04 |
| aging | 2.32E-04 |
| cellular macromolecule catabolic process | 2.40E-04 |
| positive regulation of RNA biosynthetic process | 2.40E-04 |
| Glucocorticoid receptor regulatory network | 2.44E-04 |
| FOXM1 transcription factor network | 2.44E-04 |
| Apoptotic cleavage of cellular proteins | 2.44E-04 |
| protein deacylation | 2.56E-04 |
| peroxisome proliferator activated receptor binding | 2.57E-04 |
| Neoplasms | 2.66E-04 |
| Regulation of Actin Cytoskeleton | 2.78E-04 |
| negative regulation of organelle organization | 2.86E-04 |
| neuron projection development | 2.93E-04 |
| oogenesis | 2.97E-04 |
| regulation of proteolysis | 2.99E-04 |
| DNA damage checkpoint | 2.99E-04 |
| ErbB signaling pathway | 3.06E-04 |
| Androgen receptor signaling pathway | 3.06E-04 |
| regulation of nuclear division | 3.07E-04 |
| lamellipodium | 3.13E-04 |
| microtubule organizing center | 3.19E-04 |
| cellular response to hormone stimulus | 3.20E-04 |
| regulation of microtubule polymerization | 3.29E-04 |
| TWEAK Signaling Pathway | 3.29E-04 |
| nuclear division | 3.35E-04 |
| response to hypoxia | 3.41E-04 |
| N-terminal peptidyl-lysine acetylation | 3.46E-04 |
| fungiform papilla morphogenesis | 3.46E-04 |
| fungiform papilla formation | 3.46E-04 |
| negative regulation of developmental process | 3.51E-04 |
| positive regulation of phosphorus metabolic process | 3.53E-04 |
| positive regulation of phosphate metabolic process | 3.53E-04 |
| response to X-ray | 3.55E-04 |
| response to lipid | 3.56E-04 |
| response to decreased oxygen levels | 3.56E-04 |
| epithelium development | 3.59E-04 |
| DNA integrity checkpoint | 3.67E-04 |
| multi-organism cellular process | 3.74E-04 |
| Carcinoma, Adenoid Cystic | 3.82E-04 |
| Multi-step Regulation of Transcription by Pitx2 | 3.84E-04 |
| spindle | 3.85E-04 |
| regulation of DNA damage response, signal transduction by p53 class mediator | 3.86E-04 |
| cellular component disassembly involved in execution phase of apoptosis | 4.06E-04 |
| regulation of cellular response to stress | 4.22E-04 |
| regulation of gliogenesis | 4.22E-04 |
| phosphatidylinositol 3-kinase activity | 4.35E-04 |
| double-stranded DNA binding | 4.35E-04 |
| transcription factor binding | 4.35E-04 |
| FoxO family signaling | 4.50E-04 |
| response to oxygen levels | 4.58E-04 |
| cell aging | 4.58E-04 |
| organelle fission | 4.58E-04 |
| regulation of protein processing | 4.58E-04 |
| neurotrophin TRK receptor signaling pathway | 4.58E-04 |
| positive regulation of cellular component organization | 4.58E-04 |
| growth | 4.75E-04 |
| regulation of MAPK cascade | 4.75E-04 |
| response to drug | 4.78E-04 |
| neurotrophin signaling pathway | 4.78E-04 |
| NOTCH1 Intracellular Domain Regulates Transcription | 4.79E-04 |
| gland development | 4.96E-04 |
| regulation of oligodendrocyte differentiation | 4.96E-04 |
| positive regulation of proteolysis | 5.04E-04 |
| fungiform papilla development | 5.12E-04 |
| regulation of determination of dorsal identity | 5.12E-04 |
| Aberrant Crypt Foci | 5.26E-04 |
| regulation of anatomical structure morphogenesis | 5.29E-04 |
| microtubule polymerization | 5.36E-04 |
| phosphatidylinositol kinase activity | 5.36E-04 |
| DNA repair | 5.44E-04 |
| G2/M transition of mitotic cell cycle | 5.66E-04 |
| peptidyl-serine phosphorylation | 5.75E-04 |
| regulation of DNA-dependent DNA replication | 5.75E-04 |
| hair follicle development | 5.75E-04 |
| molting cycle process | 5.75E-04 |
| hair cycle process | 5.75E-04 |
| cell division | 5.81E-04 |
| cell cycle G2/M phase transition | 5.81E-04 |
| positive regulation of apoptotic process | 5.90E-04 |
| immune response | 5.92E-04 |
| insulin receptor signaling pathway | 6.03E-04 |
| Fc-epsilon receptor signaling pathway | 6.03E-04 |
| phosphatidylinositol phosphorylation | 6.09E-04 |
| regulation of chromosome segregation | 6.09E-04 |
| response to endogenous stimulus | 6.14E-04 |
| IL-3 Signaling Pathway | 6.15E-04 |
| Telomeres, Telomerase, Cellular Aging, and Immortality | 6.15E-04 |
| PTEN dependent cell cycle arrest and apoptosis | 6.15E-04 |
| cell projection organization | 6.29E-04 |
| positive regulation of programmed cell death | 6.30E-04 |
| lymphocyte differentiation | 6.30E-04 |
| fibroblast growth factor receptor signaling pathway | 6.50E-04 |
| gamete generation | 6.56E-04 |
| Apoptotic execution phase | 6.56E-04 |
| meiotic nuclear division | 6.62E-04 |
| mitotic DNA damage checkpoint | 6.66E-04 |
| canonical Wnt signaling pathway involved in negative regulation of apoptotic process | 6.93E-04 |
| hair follicle placode formation | 6.93E-04 |
| regulation of response to stress | 7.02E-04 |
| reproduction | 7.11E-04 |
| peptidyl-serine modification | 7.16E-04 |
| DNA replication | 7.24E-04 |
| positive regulation of cell migration | 7.24E-04 |
| movement of cell or subcellular component | 7.40E-04 |
| sister chromatid cohesion | 7.40E-04 |
| posttranscriptional regulation of gene expression | 7.47E-04 |
| positive regulation of protein processing | 7.50E-04 |
| regulation of protein phosphorylation | 7.64E-04 |
| mitotic DNA integrity checkpoint | 7.74E-04 |
| single-stranded DNA binding | 7.75E-04 |
| positive regulation of cell motility | 7.84E-04 |
| DNA repair complex | 7.89E-04 |
| lung development | 7.99E-04 |
| ATM Signaling Pathway | 8.15E-04 |
| Constitutive Signaling by NOTCH1 HD+PEST Domain Mutants | 8.15E-04 |
| VEGF signaling pathway | 8.15E-04 |
| HIV-I Nef: negative effector of Fas and TNF | 8.15E-04 |
| Notch signaling pathway | 8.15E-04 |
| Constitutive Signaling by NOTCH1 PEST Domain Mutants | 8.15E-04 |
| Mechanism of Gene Regulation by Peroxisome Proliferators via PPARa(alpha) | 8.15E-04 |
| Wnt Signaling Pathway NetPath | 8.15E-04 |
| Role of BRCA1, BRCA2 and ATR in Cancer Susceptibility | 8.30E-04 |
| protein catabolic process | 8.40E-04 |
| lipid phosphorylation | 8.44E-04 |
| heart development | 8.64E-04 |
| respiratory tube development | 8.65E-04 |
| cytoskeletal part | 8.65E-04 |
| cytoskeleton | 8.72E-04 |
| positive regulation of cellular component movement | 8.91E-04 |
| cell morphogenesis involved in neuron differentiation | 8.91E-04 |
| reciprocal DNA recombination | 8.93E-04 |
| reciprocal meiotic recombination | 8.93E-04 |
| muscle cell differentiation | 8.93E-04 |
| Schwann cell proliferation | 8.93E-04 |
| nucleotide-excision repair, DNA incision | 8.93E-04 |
| positive regulation of protein kinase activity | 8.95E-04 |
| Carcinoma of colon | 9.07E-04 |
| Cecal Neoplasms | 9.07E-04 |
| Penile Neoplasms | 9.07E-04 |
| cellular response to fibroblast growth factor stimulus | 9.20E-04 |
| chordate embryonic development | 9.24E-04 |
| cellular response to lipid | 9.24E-04 |
| cell morphogenesis involved in differentiation | 9.25E-04 |
| Role of ERBB2 in Signal Transduction and Oncology | 9.42E-04 |
| VEGF signaling pathway | 9.42E-04 |
| neuron projection morphogenesis | 9.45E-04 |
| Cell Cycle | 9.46E-04 |
| positive regulation of locomotion | 9.60E-04 |
| response to fibroblast growth factor | 9.60E-04 |
| regulation of myeloid leukocyte differentiation | 9.60E-04 |
| regulation of cellular component biogenesis | 9.60E-04 |
| defense response | 9.60E-04 |
| immune response-regulating signaling pathway | 9.69E-04 |
| regulation of protein ubiquitination | 9.72E-04 |
| embryo development ending in birth or egg hatching | 9.74E-04 |
| negative regulation of transcription from RNA polymerase II promoter | 9.74E-04 |
| hair cycle | 9.74E-04 |
| molting cycle | 9.74E-04 |
| positive regulation of multicellular organismal process | 9.75E-04 |
| cellular response to growth factor stimulus | 9.75E-04 |
| locomotion | 9.75E-04 |
| Angiogenesis | 9.90E-04 |
| G alpha 12 Pathway | 9.90E-04 |
| Double-Strand Break Repair | 9.90E-04 |
| Signaling events mediated by VEGFR1 and VEGFR2 | 9.90E-04 |
| regulation of microtubule cytoskeleton organization | 9.96E-04 |
| Epstein-Barr virus infection | 1.02E-03 |
| IL-4 signaling Pathway | 1.02E-03 |
| execution phase of apoptosis | 1.03E-03 |
| female gamete generation | 1.03E-03 |
| Glioma | 1.04E-03 |
| CXCR4 Signaling Pathway | 1.04E-03 |
| Erk and PI-3 Kinase Are Necessary for Collagen Binding in Corneal Epithelia | 1.04E-03 |
| Focal adhesion | 1.05E-03 |
| myeloid cell differentiation | 1.05E-03 |
| intrinsic apoptotic signaling pathway in response to DNA damage | 1.05E-03 |
| canonical Wnt signaling pathway | 1.07E-03 |
| cellular response to UV | 1.07E-03 |
| response to hormone | 1.07E-03 |
| protein processing | 1.07E-03 |
| regulation of sarcomere organization | 1.07E-03 |
| regulation of miRNA metabolic process | 1.07E-03 |
| regulation of telomere maintenance via telomerase | 1.07E-03 |
| tongue morphogenesis | 1.07E-03 |
| positive regulation of kinase activity | 1.09E-03 |
| odontogenesis | 1.09E-03 |
| response to growth factor | 1.10E-03 |
| intrinsic apoptotic signaling pathway in response to DNA damage by p53 class mediator | 1.12E-03 |
| regulation of signal transduction by p53 class mediator | 1.12E-03 |
| double-stranded telomeric DNA binding | 1.14E-03 |
| histone deacetylase regulator activity | 1.14E-03 |
| Kinase-like_dom | 1.15E-03 |
| TRAIL signaling pathway | 1.15E-03 |
| regulation of transferase activity | 1.16E-03 |
| germ cell development | 1.16E-03 |
| immune response-regulating cell surface receptor signaling pathway | 1.17E-03 |
| Developmental Biology | 1.18E-03 |
| Ras Pathway | 1.18E-03 |
| regulation of sequence-specific DNA binding transcription factor activity | 1.18E-03 |
| positive regulation of gliogenesis | 1.18E-03 |
| positive regulation of protein phosphorylation | 1.20E-03 |
| IL-5 Signaling Pathway | 1.22E-03 |
| Hypoxia response via HIF activation | 1.22E-03 |
| Fanconi Anemia pathway | 1.22E-03 |
| respiratory system development | 1.22E-03 |
| Direct p53 effectors | 1.23E-03 |
| Fc epsilon RI signaling pathway | 1.23E-03 |
| Melanoma | 1.23E-03 |
| Phospholipids as signalling intermediaries | 1.23E-03 |
| Granule Cell Survival Pathway is a specific case of more general PAC1 Receptor Pathway. | 1.23E-03 |
| Inactivation of Gsk3 by AKT causes accumulation of b-catenin in Alveolar Macrophages | 1.23E-03 |
| Ubiquitin mediated proteolysis | 1.23E-03 |
| Prolactin signaling pathway | 1.23E-03 |
| altered canonical Wnt signaling | 1.23E-03 |
| LRR FLII-interacting protein 1 (LRRFIP1) activates type I IFN production | 1.23E-03 |
| Signaling by NOTCH1 | 1.23E-03 |
| Signaling by NOTCH1 in Cancer | 1.23E-03 |
| Signaling by NOTCH1 t(7;9)(NOTCH1:M1580_K2555) Translocation Mutant | 1.23E-03 |
| FBXW7 Mutants and NOTCH1 in Cancer | 1.23E-03 |
| Signaling by NOTCH1 PEST Domain Mutants in Cancer | 1.23E-03 |
| Signaling by NOTCH1 HD Domain Mutants in Cancer | 1.23E-03 |
| Signaling by NOTCH1 HD+PEST Domain Mutants in Cancer | 1.23E-03 |
| protein maturation | 1.23E-03 |
| circadian regulation of gene expression | 1.24E-03 |
| response to gamma radiation | 1.24E-03 |
| Cell Cycle: G1/S Check Point | 1.24E-03 |
| Proteoglycans in cancer | 1.27E-03 |
| adherens junction | 1.27E-03 |
| Prolactin Signaling Pathway | 1.31E-03 |
| Thyroid cancer | 1.31E-03 |
| BARD1 signaling events | 1.31E-03 |
| VEGF, Hypoxia, and Angiogenesis | 1.31E-03 |
| Colorectal Neoplasms | 1.33E-03 |
| MISMATCH REPAIR CANCER SYNDROME | 1.33E-03 |
| Turcot syndrome | 1.33E-03 |
| positive regulation of cell cycle process | 1.33E-03 |
| myelin sheath abaxonal region | 1.35E-03 |
| cell cycle arrest | 1.36E-03 |
| RNA polymerase II activating transcription factor binding | 1.38E-03 |
| MutLalpha complex binding | 1.38E-03 |
| protein N-terminus binding | 1.38E-03 |
| ATP binding | 1.38E-03 |
| kinase activity | 1.38E-03 |
| negative regulation of gene expression | 1.39E-03 |
| regulation of mitotic nuclear division | 1.43E-03 |
| viral process | 1.44E-03 |
| Hepatitis B | 1.45E-03 |
| DNA recombination | 1.45E-03 |
| cellular response to ionizing radiation | 1.46E-03 |
| regulation of muscle cell differentiation | 1.46E-03 |
| cell activation | 1.46E-03 |
| Regulation of Wnt-mediated beta catenin signaling and target gene transcription | 1.54E-03 |
| regulation of protein kinase activity | 1.56E-03 |
| Signaling by ERBB4 | 1.56E-03 |
| regulation of catabolic process | 1.57E-03 |
| histone H3-K4 trimethylation | 1.58E-03 |
| negative regulation of telomere maintenance | 1.58E-03 |
| regulation of epithelial cell proliferation involved in prostate gland development | 1.58E-03 |
| cellular response to insulin stimulus | 1.59E-03 |
| adenyl ribonucleotide binding | 1.63E-03 |
| 1-phosphatidylinositol-4-phosphate 3-kinase activity | 1.63E-03 |
| regulation of transcription from RNA polymerase II promoter | 1.63E-03 |
| striated muscle cell differentiation | 1.64E-03 |
| anchoring junction | 1.66E-03 |
| stress-activated MAPK cascade | 1.67E-03 |
| histone H4 acetylation | 1.68E-03 |
| positive regulation of cell cycle | 1.69E-03 |
| Osteopontin-mediated events | 1.70E-03 |
| adenyl nucleotide binding | 1.72E-03 |
| Integrin Signaling Pathway | 1.73E-03 |
| positive regulation of MAPK cascade | 1.74E-03 |
| purine ribonucleoside triphosphate binding | 1.76E-03 |
| mismatch repair complex binding | 1.76E-03 |
| purine ribonucleoside binding | 1.76E-03 |
| purine nucleoside binding | 1.76E-03 |
| ribonucleoside binding | 1.76E-03 |
| regulation of protein localization | 1.76E-03 |
| regulation of microtubule-based process | 1.77E-03 |
| Hippo signaling pathway | 1.77E-03 |
| nucleoside binding | 1.79E-03 |
| stress-activated protein kinase signaling cascade | 1.80E-03 |
| regulation of neuron death | 1.80E-03 |
| Intracellular Signalling Through Adenosine Receptor A2a and Adenosine | 1.80E-03 |
| N-cadherin signaling events | 1.80E-03 |
| N-terminal protein amino acid acetylation | 1.86E-03 |
| replicative senescence | 1.86E-03 |
| negative regulation of DNA-dependent DNA replication | 1.86E-03 |
| epithelial cell proliferation involved in prostate gland development | 1.86E-03 |
| Insulin/IGF pathway-protein kinase B signaling cascade | 1.89E-03 |
| Angiotensin II mediated activation of JNK Pathway via Pyk2 dependent signaling | 1.89E-03 |
| Gastric cancer network 2 | 1.89E-03 |
| Intracellular Signalling Through Adenosine Receptor A2b and Adenosine | 1.89E-03 |
| Genes related to CD40 signaling | 1.89E-03 |
| axonogenesis | 1.90E-03 |
| Neoplasm of ovary | 1.91E-03 |
| Small cell lung cancer | 1.92E-03 |
| purine ribonucleotide binding | 1.93E-03 |
| regulation of protein binding | 1.94E-03 |
| FATC | 1.99E-03 |
| FAT | 1.99E-03 |
| PIK-rel_kinase_FAT | 1.99E-03 |
| FAT | 1.99E-03 |
| FATC | 1.99E-03 |
| PIK_FAT | 1.99E-03 |
| FATC | 1.99E-03 |
| negative regulation of cell differentiation | 1.99E-03 |
| ErbB2/ErbB3 signaling events | 2.01E-03 |
| FAS (CD95) signaling pathway | 2.01E-03 |
| histone deacetylation | 2.01E-03 |
| response to organic cyclic compound | 2.01E-03 |
| ribonucleotide binding | 2.02E-03 |
| purine nucleotide binding | 2.02E-03 |
| phosphotransferase activity, alcohol group as acceptor | 2.02E-03 |
| positive regulation of cytoskeleton organization | 2.03E-03 |
| Apoptosis | 2.04E-03 |
| regulation of neurogenesis | 2.04E-03 |
| regulation of microtubule polymerization or depolymerization | 2.10E-03 |
| establishment or maintenance of cell polarity | 2.12E-03 |
| peptidyl-lysine acetylation | 2.12E-03 |
| Adrenergic Pathway | 2.13E-03 |
| Agrin in Postsynaptic Differentiation | 2.13E-03 |
| UV protection | 2.15E-03 |
| determination of dorsal identity | 2.15E-03 |
| positive regulation of protein ubiquitination involved in ubiquitin-dependent protein catabolic process | 2.15E-03 |
| determination of dorsal/ventral asymmetry | 2.15E-03 |
| sexual reproduction | 2.16E-03 |
| Corticotropin-releasing hormone | 2.18E-03 |
| angiogenesis | 2.20E-03 |
| cell-cell adherens junction | 2.23E-03 |
| JUN kinase binding | 2.25E-03 |
| regulation of kinase activity | 2.25E-03 |
| negative regulation of transcription, DNA-templated | 2.26E-03 |
| regulation of glial cell differentiation | 2.26E-03 |
| beta-catenin destruction complex | 2.35E-03 |
| lymphocyte activation | 2.35E-03 |
| PI3K_ras-bd | 2.37E-03 |
| PI3K_rbd | 2.37E-03 |
| PI3K_rbd | 2.37E-03 |
| axon development | 2.37E-03 |
| muscle cell proliferation | 2.39E-03 |
| circadian rhythm | 2.39E-03 |
| Signaling events regulated by Ret tyrosine kinase | 2.40E-03 |
| Bladder cancer | 2.40E-03 |
| TSLP Signaling Pathway | 2.40E-03 |
| Integrin Signaling Pathway | 2.40E-03 |
| interspecies interaction between organisms | 2.44E-03 |
| symbiosis, encompassing mutualism through parasitism | 2.44E-03 |
| positive regulation of DNA damage response, signal transduction by p53 class mediator | 2.45E-03 |
| regulation of sister chromatid cohesion | 2.45E-03 |
| determination of adult lifespan | 2.45E-03 |
| axon | 2.45E-03 |
| cell projection | 2.45E-03 |
| telomere cap complex | 2.45E-03 |
| nuclear telomere cap complex | 2.45E-03 |
| cellular process involved in reproduction in multicellular organism | 2.48E-03 |
| cellular response to endogenous stimulus | 2.49E-03 |
| multicellular organismal reproductive process | 2.51E-03 |
| regulation of osteoclast differentiation | 2.52E-03 |
| positive regulation of transferase activity | 2.52E-03 |
| negative regulation of RNA biosynthetic process | 2.52E-03 |
| Fc Epsilon Receptor I Signaling in Mast Cells | 2.56E-03 |
| Aldosterone-regulated sodium reabsorption | 2.56E-03 |
| OVARIAN CANCER | 2.57E-03 |
| activating transcription factor binding | 2.59E-03 |
| positive regulation of chromosome organization | 2.62E-03 |
| damaged DNA binding | 2.63E-03 |
| transferase activity, transferring phosphorus-containing groups | 2.63E-03 |
| XY body | 2.64E-03 |
| Sin3-type complex | 2.64E-03 |
| Sin3 complex | 2.64E-03 |
| histone acetyltransferase activity | 2.65E-03 |
| regulation of immune response | 2.67E-03 |
| limb morphogenesis | 2.68E-03 |
| appendage morphogenesis | 2.68E-03 |
| IFN-gamma pathway | 2.73E-03 |
| regulation of canonical Wnt signaling pathway | 2.74E-03 |
| PI3K_C2 | 2.74E-03 |
| PI3K_C2 | 2.74E-03 |
| PI3K_C2 | 2.74E-03 |
| positive regulation of glycoprotein metabolic process | 2.76E-03 |
| positive regulation of glycoprotein biosynthetic process | 2.76E-03 |
| DNA damage response, signal transduction by p53 class mediator resulting in cell cycle arrest | 2.83E-03 |
| response to wounding | 2.84E-03 |
| multicellular organism reproduction | 2.90E-03 |
| protein acetylation | 2.90E-03 |
| signal transduction involved in mitotic G1 DNA damage checkpoint | 2.91E-03 |
| intracellular signal transduction involved in G1 DNA damage checkpoint | 2.91E-03 |
| intrinsic apoptotic signaling pathway by p53 class mediator | 2.91E-03 |
| IL-6 Signaling Pathway | 2.93E-03 |
| Transcriptional misregulation in cancer | 2.93E-03 |
| neuron death | 2.96E-03 |
| proteasomal protein catabolic process | 2.96E-03 |
| Stomach Neoplasms | 2.98E-03 |
| Lymphoma, T-Cell, Peripheral | 2.98E-03 |
| Colorectal Neoplasms, Hereditary Nonpolyposis | 2.98E-03 |
| Neoplasm of stomach | 2.98E-03 |
| signal transduction involved in mitotic cell cycle checkpoint | 2.99E-03 |
| signal transduction involved in DNA integrity checkpoint | 2.99E-03 |
| signal transduction involved in DNA damage checkpoint | 2.99E-03 |
| signal transduction involved in mitotic DNA damage checkpoint | 2.99E-03 |
| positive regulation of translation | 2.99E-03 |
| signal transduction involved in mitotic DNA integrity checkpoint | 2.99E-03 |
| tube development | 2.99E-03 |
| cell projection morphogenesis | 2.99E-03 |
| PInositide-3_kin_accessory_dom | 3.00E-03 |
| PI3Ka | 3.00E-03 |
| PI3Ka | 3.00E-03 |
| PI3Ka | 3.00E-03 |
| negative regulation of RNA metabolic process | 3.02E-03 |
| ectodermal placode formation | 3.02E-03 |
| ectodermal placode morphogenesis | 3.02E-03 |
| miRNA metabolic process | 3.02E-03 |
| ectodermal placode development | 3.02E-03 |
| regulation of gene expression, epigenetic | 3.05E-03 |
| signal transduction involved in cell cycle checkpoint | 3.05E-03 |
| G2/M DNA damage checkpoint | 3.08E-03 |
| response to insulin | 3.09E-03 |
| Melanogenesis | 3.09E-03 |
| Cellular Senescence | 3.09E-03 |
| FGF signaling pathway | 3.15E-03 |
| Apoptosis signaling pathway | 3.15E-03 |
| Focal Adhesion | 3.15E-03 |
| Signalling by NGF | 3.15E-03 |
| Interleukin-2 signaling | 3.15E-03 |
| cellular response to hydrogen peroxide | 3.17E-03 |
| cellular nitrogen compound catabolic process | 3.17E-03 |
| heterocycle catabolic process | 3.22E-03 |
| tissue development | 3.23E-03 |
| protein oligomerization | 3.23E-03 |
| double-strand break repair via homologous recombination | 3.27E-03 |
| transcription from RNA polymerase II promoter | 3.27E-03 |
| regulation of organ morphogenesis | 3.27E-03 |
| regulation of cellular catabolic process | 3.30E-03 |
| cell part morphogenesis | 3.33E-03 |
| positive regulation of signal transduction by p53 class mediator | 3.33E-03 |
| negative regulation of anoikis | 3.33E-03 |
| cellular response to gamma radiation | 3.33E-03 |
| microtubule polymerization or depolymerization | 3.34E-03 |
| recombinational repair | 3.34E-03 |
| regulation of nervous system development | 3.35E-03 |
| negative regulation of neuron death | 3.50E-03 |
| Chemokine signaling pathway | 3.51E-03 |
| HIF-1 signaling pathway | 3.51E-03 |
| Differentiation Pathway in PC12 Cells; this is a specific case of PAC1 Receptor Pathway. | 3.52E-03 |
| Activation of the AP-1 family of transcription factors | 3.52E-03 |
| RAF/MAP kinase cascade | 3.52E-03 |
| protein kinase activity | 3.54E-03 |
| Notch signaling pathway | 3.57E-03 |
| protein ubiquitination involved in ubiquitin-dependent protein catabolic process | 3.59E-03 |
| PI_Kinase | 3.61E-03 |
| appendage development | 3.62E-03 |
| response to estrogen | 3.62E-03 |
| limb development | 3.62E-03 |
| Presenilin action in Notch and Wnt signaling | 3.66E-03 |
| Integrins in angiogenesis | 3.66E-03 |
| mammary gland epithelium development | 3.69E-03 |
| positive regulation of type I interferon production | 3.69E-03 |
| MicroRNAs in cardiomyocyte hypertrophy | 3.75E-03 |
| Fc epsilon receptor (FCERI) signaling | 3.75E-03 |
| TNF signaling pathway | 3.82E-03 |
| TRIF-dependent toll-like receptor signaling pathway | 3.83E-03 |
| H4 histone acetyltransferase activity | 3.84E-03 |
| organic cyclic compound catabolic process | 3.84E-03 |
| EGF receptor signaling pathway | 3.93E-03 |
| mitotic G1/S transition checkpoint | 3.95E-03 |
| mitotic G1 DNA damage checkpoint | 3.95E-03 |
| Angiopoietin receptor Tie2-mediated signaling | 3.98E-03 |
| Type II diabetes mellitus | 3.98E-03 |
| Regulation of the Fanconi anemia pathway | 4.05E-03 |
| regulation of glial cell proliferation | 4.08E-03 |
| G1 DNA damage checkpoint | 4.08E-03 |
| positive regulation of protein polymerization | 4.08E-03 |
| protein serine/threonine kinase activity | 4.10E-03 |
| epithelial cell proliferation | 4.11E-03 |
| epidermis development | 4.11E-03 |
| glial cell differentiation | 4.18E-03 |
| MyD88-independent toll-like receptor signaling pathway | 4.20E-03 |
| Signaling by NOTCH | 4.23E-03 |
| regulation of myeloid cell differentiation | 4.25E-03 |
| Adenocarcinoma | 4.28E-03 |
| oligodendrocyte differentiation | 4.33E-03 |
| Regulation of Androgen receptor activity | 4.40E-03 |
| regulation of ossification | 4.41E-03 |
| transcription coactivator activity | 4.42E-03 |
| phosphatidylinositol 3-kinase complex | 4.44E-03 |
| Carcinoma, Renal Cell | 4.45E-03 |
| telomere maintenance via telomerase | 4.47E-03 |
| odontogenesis of dentin-containing tooth | 4.47E-03 |
| stem cell differentiation | 4.52E-03 |
| wound healing | 4.57E-03 |
| telomeric DNA binding | 4.70E-03 |
| Control of Gene Expression by Vitamin D Receptor | 4.73E-03 |
| Meiosis | 4.73E-03 |
| cellular response to light stimulus | 4.77E-03 |
| IL2-mediated signaling events | 4.85E-03 |
| regulation of protein ubiquitination involved in ubiquitin-dependent protein catabolic process | 4.89E-03 |
| regulation of anoikis | 4.89E-03 |
| toll-like receptor 3 signaling pathway | 4.89E-03 |
| nucleotide-excision repair | 4.89E-03 |
| MAPK | 4.92E-03 |
| MAP_kinase_CS | 4.92E-03 |
| Neurotrophin signaling pathway | 4.97E-03 |
| Glioblastoma | 5.00E-03 |
| Prostatic Neoplasms | 5.00E-03 |
| Intestinal Neoplasms | 5.00E-03 |
| FCERI mediated MAPK activation | 5.03E-03 |
| Fanconi anemia pathway | 5.03E-03 |
| protein acylation | 5.05E-03 |
| myeloid leukocyte differentiation | 5.05E-03 |
| cell adhesion molecule binding | 5.05E-03 |
| leukocyte activation | 5.06E-03 |
| proteolysis involved in cellular protein catabolic process | 5.06E-03 |
| JNK cascade | 5.11E-03 |
| positive regulation of cell cycle arrest | 5.18E-03 |
| Toxoplasmosis | 5.18E-03 |
| RANKL/RANK Signaling Pathway | 5.18E-03 |
| NFAT and Hypertrophy of the heart (Transcription in the broken heart) | 5.18E-03 |
| Signaling events mediated by focal adhesion kinase | 5.18E-03 |
| Oxidative Stress Induced Gene Expression Via Nrf2 | 5.20E-03 |
| Keap1-Nrf2 Pathway | 5.20E-03 |
| RB Tumor Suppressor/Checkpoint Signaling in response to DNA damage | 5.20E-03 |
| Regulation of actin cytoskeleton | 5.22E-03 |
| proteolysis | 5.25E-03 |
| peptidyl-lysine trimethylation | 5.28E-03 |
| DNA excision | 5.28E-03 |
| nucleotide-excision repair, DNA damage removal | 5.28E-03 |
| ATF-2 transcription factor network | 5.31E-03 |
| neuron part | 5.36E-03 |
| nuclear transcription factor complex | 5.36E-03 |
| vasculogenesis | 5.48E-03 |
| Regulation of retinoblastoma protein | 5.53E-03 |
| Non-small cell lung cancer | 5.53E-03 |
| positive regulation of transcription from RNA polymerase II promoter | 5.54E-03 |
| dendrite cytoplasm | 5.59E-03 |
| cell leading edge | 5.59E-03 |
| Axon guidance | 5.64E-03 |
| NOD-like receptor signaling pathway | 5.65E-03 |
| Acute myeloid leukemia | 5.65E-03 |
| Homologous recombination | 5.65E-03 |
| SOS-mediated signalling | 5.65E-03 |
| Cell to Cell Adhesion Signaling | 5.65E-03 |
| Presenilin action in Notch and Wnt signaling | 5.65E-03 |
| GRB2 events in EGFR signaling | 5.65E-03 |
| tongue development | 5.75E-03 |
| positive regulation of protein acetylation | 5.75E-03 |
| N-terminal protein amino acid modification | 5.75E-03 |
| osteoclast differentiation | 5.79E-03 |
| cellular protein catabolic process | 5.79E-03 |
| cell-cell junction organization | 5.79E-03 |
| B cell activation | 5.82E-03 |
| GLIOMA SUSCEPTIBILITY 1; GLM1 | 5.83E-03 |
| Fc-gamma receptor signaling pathway involved in phagocytosis | 5.92E-03 |
| immune response-regulating cell surface receptor signaling pathway involved in phagocytosis | 5.92E-03 |
| protein stabilization | 5.92E-03 |
| epithelial tube morphogenesis | 5.96E-03 |
| multi-organism reproductive process | 5.98E-03 |
| Ras protein signal transduction | 6.01E-03 |
| Cell-Cell communication | 6.02E-03 |
| cell junction assembly | 6.04E-03 |
| cellular macromolecular complex assembly | 6.04E-03 |
| Fc-gamma receptor signaling pathway | 6.04E-03 |
| Fc-epsilon receptor I signaling in mast cells | 6.05E-03 |
| glycoprotein biosynthetic process | 6.12E-03 |
| Wnt signaling pathway | 6.18E-03 |
| Hepatitis C | 6.20E-03 |
| SHC-mediated signalling | 6.20E-03 |
| the planar cell polarity Wnt signaling | 6.20E-03 |
| Hypoxia-Inducible Factor in the Cardiovascular System | 6.20E-03 |
| SHC1 events in EGFR signaling | 6.20E-03 |
| Signalling to p38 via RIT and RIN | 6.20E-03 |
| blood coagulation | 6.24E-03 |
| steroid hormone receptor binding | 6.28E-03 |
| T cell differentiation | 6.30E-03 |
| regulation of cell development | 6.32E-03 |
| coagulation | 6.38E-03 |
| vesicle-mediated transport | 6.41E-03 |
| regulation of neuron apoptotic process | 6.47E-03 |
| hemostasis | 6.47E-03 |
| skin development | 6.47E-03 |
| regionalization | 6.47E-03 |
| oocyte maturation | 6.51E-03 |
| regulation of histone deacetylation | 6.51E-03 |
| attachment of spindle microtubules to kinetochore | 6.51E-03 |
| Fc receptor mediated stimulatory signaling pathway | 6.61E-03 |
| phosphatidylinositol biosynthetic process | 6.61E-03 |
| regulation of multicellular organism growth | 6.61E-03 |
| Genes in module_403 | 6.64E-03 |
| Signaling by SCF-KIT | 6.76E-03 |
| Role of nicotinic acetylcholine receptors in the regulation of apoptosis | 6.76E-03 |
| p53 Signaling Pathway | 6.76E-03 |
| Homologous recombination repair of replication-independent double-strand breaks | 6.76E-03 |
| Homologous Recombination Repair | 6.76E-03 |
| ARMS-mediated activation | 6.76E-03 |
| p53 signaling pathway | 6.76E-03 |
| Oncostatin M Signaling Pathway | 6.76E-03 |
| NCAM signaling for neurite out-growth | 6.76E-03 |
| Wnt signaling pathway | 6.86E-03 |
| tube morphogenesis | 6.94E-03 |
| regulation of histone modification | 6.94E-03 |
| anoikis | 6.94E-03 |
| establishment or maintenance of apical/basal cell polarity | 6.94E-03 |
| positive regulation of collagen biosynthetic process | 6.94E-03 |
| establishment or maintenance of bipolar cell polarity | 6.94E-03 |
| RNA-dependent DNA replication | 6.94E-03 |
| TSH signaling pathway | 7.01E-03 |
| regulation of protein catabolic process | 7.08E-03 |
| regulation of cell migration | 7.09E-03 |
| protein C-terminus binding | 7.11E-03 |
| Insulin signaling pathway | 7.15E-03 |
| regulation of apoptotic signaling pathway | 7.16E-03 |
| anterior/posterior pattern specification | 7.16E-03 |
| Pancreatic Neoplasms | 7.25E-03 |
| regulation of cysteine-type endopeptidase activity | 7.27E-03 |
| Hemostasis | 7.29E-03 |
| Human Cytomegalovirus and Map Kinase Pathways | 7.29E-03 |
| PTEN is a tumor suppressor that dephosphorylates the lipid messenger phosphatidylinositol triphosphate. | 7.29E-03 |
| Tumor Suppressor Arf Inhibits Ribosomal Biogenesis | 7.29E-03 |
| IL-7 Signal Transduction | 7.29E-03 |
| Beta-catenin phosphorylation cascade | 7.29E-03 |
| regulation of hydrolase activity | 7.31E-03 |
| BCR signaling pathway | 7.36E-03 |
| positive regulation of collagen metabolic process | 7.38E-03 |
| regulation of odontogenesis | 7.38E-03 |
| regulation of protein export from nucleus | 7.38E-03 |
| BDNF signaling pathway | 7.40E-03 |
| Downstream signaling of activated FGFR | 7.40E-03 |
| regulation of smooth muscle cell proliferation | 7.41E-03 |
| DNA secondary structure binding | 7.47E-03 |
| Long-term potentiation | 7.51E-03 |
| DNA damage response | 7.51E-03 |
| Signaling events mediated by HDAC Class I | 7.51E-03 |
| Genes in module_57 | 7.53E-03 |
| ossification | 7.59E-03 |
| Cytosolic sensors of pathogen-associated DNA | 7.67E-03 |
| p53 signaling pathway | 7.67E-03 |
| SHC-related events | 7.67E-03 |
| Nongenotropic Androgen signaling | 7.67E-03 |
| Nerve growth factor pathway (NGF) | 7.67E-03 |
| Frs2-mediated activation | 7.67E-03 |
| SHC-related events triggered by IGF1R | 7.67E-03 |
| Role of Erk5 in Neuronal Survival | 7.67E-03 |
| regulation of Wnt signaling pathway | 7.67E-03 |
| muscle structure development | 7.69E-03 |
| positive regulation of sequence-specific DNA binding transcription factor activity | 7.76E-03 |
| negative regulation of G1/S transition of mitotic cell cycle | 7.76E-03 |
| negative regulation of cell cycle G1/S phase transition | 7.76E-03 |
| nucleobase-containing compound catabolic process | 7.81E-03 |
| transcription cofactor activity | 7.87E-03 |
| MAP kinase activity | 7.87E-03 |
| smooth muscle cell proliferation | 7.93E-03 |
| cellular response to reactive oxygen species | 7.93E-03 |
| immune response-activating signal transduction | 7.99E-03 |
| response to steroid hormone | 7.99E-03 |
| single-organism biosynthetic process | 8.02E-03 |
| cellular protein localization | 8.07E-03 |
| phosphatidylinositol phosphate kinase activity | 8.27E-03 |
| mitogen-activated protein kinase binding | 8.27E-03 |
| heart morphogenesis | 8.28E-03 |
| regulation of cell cycle arrest | 8.28E-03 |
| cellular macromolecule localization | 8.28E-03 |
| mammary gland epithelial cell proliferation | 8.28E-03 |
| regulation of glycolytic process | 8.28E-03 |
| TGF beta signaling pathway | 8.36E-03 |
| MAP00562 Inositol phosphate metabolism | 8.36E-03 |
| Tie2 Signaling | 8.36E-03 |
| Role of MAL in Rho-Mediated Activation of SRF | 8.36E-03 |
| TP53 network | 8.36E-03 |
| mitotic nuclear division | 8.44E-03 |
| cellular response to hypoxia | 8.44E-03 |
| Glioma | 8.62E-03 |
| Lung cancer | 8.62E-03 |
| LUNG CANCER | 8.62E-03 |
| Hemangiosarcoma | 8.62E-03 |
| Kidney Neoplasms | 8.62E-03 |
| Nasopharyngeal carcinoma | 8.62E-03 |
| Endometriosis | 8.62E-03 |
| Tracheoesophageal fistula | 8.62E-03 |
| Peripheral Nervous System Diseases | 8.62E-03 |
| Cell Transformation, Neoplastic | 8.62E-03 |
| Carcinoma, Transitional Cell | 8.62E-03 |
| toll-like receptor 4 signaling pathway | 8.64E-03 |
| regulation of chromatin organization | 8.64E-03 |
| B cell receptor signaling pathway | 8.67E-03 |
| cell junction organization | 8.68E-03 |
| Neoplasm Invasiveness | 8.72E-03 |
| meiotic chromosome segregation | 8.73E-03 |
| regulation of transcription from RNA polymerase II promoter in response to hypoxia | 8.73E-03 |
| neuron apoptotic process | 8.73E-03 |
| cellular response to decreased oxygen levels | 8.73E-03 |
| negative regulation of neurogenesis | 8.73E-03 |
| protein heterooligomerization | 8.73E-03 |
| dorsal/ventral pattern formation | 8.73E-03 |
| response to hydrogen peroxide | 8.73E-03 |
| cell-cell junction | 8.78E-03 |
| regulation of cell motility | 8.80E-03 |
| glycerolipid biosynthetic process | 8.80E-03 |
| regulation of type I interferon production | 8.88E-03 |
| regulation of proteolysis involved in cellular protein catabolic process | 8.88E-03 |
| regulation of leukocyte differentiation | 8.88E-03 |
| regulation of binding | 8.88E-03 |
| Liver Neoplasms, Experimental | 8.92E-03 |
| Adherens junction | 8.94E-03 |
| Endothelin signaling pathway | 8.94E-03 |
| sex chromosome | 8.94E-03 |
| Downstream signal transduction | 8.99E-03 |
| Prolonged ERK activation events | 8.99E-03 |
| Estrogen signaling pathway | 8.99E-03 |
| Signaling by constitutively active EGFR | 8.99E-03 |
| Lymphoma, Large B-Cell, Diffuse | 9.02E-03 |
| hair follicle morphogenesis | 9.18E-03 |
| regulation of osteoblast differentiation | 9.22E-03 |
| aromatic compound catabolic process | 9.22E-03 |
| epithelial cell differentiation | 9.22E-03 |
| negative regulation of epithelial cell proliferation | 9.22E-03 |
| type I interferon production | 9.22E-03 |
| regulation of glycoprotein biosynthetic process | 9.26E-03 |
| multicellular organismal aging | 9.26E-03 |
| growth hormone receptor signaling pathway | 9.26E-03 |
| maintenance of protein location in cell | 9.26E-03 |
| Signaling by FGFR | 9.32E-03 |
| Signaling by ERBB2 | 9.41E-03 |
| B Cell Receptor Signaling Pathway | 9.41E-03 |
| DAP12 signaling | 9.41E-03 |
| T cell activation | 9.43E-03 |
| Bacterial invasion of epithelial cells | 9.43E-03 |
| Formation of incision complex in GG-NER | 9.43E-03 |
| Type II diabetes mellitus | 9.43E-03 |
| IGF-1 Signaling Pathway | 9.43E-03 |
| SHC1 events in ERBB4 signaling | 9.43E-03 |
| Dual incision reaction in GG-NER | 9.43E-03 |
| Role of MEF2D in T-cell Apoptosis | 9.43E-03 |
| organ growth | 9.47E-03 |
| glycoprotein metabolic process | 9.47E-03 |
| establishment of organelle localization | 9.67E-03 |
| regulation of intracellular transport | 9.67E-03 |
| programmed necrotic cell death | 9.67E-03 |
| regulation of collagen biosynthetic process | 9.67E-03 |
| regulation of myelination | 9.67E-03 |
| regulation of Rac protein signal transduction | 9.67E-03 |
| sarcomere organization | 9.67E-03 |
| cellular response to growth hormone stimulus | 9.67E-03 |
| positive regulation of glial cell differentiation | 9.67E-03 |
| embryonic hindlimb morphogenesis | 9.67E-03 |
| cellular protein complex assembly | 9.71E-03 |
| cellular response to oxygen levels | 9.75E-03 |
| transcription regulatory region DNA binding | 9.76E-03 |
| transcription factor activity, transcription factor binding | 9.76E-03 |
| transcription factor activity, protein binding | 9.76E-03 |
| regulatory region DNA binding | 9.76E-03 |
| regulatory region nucleic acid binding | 9.76E-03 |
| regulation of Ras protein signal transduction | 9.89E-03 |
| N-acetyltransferase activity | 9.92E-03 |
| Insulin Signaling | 9.93E-03 |
| regulation of protein kinase B signaling | 9.96E-03 |
| Regulation of nuclear SMAD2/3 signaling | 1.00E-02 |
| core promoter binding | 1.00E-02 |
| cell cortex | 1.00E-02 |
| Signaling by Leptin | 1.00E-02 |
| Ceramide Signaling Pathway | 1.00E-02 |
| Apoptotic Signaling in Response to DNA Damage | 1.00E-02 |
| Aspirin Blocks Signaling Pathway Involved in Platelet Activation | 1.00E-02 |
| EBV LMP1 signaling | 1.00E-02 |
| Insulin Signaling Pathway | 1.00E-02 |
| G1/S transition of mitotic cell cycle | 1.01E-02 |
| liver development | 1.01E-02 |
| cell cycle G1/S phase transition | 1.01E-02 |
| proximal/distal pattern formation | 1.01E-02 |
| epithelial tube branching involved in lung morphogenesis | 1.01E-02 |
| MAPK signaling pathway | 1.03E-02 |
| Integrin signalling pathway | 1.05E-02 |
| SCF ubiquitin ligase complex | 1.05E-02 |
| occluding junction | 1.05E-02 |
| bicellular tight junction | 1.05E-02 |
| CTCF: First Multivalent Nuclear Factor | 1.06E-02 |
| GRB2 events in ERBB2 signaling | 1.06E-02 |
| Inhibition of Cellular Proliferation by Gleevec | 1.06E-02 |
| Multiple antiapoptotic pathways from IGF-1R signaling lead to BAD phosphorylation | 1.06E-02 |
| CCR3 signaling in Eosinophils | 1.06E-02 |
| VEGFR3 signaling in lymphatic endothelium | 1.06E-02 |
| Canonical NF-kappaB pathway | 1.06E-02 |
| hepaticobiliary system development | 1.06E-02 |
| negative regulation of homeostatic process | 1.07E-02 |
| regulation of glycoprotein metabolic process | 1.07E-02 |
| IRS-mediated signalling | 1.08E-02 |
| regulation of protein complex assembly | 1.10E-02 |
| intracellular steroid hormone receptor signaling pathway | 1.10E-02 |
| maintenance of location in cell | 1.12E-02 |
| positive regulation of multicellular organismal metabolic process | 1.12E-02 |
| epidermis morphogenesis | 1.12E-02 |
| regulation of collagen metabolic process | 1.12E-02 |
| negative regulation of gliogenesis | 1.12E-02 |
| S1P2 pathway | 1.13E-02 |
| Dorso-ventral axis formation | 1.13E-02 |
| Physiological and Pathological Hypertrophy of the Heart | 1.13E-02 |
| Circadian Repression of Expression by REV-ERBA | 1.13E-02 |
| Regulation of eIF4e and p70 S6 Kinase | 1.13E-02 |
| Signaling by FGFR in disease | 1.13E-02 |
| cellular response to external stimulus | 1.13E-02 |
| T cell activation | 1.13E-02 |
| NF-kappaB binding | 1.14E-02 |
| centrosome | 1.14E-02 |
| regulation of growth | 1.16E-02 |
| cardiocyte differentiation | 1.16E-02 |
| cellular response to lipopolysaccharide | 1.16E-02 |
| immune effector process | 1.17E-02 |
| IRS-related events | 1.17E-02 |
| stem cell development | 1.17E-02 |
| spinal cord motor neuron differentiation | 1.17E-02 |
| inner ear receptor cell development | 1.17E-02 |
| Signaling by EGFR | 1.17E-02 |
| IRS-related events triggered by IGF1R | 1.17E-02 |
| Apoptosis | 1.17E-02 |
| RORA Activates Circadian Expression | 1.17E-02 |
| Retinoic acid receptors-mediated signaling | 1.17E-02 |
| EGF receptor (ErbB1) signaling pathway | 1.17E-02 |
| SHC1 events in ERBB2 signaling | 1.17E-02 |
| Nectin adhesion pathway | 1.17E-02 |
| mCalpain and friends in Cell motility | 1.17E-02 |
| EGFR1 Signaling Pathway | 1.17E-02 |
| activation of immune response | 1.18E-02 |
| stem cell maintenance | 1.18E-02 |
| Carcinoma, Non-Small-Cell Lung | 1.19E-02 |
| Signaling by EGFR in Cancer | 1.19E-02 |
| RB in Cancer | 1.19E-02 |
| cadherin binding | 1.20E-02 |
| DAP12 interactions | 1.20E-02 |
| Signaling by PDGF | 1.20E-02 |
| Influenza A | 1.20E-02 |
| DNA-dependent DNA replication | 1.20E-02 |
| Regulation of BAD phosphorylation | 1.22E-02 |
| WNT Signaling Pathway | 1.22E-02 |
| The extracellular signal-regulated RAF/MEK/ERK signaling | 1.22E-02 |
| Nephrin/Neph1 signaling in the kidney podocyte | 1.22E-02 |
| Influence of Ras and Rho proteins on G1 to S Transition | 1.22E-02 |
| regulation of response to DNA damage stimulus | 1.22E-02 |
| histone H3-K4 methylation | 1.22E-02 |
| regulation of cellular carbohydrate catabolic process | 1.22E-02 |
| regulation of carbohydrate catabolic process | 1.22E-02 |
| IGF1R signaling cascade | 1.23E-02 |
| Signaling by Type 1 Insulin-like Growth Factor 1 Receptor (IGF1R) | 1.23E-02 |
| Genes related to Wnt-mediated signal transduction | 1.23E-02 |
| proteasome-mediated ubiquitin-dependent protein catabolic process | 1.23E-02 |
| kidney development | 1.23E-02 |
| positive regulation of protein complex assembly | 1.24E-02 |
| digestive tract development | 1.24E-02 |
| maintenance of protein location | 1.24E-02 |
| regulation of locomotion | 1.24E-02 |
| Insulin receptor signalling cascade | 1.25E-02 |
| Disease | 1.25E-02 |
| Genes related to IL4 rceptor signaling in B lymphocytes | 1.25E-02 |
| Regulation of Hypoxia-inducible Factor (HIF) by Oxygen | 1.25E-02 |
| 3-phosphoinositide biosynthesis | 1.25E-02 |
| Ephrin B reverse signaling | 1.25E-02 |
| Cellular response to hypoxia | 1.25E-02 |
| Myocyte Adrenergic Pathway is a specific case of the generalized Adrenergic Pathway. | 1.25E-02 |
| Signalling to RAS | 1.25E-02 |
| Transcription factor CREB and its extracellular signals | 1.25E-02 |
| Recycling pathway of L1 | 1.25E-02 |
| TGF-beta signaling pathway | 1.25E-02 |
| regulation of epithelial cell proliferation | 1.26E-02 |
| rhythmic process | 1.27E-02 |
| collagen biosynthetic process | 1.27E-02 |
| negative regulation of mitotic cell cycle | 1.27E-02 |
| embryonic limb morphogenesis | 1.28E-02 |
| cytokinesis | 1.28E-02 |
| embryonic appendage morphogenesis | 1.28E-02 |
| sequence-specific DNA binding | 1.28E-02 |
| cell-type specific apoptotic process | 1.28E-02 |
| GnRH signaling pathway | 1.29E-02 |
| perinuclear region of cytoplasm | 1.29E-02 |
| embryonic epithelial tube formation | 1.30E-02 |
| endothelial cell migration | 1.30E-02 |
| cellular response to molecule of bacterial origin | 1.30E-02 |
| response to growth hormone | 1.32E-02 |
| response to cocaine | 1.32E-02 |
| regulation of G1/S transition of mitotic cell cycle | 1.32E-02 |
| pallium development | 1.32E-02 |
| epithelial tube formation | 1.32E-02 |
| regulation of cellular component movement | 1.33E-02 |
| Interferon-gamma signaling pathway | 1.33E-02 |
| Growth Hormone Signaling Pathway | 1.33E-02 |
| regulation of cell cycle G1/S phase transition | 1.34E-02 |
| histone acetylation | 1.34E-02 |
| Adenocarcinoma of lung | 1.35E-02 |
| acetyltransferase activity | 1.36E-02 |
| N-acyltransferase activity | 1.36E-02 |
| apical junction complex | 1.36E-02 |
| regulation of transcription from RNA polymerase II promoter in response to stress | 1.37E-02 |
| necrotic cell death | 1.37E-02 |
| Inflammation mediated by chemokine and cytokine signaling pathway | 1.39E-02 |
| ubiquitin-dependent protein catabolic process | 1.40E-02 |
| Reelin signaling pathway | 1.40E-02 |
| VEGFR1 specific signals | 1.40E-02 |
| MAPK Cascade | 1.40E-02 |
| Links between Pyk2 and Map Kinases | 1.40E-02 |
| response to metal ion | 1.40E-02 |
| chemotaxis | 1.41E-02 |
| internal peptidyl-lysine acetylation | 1.41E-02 |
| taxis | 1.42E-02 |
| microtubule anchoring | 1.43E-02 |
| regulation of organelle assembly | 1.43E-02 |
| renal system development | 1.43E-02 |
| regulation of protein polymerization | 1.43E-02 |
| morphogenesis of an epithelium | 1.43E-02 |
| Toll Like Receptor 3 (TLR3) Cascade | 1.45E-02 |
| MyD88-independent cascade | 1.45E-02 |
| CD40/CD40L signaling | 1.46E-02 |
| Canonical Wnt signaling pathway | 1.46E-02 |
| Axon guidance mediated by netrin | 1.46E-02 |
| Dual incision reaction in TC-NER | 1.46E-02 |
| MAPK targets/ Nuclear events mediated by MAP kinases | 1.46E-02 |
| Formation of transcription-coupled NER (TC-NER) repair complex | 1.46E-02 |
| Cell surface interactions at the vascular wall | 1.46E-02 |
| protein localization | 1.48E-02 |
| hindlimb morphogenesis | 1.48E-02 |
| cardiac muscle cell proliferation | 1.48E-02 |
| digestive system development | 1.48E-02 |
| phosphatidylinositol metabolic process | 1.48E-02 |
| regulation of innate immune response | 1.48E-02 |
| intracellular receptor signaling pathway | 1.48E-02 |
| regulation of body fluid levels | 1.48E-02 |
| modification-dependent protein catabolic process | 1.48E-02 |
| regulation of neuron differentiation | 1.49E-02 |
| Myb_DNA-bd | 1.50E-02 |
| internal protein amino acid acetylation | 1.50E-02 |
| modification-dependent macromolecule catabolic process | 1.50E-02 |
| regulation of protein acetylation | 1.50E-02 |
| telomere maintenance via telomere lengthening | 1.50E-02 |
| hair cell differentiation | 1.50E-02 |
| response to cadmium ion | 1.50E-02 |
| cellular response to acid chemical | 1.50E-02 |
| mammary gland development | 1.50E-02 |
| tube formation | 1.50E-02 |
| negative regulation of neuron apoptotic process | 1.50E-02 |
| toll-like receptor signaling pathway | 1.50E-02 |
| response to acid chemical | 1.50E-02 |
| positive regulation of cytokine production | 1.51E-02 |
| positive regulation of epithelial cell differentiation | 1.51E-02 |
| cell cycle DNA replication | 1.51E-02 |
| thymus development | 1.51E-02 |
| positive regulation of protein ubiquitination | 1.51E-02 |
| ARM | 1.52E-02 |
| Estrogen signaling pathway | 1.53E-02 |
| CDO in myogenesis | 1.53E-02 |
| Myogenesis | 1.53E-02 |
| EGF Signaling Pathway | 1.53E-02 |
| Aurora A signaling | 1.53E-02 |
| Arm | 1.53E-02 |
| Myb_DNA-binding | 1.55E-02 |
| BROMODOMAIN_1 | 1.55E-02 |
| Bromodomain | 1.55E-02 |
| MYB_LIKE | 1.55E-02 |
| ARM_REPEAT | 1.55E-02 |
| BROMODOMAIN_2 | 1.55E-02 |
| BROMO | 1.55E-02 |
| Bromodomain | 1.55E-02 |
| Armadillo | 1.55E-02 |
| Bromodomain | 1.55E-02 |
| pattern specification process | 1.56E-02 |
| negative regulation of cell development | 1.56E-02 |
| skin morphogenesis | 1.56E-02 |
| positive regulation of neural precursor cell proliferation | 1.56E-02 |
| peptidyl-lysine methylation | 1.56E-02 |
| regulation of multicellular organismal metabolic process | 1.56E-02 |
| Wnt Signaling Pathway and Pluripotency | 1.56E-02 |
| NGF signalling via TRKA from the plasma membrane | 1.57E-02 |
| chromatin binding | 1.59E-02 |
| Regulation of toll-like receptor signaling pathway | 1.59E-02 |
| Ovarian Infertility Genes | 1.60E-02 |
| Oncogene Induced Senescence | 1.60E-02 |
| PDGF Signaling Pathway | 1.60E-02 |
| regulation of small GTPase mediated signal transduction | 1.61E-02 |
| Toll-like receptor signaling pathway | 1.61E-02 |
| ErbB1 downstream signaling | 1.61E-02 |
| maintenance of location | 1.62E-02 |
| regulation of DNA-templated transcription in response to stress | 1.62E-02 |
| striated muscle cell development | 1.64E-02 |
| Chagas disease (American trypanosomiasis) | 1.65E-02 |
| T cell receptor signaling pathway | 1.65E-02 |
| 3p21 | 1.66E-02 |
| EPHB forward signaling | 1.66E-02 |
| E-cadherin signaling in the nascent adherens junction | 1.66E-02 |
| Synthesis of PIPs at the plasma membrane | 1.66E-02 |
| Syndecan-2-mediated signaling events | 1.66E-02 |
| Fcgamma receptor (FCGR) dependent phagocytosis | 1.67E-02 |
| cellular response to biotic stimulus | 1.67E-02 |
| CD molecules | 1.67E-02 |
| membrane raft | 1.68E-02 |
| transcription-coupled nucleotide-excision repair | 1.68E-02 |
| cell adhesion mediated by integrin | 1.68E-02 |
| Toll-like receptor signaling pathway | 1.71E-02 |
| placenta development | 1.73E-02 |
| Wnt/beta-catenin Pathway | 1.73E-02 |
| HIF-2-alpha transcription factor network | 1.73E-02 |
| Global Genomic NER (GG-NER) | 1.73E-02 |
| CXCR3-mediated signaling events | 1.73E-02 |
| Senescence and Autophagy | 1.73E-02 |
| organelle localization | 1.74E-02 |
| somatic stem cell maintenance | 1.74E-02 |
| ventral spinal cord development | 1.74E-02 |
| myofibril assembly | 1.74E-02 |
| Rap1 signaling pathway | 1.80E-02 |
| Signal transduction by L1 | 1.80E-02 |
| CARM1 and Regulation of the Estrogen Receptor | 1.80E-02 |
| IL1-mediated signaling events | 1.80E-02 |
| regulation of cytokinesis | 1.81E-02 |
| lipopolysaccharide-mediated signaling pathway | 1.81E-02 |
| negative regulation of chromosome organization | 1.81E-02 |
| dendrite | 1.82E-02 |
| cellular response to organic cyclic compound | 1.82E-02 |
| cell maturation | 1.83E-02 |
| leukocyte migration | 1.84E-02 |
| Mouth Neoplasms | 1.85E-02 |
| Signaling events mediated by TCPTP | 1.87E-02 |
| bicellular tight junction assembly | 1.87E-02 |
| striated muscle cell proliferation | 1.87E-02 |
| positive regulation of I-kappaB kinase/NF-kappaB signaling | 1.89E-02 |
| PI3K-Akt signaling pathway | 1.91E-02 |
| Cholinergic synapse | 1.93E-02 |
| Signaling by Insulin receptor | 1.93E-02 |
| ALK in cardiac myocytes | 1.93E-02 |
| BMAL1:CLOCK/NPAS2 Activates Circadian Expression | 1.93E-02 |
| G alpha 13 Pathway | 1.93E-02 |
| Signalling to ERKs | 1.93E-02 |
| positive regulation of myeloid leukocyte differentiation | 1.93E-02 |
| Rac protein signal transduction | 1.93E-02 |
| protein export from nucleus | 1.93E-02 |
| morphogenesis of embryonic epithelium | 1.94E-02 |
| cellular homeostasis | 1.94E-02 |
| SANT | 1.95E-02 |
| androgen receptor binding | 1.95E-02 |
| muscle cell development | 1.97E-02 |
| somatodendritic compartment | 1.98E-02 |
| positive regulation of histone modification | 1.99E-02 |
| response to fatty acid | 1.99E-02 |
| spindle assembly | 1.99E-02 |
| neuron projection | 2.00E-02 |
| SANT_DNA-bd | 2.01E-02 |
| FBOX | 2.01E-02 |
| positive regulation of immune system process | 2.02E-02 |
| urogenital system development | 2.02E-02 |
| regulation of cellular localization | 2.02E-02 |
| negative regulation of molecular function | 2.02E-02 |
| prostate gland development | 2.02E-02 |
| artery morphogenesis | 2.02E-02 |
| single organismal cell-cell adhesion | 2.02E-02 |
| small GTPase mediated signal transduction | 2.02E-02 |
| negative regulation of protein binding | 2.03E-02 |
| centrosome cycle | 2.03E-02 |
| histone H3 acetylation | 2.03E-02 |
| IL-2 Receptor Beta Chain in T cell Activation | 2.03E-02 |
| ubiquitin-protein transferase activity | 2.04E-02 |
| estrogen receptor binding | 2.04E-02 |
| nuclear hormone receptor binding | 2.04E-02 |
| Signaling by Interleukins | 2.04E-02 |
| positive regulation of immune response | 2.06E-02 |
| sensory organ development | 2.07E-02 |
| response to reactive oxygen species | 2.08E-02 |
| regulation of transport | 2.08E-02 |
| FRS2-mediated cascade | 2.12E-02 |
| Leukocyte transendothelial migration | 2.12E-02 |
| Ras signaling pathway | 2.12E-02 |
| endonuclease activity | 2.13E-02 |
| exocytosis | 2.13E-02 |
| lipid modification | 2.14E-02 |
| lateral plasma membrane | 2.14E-02 |
| cell differentiation in spinal cord | 2.16E-02 |
| identical protein binding | 2.16E-02 |
| cellular response to oxidative stress | 2.16E-02 |
| Endometrial Neoplasms | 2.18E-02 |
| intercalated disc | 2.19E-02 |
| glycerolipid metabolic process | 2.19E-02 |
| pattern recognition receptor signaling pathway | 2.19E-02 |
| B Cell Antigen Receptor | 2.21E-02 |
| kidney morphogenesis | 2.22E-02 |
| lung morphogenesis | 2.22E-02 |
| positive regulation of cell adhesion | 2.22E-02 |
| myelin sheath | 2.24E-02 |
| Nerve Degeneration | 2.25E-02 |
| innate immune response-activating signal transduction | 2.28E-02 |
| regulation of Notch signaling pathway | 2.28E-02 |
| apical junction assembly | 2.28E-02 |
| inner ear receptor cell differentiation | 2.28E-02 |
| F-box_dom_Skp2-like | 2.33E-02 |
| Neoplasm Metastasis | 2.33E-02 |
| positive regulation of DNA replication | 2.35E-02 |
| regulation of intracellular steroid hormone receptor signaling pathway | 2.35E-02 |
| histone deacetylase complex | 2.37E-02 |
| nuclear body | 2.41E-02 |
| branching morphogenesis of an epithelial tube | 2.41E-02 |
| regulation of stress-activated MAPK cascade | 2.41E-02 |
| central nervous system neuron differentiation | 2.41E-02 |
| cell-cell signaling | 2.41E-02 |
| artery development | 2.41E-02 |
| cardiac muscle tissue growth | 2.41E-02 |
| positive regulation of response to DNA damage stimulus | 2.41E-02 |
| Cell Cycle Checkpoints | 2.41E-02 |
| epithelial cell migration | 2.42E-02 |
| epithelium migration | 2.42E-02 |
| regulation of stress-activated protein kinase signaling cascade | 2.42E-02 |
| epidermal cell differentiation | 2.42E-02 |
| negative regulation of intracellular signal transduction | 2.43E-02 |
| cartilage development | 2.46E-02 |
| positive regulation of canonical Wnt signaling pathway | 2.46E-02 |
| embryonic digit morphogenesis | 2.46E-02 |
| cardiac muscle cell development | 2.46E-02 |
| response to dsRNA | 2.46E-02 |
| retroviral 3' processing activity | 2.47E-02 |
| retroviral integrase activity | 2.47E-02 |
| T/G mismatch-specific endonuclease activity | 2.47E-02 |
| integrase activity | 2.47E-02 |
| hormone receptor binding | 2.47E-02 |
| response to carbohydrate | 2.47E-02 |
| activation of innate immune response | 2.47E-02 |
| cardiac muscle tissue development | 2.47E-02 |
| endocytosis | 2.48E-02 |
| adherens junction assembly | 2.48E-02 |
| repressing transcription factor binding | 2.49E-02 |
| double-stranded RNA binding | 2.49E-02 |
| striated muscle tissue development | 2.49E-02 |
| tissue migration | 2.49E-02 |
| protein polyubiquitination | 2.49E-02 |
| synaptic transmission | 2.49E-02 |
| positive regulation of smooth muscle cell proliferation | 2.51E-02 |
| Oxidative Stress Induced Senescence | 2.51E-02 |
| Colonic Neoplasms | 2.52E-02 |
| Bioactive Peptide Induced Signaling Pathway | 2.52E-02 |
| side of membrane | 2.53E-02 |
| cytoplasmic side of plasma membrane | 2.53E-02 |
| cell-cell contact zone | 2.53E-02 |
| replication fork | 2.54E-02 |
| RNA polymerase II transcription factor complex | 2.54E-02 |
| PDGF signaling pathway | 2.55E-02 |
| Arf6 trafficking events | 2.57E-02 |
| Ceramide signaling pathway | 2.57E-02 |
| PLK1 signaling events | 2.57E-02 |
| Regulation of Microtubule Cytoskeleton | 2.57E-02 |
| outflow tract morphogenesis | 2.58E-02 |
| mechanoreceptor differentiation | 2.58E-02 |
| actomyosin structure organization | 2.58E-02 |
| androgen receptor signaling pathway | 2.58E-02 |
| F-box | 2.58E-02 |
| F-box_dom_cyclin-like | 2.58E-02 |
| ERK1 and ERK2 cascade | 2.62E-02 |
| Neighborhood of RRM1 | 2.63E-02 |
| Neighborhood of HDAC2 | 2.63E-02 |
| Genes in module_197 | 2.63E-02 |
| toll-like receptor 10 signaling pathway | 2.65E-02 |
| cardiac cell development | 2.65E-02 |
| G2/M Checkpoints | 2.67E-02 |
| T Cell Signal Transduction | 2.67E-02 |
| Signaling by FGFR mutants | 2.67E-02 |
| tissue morphogenesis | 2.68E-02 |
| regulation of nucleocytoplasmic transport | 2.68E-02 |
| protein polymerization | 2.68E-02 |
| organelle assembly | 2.70E-02 |
| positive regulation of muscle cell differentiation | 2.71E-02 |
| cellular carbohydrate catabolic process | 2.71E-02 |
| glycerophospholipid biosynthetic process | 2.71E-02 |
| FBOX | 2.74E-02 |
| Osteoclast differentiation | 2.75E-02 |
| Members of the BCR signaling pathway | 2.75E-02 |
| Keratinocyte Differentiation | 2.75E-02 |
| Transcription-coupled NER (TC-NER) | 2.75E-02 |
| canonical Wnt signaling | 2.75E-02 |
| embryonic morphogenesis | 2.76E-02 |
| heart growth | 2.77E-02 |
| activation of MAPKK activity | 2.77E-02 |
| toll-like receptor 5 signaling pathway | 2.77E-02 |
| regulation of endopeptidase activity | 2.77E-02 |
| p53 binding | 2.82E-02 |
| ubiquitin ligase complex | 2.82E-02 |
| Tight junction | 2.82E-02 |
| T Cell Receptor Signaling Pathway | 2.82E-02 |
| FGF signaling pathway | 2.82E-02 |
| Heart Development | 2.82E-02 |
| Nucleotide excision repair | 2.82E-02 |
| positive regulation of catabolic process | 2.84E-02 |
| muscle tissue development | 2.85E-02 |
| Natural killer cell mediated cytotoxicity | 2.85E-02 |
| single organism cell adhesion | 2.87E-02 |
| glycolytic process | 2.91E-02 |
| response to ammonium ion | 2.91E-02 |
| Notch-mediated HES/HEY network | 2.92E-02 |
| Notch signaling pathway | 2.92E-02 |
| epithelial cell development | 2.92E-02 |
| forebrain development | 2.92E-02 |
| somitogenesis | 2.92E-02 |
| DNA methylation or demethylation | 2.92E-02 |
| cellular response to mechanical stimulus | 2.92E-02 |
| Signal Transduction | 2.96E-02 |
| neuron projection guidance | 2.98E-02 |
| axon guidance | 2.98E-02 |
| response to osmotic stress | 2.98E-02 |
| toll-like receptor TLR6:TLR2 signaling pathway | 2.98E-02 |
| toll-like receptor TLR1:TLR2 signaling pathway | 2.98E-02 |
| cytoplasmic side of membrane | 2.99E-02 |
| MAP kinase activation in TLR cascade | 3.02E-02 |
| macromolecule methylation | 3.04E-02 |
| regulation of cysteine-type endopeptidase activity involved in apoptotic process | 3.04E-02 |
| cardiac ventricle morphogenesis | 3.04E-02 |
| regulation of biomineral tissue development | 3.04E-02 |
| peptidyl-threonine phosphorylation | 3.04E-02 |
| osteoblast differentiation | 3.06E-02 |
| telencephalon development | 3.06E-02 |
| positive regulation of MAP kinase activity | 3.06E-02 |
| phagocytosis | 3.06E-02 |
| MAPK signaling pathway | 3.06E-02 |
| protein autophosphorylation | 3.09E-02 |
| DNA catabolic process, endonucleolytic | 3.11E-02 |
| Downstream signaling in naive CD8+ T cells | 3.12E-02 |
| regulation of peptidase activity | 3.15E-02 |
| Axon guidance | 3.17E-02 |
| Nucleotide Excision Repair | 3.21E-02 |
| Role of Calcineurin-dependent NFAT signaling in lymphocytes | 3.21E-02 |
| Genes related to the insulin receptor pathway | 3.21E-02 |
| PI Metabolism | 3.21E-02 |
| presynaptic membrane | 3.22E-02 |
| regulation of phosphatidylinositol 3-kinase signaling | 3.26E-02 |
| toll-like receptor 9 signaling pathway | 3.26E-02 |
| histone methyltransferase complex | 3.27E-02 |
| cellular component assembly involved in morphogenesis | 3.28E-02 |
| morphogenesis of a branching epithelium | 3.28E-02 |
| Signaling events mediated by Stem cell factor receptor (c-Kit) | 3.32E-02 |
| regulation of cell morphogenesis | 3.32E-02 |
| in utero embryonic development | 3.32E-02 |
| peptidyl-threonine modification | 3.32E-02 |
| regulation of generation of precursor metabolites and energy | 3.32E-02 |
| toll-like receptor 2 signaling pathway | 3.34E-02 |
| negative regulation of response to stimulus | 3.36E-02 |
| nucleocytoplasmic transport | 3.40E-02 |
| positive regulation of protein kinase B signaling | 3.40E-02 |
| regulation of neural precursor cell proliferation | 3.40E-02 |
| Melanoma | 3.40E-02 |
| Endothelins | 3.43E-02 |
| positive regulation of myeloid cell differentiation | 3.48E-02 |
| negative regulation of cysteine-type endopeptidase activity involved in apoptotic process | 3.48E-02 |
| cell projection part | 3.49E-02 |
| positive regulation of innate immune response | 3.50E-02 |
| Cell Cycle, Mitotic | 3.52E-02 |
| nuclear transport | 3.52E-02 |
| regulation of cell projection organization | 3.52E-02 |
| phospholipid biosynthetic process | 3.53E-02 |
| response to cytokine | 3.53E-02 |
| T cell differentiation in thymus | 3.53E-02 |
| regulation of organ growth | 3.53E-02 |
| positive regulation of cell division | 3.53E-02 |
| pyrimidine-containing compound metabolic process | 3.53E-02 |
| centrosome organization | 3.53E-02 |
| Stabilization of p53 | 3.54E-02 |
| protein O-linked glycosylation | 3.60E-02 |
| glial cell development | 3.60E-02 |
| histone lysine methylation | 3.60E-02 |
| somite development | 3.60E-02 |
| core promoter sequence-specific DNA binding | 3.61E-02 |
| beta-catenin binding | 3.61E-02 |
| transcriptional repressor complex | 3.61E-02 |
| basolateral plasma membrane | 3.61E-02 |
| RAC1 signaling pathway | 3.64E-02 |
| Basal cell carcinoma | 3.64E-02 |
| TGF Beta Signaling Pathway | 3.64E-02 |
| organonitrogen compound catabolic process | 3.64E-02 |
| nuclease activity | 3.64E-02 |
| SMAD binding | 3.64E-02 |
| transferase activity, transferring acyl groups other than amino-acyl groups | 3.64E-02 |
| response to inorganic substance | 3.65E-02 |
| morphogenesis of a branching structure | 3.66E-02 |
| negative regulation of cysteine-type endopeptidase activity | 3.67E-02 |
| negative regulation of protein metabolic process | 3.67E-02 |
| regulation of intracellular protein transport | 3.68E-02 |
| regulation of I-kappaB kinase/NF-kappaB signaling | 3.68E-02 |
| Neurotrophic factor-mediated Trk receptor signaling | 3.75E-02 |
| Circadian Clock | 3.75E-02 |
| MyD88-dependent toll-like receptor signaling pathway | 3.75E-02 |
| secretion by cell | 3.76E-02 |
| TGF-beta Receptor Signaling Pathway | 3.79E-02 |
| Coregulation of Androgen receptor activity | 3.79E-02 |
| nucleic acid phosphodiester bond hydrolysis | 3.82E-02 |
| response to heat | 3.82E-02 |
| microtubule organizing center organization | 3.82E-02 |
| platelet activation | 3.85E-02 |
| chromatin DNA binding | 3.86E-02 |
| 8q24.3 | 3.88E-02 |
| immune response-activating cell surface receptor signaling pathway | 3.89E-02 |
| cell-cell junction assembly | 3.89E-02 |
| regulation of cellular amine metabolic process | 3.89E-02 |
| DNA modification | 3.89E-02 |
| carbohydrate metabolic process | 3.90E-02 |
| connective tissue development | 3.92E-02 |
| Esophageal Squamous Cell Carcinoma | 3.94E-02 |
| establishment of synaptic vesicle localization | 3.96E-02 |
| synaptic vesicle transport | 3.96E-02 |
| histone acetyltransferase complex | 3.98E-02 |
| histone deacetylase binding | 3.98E-02 |
| p53-Dependent G1 DNA Damage Response | 4.00E-02 |
| p53-Dependent G1/S DNA damage checkpoint | 4.00E-02 |
| negative regulation of protein modification process | 4.02E-02 |
| regulation of protein serine/threonine kinase activity | 4.04E-02 |
| Long-term depression | 4.09E-02 |
| mTOR signaling pathway | 4.09E-02 |
| Leptin signaling pathway | 4.09E-02 |
| Wnt Signaling Pathway | 4.09E-02 |
| regulation of striated muscle cell differentiation | 4.09E-02 |
| synaptic vesicle localization | 4.11E-02 |
| positive regulation of Wnt signaling pathway | 4.11E-02 |
| pancreas development | 4.11E-02 |
| protein localization to organelle | 4.13E-02 |
| axis specification | 4.18E-02 |
| establishment of cell polarity | 4.18E-02 |
| spinal cord development | 4.18E-02 |
| p75(NTR)-mediated signaling | 4.19E-02 |
| Shigellosis | 4.19E-02 |
| Inositol phosphate metabolism | 4.19E-02 |
| PROTEIN_KINASE_ATP | 4.21E-02 |
| phosphoric ester hydrolase activity | 4.22E-02 |
| glycerophospholipid metabolic process | 4.28E-02 |
| G1/S DNA Damage Checkpoints | 4.31E-02 |
| developmental maturation | 4.32E-02 |
| synapse | 4.32E-02 |
| methyltransferase complex | 4.32E-02 |
| cell junction | 4.32E-02 |
| negative regulation of canonical Wnt signaling pathway | 4.34E-02 |
| regulation of reproductive process | 4.34E-02 |
| ARM-like | 4.34E-02 |
| ARM-like | 4.34E-02 |
| negative regulation of signal transduction | 4.35E-02 |
| chemical homeostasis | 4.37E-02 |
| response to lipopolysaccharide | 4.42E-02 |
| segmentation | 4.42E-02 |
| I-kappaB kinase/NF-kappaB signaling | 4.42E-02 |
| acetyltransferase complex | 4.45E-02 |
| protein acetyltransferase complex | 4.45E-02 |
| cerebral cortex development | 4.45E-02 |
| pyruvate metabolic process | 4.45E-02 |
| phosphatidylinositol 3-kinase signaling | 4.45E-02 |
| columnar/cuboidal epithelial cell differentiation | 4.45E-02 |
| Neighborhood of BUB1 | 4.48E-02 |
| endodeoxyribonuclease activity | 4.59E-02 |
| protease binding | 4.59E-02 |
| enzyme regulator activity | 4.59E-02 |
| perikaryon | 4.59E-02 |
| carbohydrate derivative catabolic process | 4.61E-02 |
| Alzheimer disease-amyloid secretase pathway | 4.63E-02 |
| Fas Signaling Pathway | 4.63E-02 |
| 17p11.2 | 4.64E-02 |
| skeletal system development | 4.70E-02 |
| ameboidal-type cell migration | 4.70E-02 |
| regulation of cell-cell adhesion | 4.70E-02 |
| cardiac muscle cell differentiation | 4.70E-02 |
| Downstream Signaling Events Of B Cell Receptor (BCR) | 4.71E-02 |
| Wnt signaling pathway | 4.71E-02 |
| DNA Damage/Telomere Stress Induced Senescence | 4.72E-02 |
| AGE/RAGE pathway | 4.72E-02 |
| histone methylation | 4.75E-02 |
| spindle organization | 4.75E-02 |
| adherens junction organization | 4.75E-02 |
| cellular component disassembly | 4.75E-02 |
| regulation of establishment of protein localization | 4.75E-02 |
| negative regulation of binding | 4.75E-02 |
| protein domain specific binding | 4.77E-02 |
| Genes related to PIP3 signaling in cardiac myocytes | 4.81E-02 |
| Kit Receptor Signaling Pathway | 4.81E-02 |
| Cyclin A:Cdk2-associated events at S phase entry | 4.81E-02 |
| superpathway of inositol phosphate compounds | 4.81E-02 |
| DNA-dependent ATPase activity | 4.88E-02 |
| G1 to S cell cycle control | 4.89E-02 |
| transferase activity, transferring acyl groups | 4.91E-02 |
| Prot_kinase_cat_dom | 4.95E-02 |
| Degradation of beta-catenin by the destruction complex | 4.97E-02 |
| regulation of cell morphogenesis involved in differentiation | 5.00E-02 |
| positive regulation of transport | 5.00E-02 |
